# Diagnostic accuracy, fairness and clinical implementation of AI for breast cancer screening: results of multicenter retrospective and prospective technical feasibility studies

**DOI:** 10.1038/s43018-026-01127-0

**Published:** 2026-03-10

**Authors:** Christopher J. Kelly, Marc Wilson, Lucy M. Warren, Richard Sidebottom, Mark Halling-Brown, Lin Yang, Megumi Morigami, Jenny Venton, Reena Chopra, Jane Chang, Jonathan Dixon, Fiona J. Gilbert, Daniel I. Golden, Elzbieta Gruzewska, Lesley Honeyfield, Amandeep Hujan, Delara Khodabakhshi, Emma Lewis, Namrata Malhotra, Rachita Mallya, Della Ogunleye, Charlotte Purdy, Rory Sayres, Marcin Sieniek, Tsvetina Stoycheva, Aminata Sy, Susan Thomas, Dominic Ward, Lihong Xi, Shawn Xu, Shravya Shetty, Ara Darzi, Kenneth Young, Hema Purushothaman, Lisanne Khoo, Mamatha Reddy, Hutan Ashrafian, Deborah Cunningham

**Affiliations:** 1 Google Research, Mountain View, CA USA; 2 Royal Surrey NHS Foundation Trust, Guildford, UK; 3 The Royal Marsden NHS Foundation Trust, London, UK; 4 University of Surrey, Guildford, UK; 5 University of Cambridge, Cambridge, UK; 6 Imperial College Healthcare NHS Trust, London, UK; 7 St George’s University Hospitals NHS Foundation Trust, London, UK; 8 Imperial College London, London, UK; 9 AIMS Public Engagement Group, London, UK

**Keywords:** Cancer, Diagnosis, Machine learning

## Abstract

Artificial intelligence (AI) promises to enhance breast cancer screening. Here we evaluated Google’s mammography AI system (version 1.2) across two phases: a retrospective study using 115,973 mammograms from five National Health Service screening services with 39-month follow-up and prospective noninterventional feasibility deployment at 12 sites (9,266 cases). The primary endpoint was AI sensitivity and specificity versus first reader using a 5% noninferiority margin. The secondary endpoints were performance versus second or consensus readers and breast-level analyses. Retrospectively, AI achieved superior sensitivity (0.541 versus 0.437 for first reader, *P* < 0.001) and noninferior specificity (0.943 versus 0.952, *P* < 0.001). Cancer detection rate increased from 7.54 to 9.33 per 1,000 women, with AI detecting 25.0% of interval cancers. Performance was particularly strong for first screens (39.3% fewer recalls, 8.8% higher detection) and invasive cancers. No systematic demographic disparities were observed. Simulated second-reader replacement reduced reading time by 32% while increasing detection by 17.7%. Prospective deployment confirmed technical feasibility but revealed a distribution shift requiring threshold recalibration. Implementation requires adaptive calibration and continuous monitoring to ensure safety and equity.

## Abstract (web summary)

Kelly et al. assessed an artificial intelligence system for breast cancer screening in retrospective datasets, followed by prospective feasibility evaluation, and report its accuracy, fairness and clinical implementation in multiple workflow settings.

## Main

Breast cancer screening offers a compelling clinical use case for artificial intelligence (AI) in healthcare. It is hoped that AI can improve the quality and consistency of screening while improving cost effectiveness and addressing global radiologist workforce shortages. A number of retrospective studies have demonstrated that AI can perform at least on a par with specialists in different settings^1^ and new evidence from interventional studies is growing^2–4^. Despite this, the use of modern AI in breast screening programs has not been widely adopted to date.

Several crucial pieces of evidence that thoroughly assess benefits and harms are still required to catalyze widespread adoption^5^. These include a better understanding of the spectrum of disease detected by AI, understanding the impact on interval cancers in large and diverse populations, ensuring safety and equity, careful exploration of human–computer interaction and implementation science to understand practical considerations when deploying AI at scale.

Global breast screening workflows can be divided into single-read (primarily United States) and double-read (other countries) workflows^6^. Double-read screening involves two readers separately reviewing each mammogram, where the second reader can be blinded or unblinded to the first reader’s decision. An arbitration panel is invoked in cases of reader disagreement and sometimes for all suspected cancer cases. In the double-read setting, there are at least three potential AI deployment options: (1) AI assisting readers^7^; (2) AI as an independent reader^4,8^; or (3) AI triage to one or two readers^3^.

**Fig. 1:**
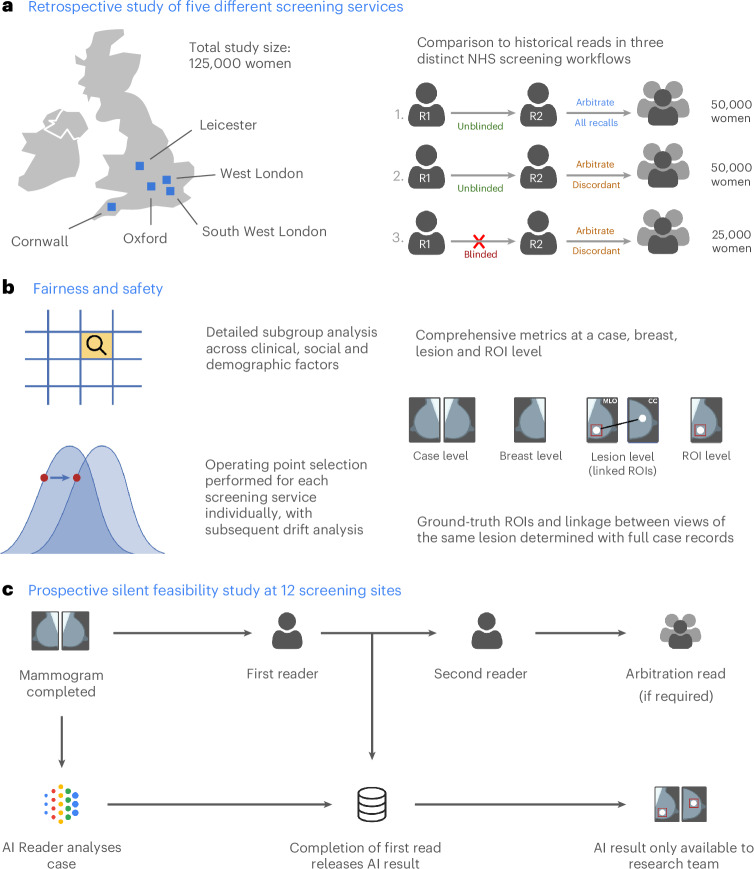
Overview of study. **a**, First, we completed a large evaluation of an AI system using a comprehensive set of metrics across five different screening services and three distinct workflows on the basis of whether the second reader is blinded to the first read and how cases are selected for arbitration. **b**, We assessed the system for fairness across many subgroups of interest and assessed the stability of operation point selection given clinical or technical variation between settings. **c**, We performed a detailed workflow assessment and prospective silent feasibility study at two screening services, covering 12 screening sites.

**Extended Data Fig. 1:**
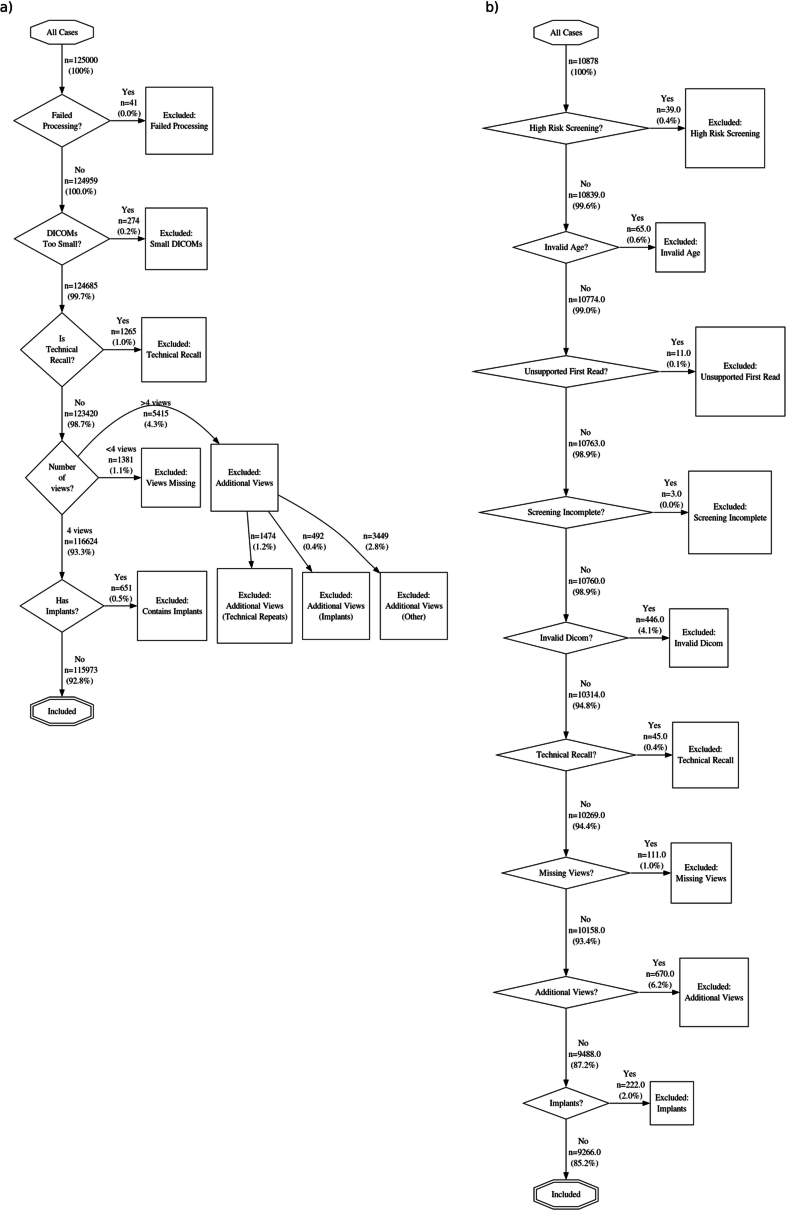
Data flow diagram. (**a**) Data flow diagram for the retrospective study showing flow across all five services, demonstrating reasons for all exclusions. A small number of cases (n = 41 participants, 0.0328%) failed processing for a variety of tail reasons. Some DICOMs (n = 274 participants, 0.22%) were much smaller than typical screening images, reflecting a rare deviation from standard imaging resolution. Technical recalls (n = 1265 participants, 1.0%) were excluded as these would be repeated in clinical practice. (**b**) Data flow diagram showing prospective study flow across both services, including reasons for all exclusions. 43 women opted out of participating in the study, and are excluded from the entire dataflow diagram.

We previously described an AI system that performed at least equivalently to UK and US radiologists when predicting breast cancer from screening mammograms^9^. In this translational study, we present our onward steps to develop an AI independent reader for a double-reader workflow, retrospective evaluation across five UK screening services (Fig. 1a and Extended Data Fig. 1a), evaluations of bias and fairness, early health economic assessment (Fig. 1b) and the clinical, operational and technical insights that were gained from prospective observational deployment at two screening services (Fig. 1c and Extended Data Fig. 1b).

## Results

### Retrospective standalone evaluation

The retrospective evaluation covered five breast screening services from across the UK, representing three distinct clinical workflows, including 125,000 women aged 50–70, who were screened in 2015–2016, as summarized in Table 1. The final analysis included 115,973 women after applying inclusion and exclusion criteria (Extended Data Fig. 1a and Supplementary Table 1).

**Table 1:** Summary characteristics of the five screening services and their datasets included in the study

Screening service	All services	Service 1	Service 2	Service 3	Service 4	Service 5
**Arbitration style during study**	Mix of practices	Discordant only	Arbitrate all recalls	Discordant only	Discordant only	Arbitrate all recalls
**Blinded or unblinded reader 1 or 2**	Mix of practices	Unblinded	Unblinded	Unblinded	Blinded	Unblinded
**Total women,** ***n***	125,000	25,000	25,000	25,000	25,000	25,000
**Included,** ***n*** **(%)**	115,973 (92.8%)	23,023 (92.1%)	22,630 (90.5%)	23,400 (93.6%)	23,590 (94.4%)	23,330 (93.3%)
**Of those included**						
**Normal,** ***n*** **(%)**	113,972 (98.3%)	22,674 (98.5%)	22,248 (98.3%)	23,004 (98.3%)	23,135 (98.1%)	22,911 (98.2%)
**All cancers,** ***n*** **(%)**	2,001 (1.7%)	349 (1.5%)	382 (1.7%)	396 (1.7%)	455 (1.9%)	419 (1.8%)
**Screen detected,** ***n*** **(%)**	876 (0.8%)	169 (0.7%)	191 (0.8%)	178 (0.8%)	186 (0.8%)	152 (0.7%)
**Interval cancers,** ***n*** **(%)**	336 (0.3%)	68 (0.3%)	68 (0.3%)	64 (0.3%)	65 (0.3%)	71 (0.3%)
**Next screen detected,** ***n*** **(%)**	789 (0.7%)	112 (0.5%)	123 (0.5%)	154 (0.7%)	204 (0.9%)	196 (0.8%)
**Screening recall rate (%, 95% CI)**	4.0 (3.9, 4.1)	4.9 (4.6, 5.2)	4.4 (4.2, 4.7)	4.0 (3.7, 4.3)	3.2 (3.0, 3.4)	3.3 (3.1, 3.5)
Arbitration rate (%) **	N/A	3.9%	12.1%	2.7%	3.4%	5.6%
Direct to clinic (%) **	N/A	3.2%	N/A	3.1%	2.3%	N/A
**CDR per 1,000 women (95% CI)**	8.03 (7.51, 8.54)	8.84 (7.62, 10.06)	7.60 (6.48, 8.72)	8.12 (6.97, 9.27)	8.48 (7.31, 9.65)	7.12 (6.04, 8.19)
Age *	59 (50–70)	58 (50–70)	58 (50–70)	60 (50–70)	59 (50–70)	59 (50–70)
**Equipment manufacturer**	Hologic: 71%, Siemens: 22%, GE: 7%	Hologic: 100%	Hologic: 88%, Siemens: 10%, GE: 1%	Siemens: 100%	Hologic: 100%	Hologic: 68%, GE: 32%

The screening interval in the UK is 3 years. Cancer outcomes were defined using a 39-month follow-up window following the index screen. *Median (range). **Percentages are directly influenced by arbitration style and are not easily comparable across sites with different styles. ‘Arbitration style’ determines which cases undergo panel review: ‘discordant’ sends only cases where readers disagree, whilst ‘arbitrate all’ sends any case flagged by either reader. ‘Direct to clinic’ describes cases that go straight to clinic without arbitration.

Source data

### AI achieved superior sensitivity and noninferior specificity for cancer detection

**Fig. 2:**
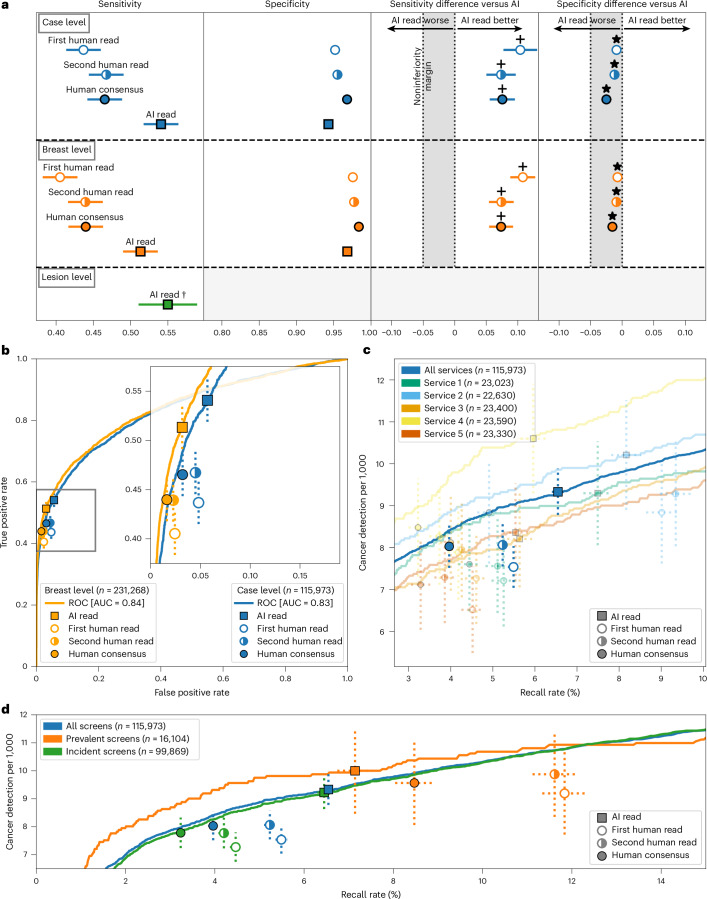
Overview of evaluation results. **a**, Sensitivity and specificity at a case (*n* = 115,973), breast (*n* = 231,268) and lesion (*n* = 681) level. *Noninferior, *P* < 0.001; ^+^superior, *P* < 0.001. A Wald test was used for noninferiority, Obuchowski’s extension of the two-sided McNemar test was used for superiority at the case level and bootstrap-based tests were used at the breast level. Statistical testing was performed using one-sided tests at the 0.025 significance level (after correcting for multiple comparisons). For the primary endpoint of AI against first human read, sensitivity was superior at *P* = 2.8 × 10^−16^, while specificity was noninferior with *P* = 6.1 × 10^−181^. Shaded columns represent the prespecified 5% noninferiority margin. ^†^Historical human readers did not mark lesions digitally; thus, we were only able to plot AI results. Ground-truth lesion ROIs were only available for services 1 and 2, where the case-level sensitivity was 0.61 for comparison. It was not possible to label 5.4% (39/721) of interval or next-round cancers because of missing records. **b**, ROC plot showing breast-level (orange; *n* = 231,268) and case-level (blue; *n* = 115,973) results for the first, second and consensus screening decision against the AI read. **c**, Case-level performance for the five screening services as measured by recall rate and CDR (*n* = 115,973). Lines for each service represent the range of possible AI OPs, with the study-selected threshold marked (squares). Error bars represent the 95% CIs. **d**, CDR against recall rate for all five screening services combined at a case level, split by prevalent screens (women’s first screens) and incident screens (screens where prior imaging exists); *n* = 115,973 total screens, 16,104 prevalent screens and 99,869 incident screens. Error bars represent the 95% CIs. Source data

The AI system achieved superior sensitivity and noninferior specificity to first reader, second reader and consensus decision after arbitration, at a case and breast level (noninferiority margin: 5%, *P* < 0.001 for all; Fig. 2a,b). Across all services, AI cancer detection rate (CDR) was higher versus the first human reader (9.33 per 1,000 women, 95% confidence interval (CI): 8.78, 9.88) versus 7.54 per 1,000 women, 95% CI: 7.04, 8.03), although the AI recall rate was higher than the first reader (6.5%, 95% CI: 6.4, 6.7 versus 5.5%, 95% CI: 5.3, 5.6). Performance was sustained across all five services, despite varying cohorts and clinical screening practices (Fig. 2c). Full results are presented in Supplementary Tables 2 and 3.

**Extended Data Fig. 2:**
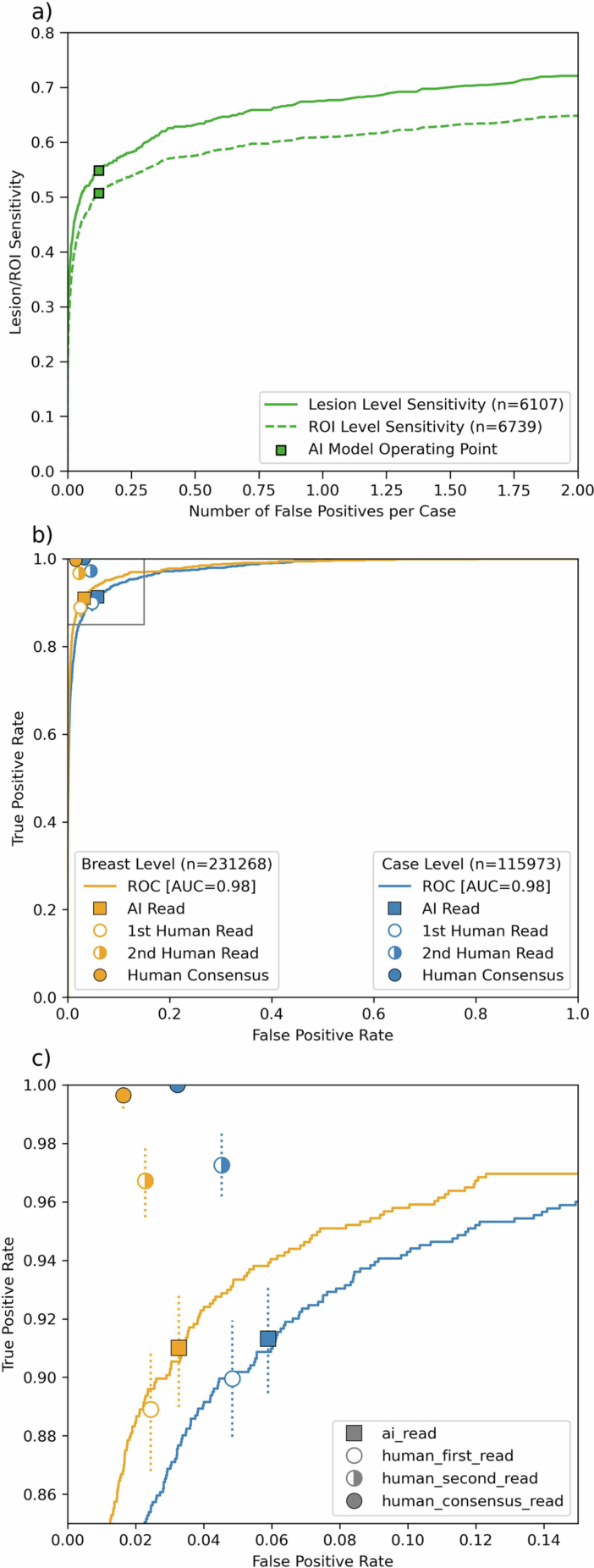
Additional performance charts. **a**) Free-response receiver operating characteristic (FROC) showing both ROI level sensitivity (considering each ROI separately, N = 6,739 ROIs) and lesion level sensitivity (where ROIs are linked to a specific unique lesion, and any hit on that lesion is considered acceptable, N = 6,107 lesions). **b**) Graph showing performance at a case (N = 115,973 participants) and breast (N = 231,268 breasts) level, across all five sites, using a ground truth of screen-detected cancers only (reflecting a common metric in the literature). This metric has a key limitation in that it cannot demonstrate incremental benefit of AI, as it does not include future cancers that developed over the subsequent 3 years. Readers naturally perform well using this metric, due to the gatekeeper effect - that is only cancers that were picked up by the historical double-reader process are considered as cancers, while incremental cancers that the AI system detects are penalized as false positives, rather than rewarded as a true positive. **c**) zoomed region of **b**). Source data

The AI system demonstrated lesion-level sensitivity of 0.550 (95% CI: 0.512, 0.588) (Extended Data Fig. 2a). There was no comparator for human reads, as specialists do not routinely mark suspicious lesions on screening images in a digital form. For the best comparison, case-level sensitivity was 0.61 for the same two sites.

To facilitate comparison to studies that do not consider interval cancers in their ground truth, the AI system achieved case-level sensitivity of 0.913 (95% CI: 0.895, 0.932) and specificity of 0.941 (95% CI: 0.940, 0.942), with an area under the receiver operating characteristic (ROC) curve (AUC) of 0.978 (Extended Data Fig. 2b,c) when considering screen-detected cancers only.

### AI outperformed for first screens

**Extended Data Fig. 3:**
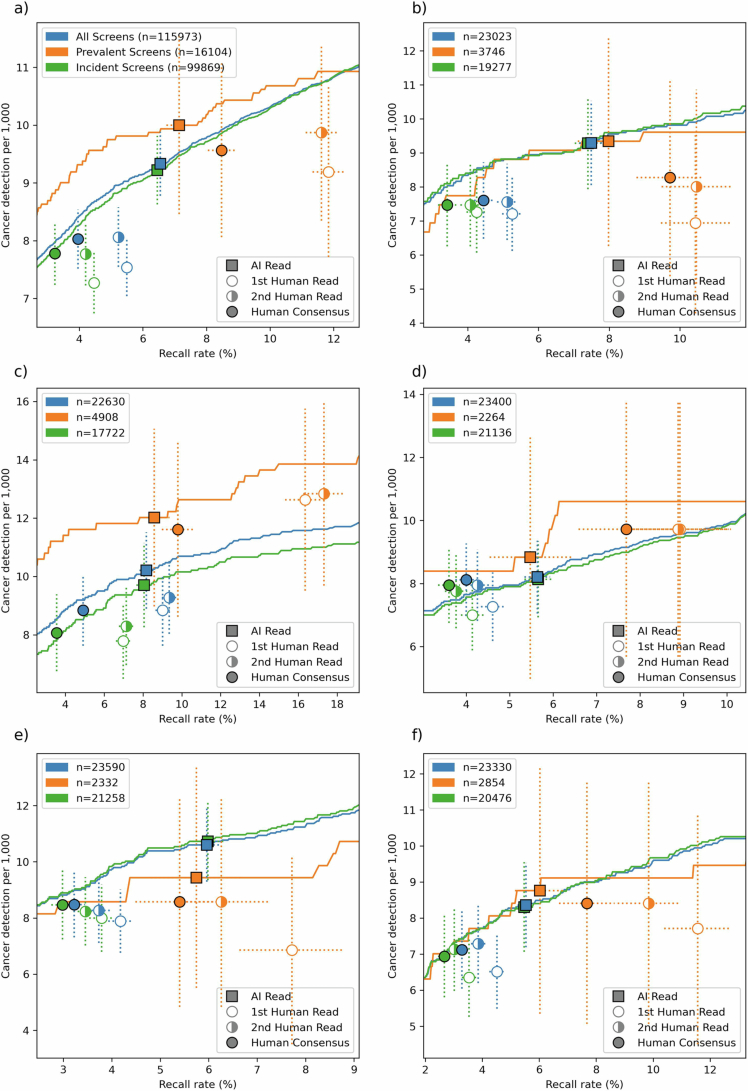
Prevalent and incident screens per screening service, and overall. Plots show cancer detection rate against recall rate curves for prevalent screens (orange), incident screens (green), and both prevalent and incident screens (blue). Individual panels represent: **a**) All screening services (N = 115,973 participants). **b**) Screening service 1 (N = 23,023 participants), **c**) Screening service 2 (N = 22,630 participants), **d**) Screening service 3 (N = 23,400 participants), **e**) Screening service 4 (N = 23,590 participants), **f**) Screening service 5 (N = 23,330 participants). The higher human reader recall rate for prevalent screens (first screens) vs incident screens (subsequent screens) is consistent across all services. AI appears to have potential benefits in the prevalent screen group for all sites. Error bars represent 95% confidence intervals. Source data

The AI reader particularly outperformed when analyzing prevalent screens (women attending for the first time) compared to those who had been screened previously (termed ‘incident’ screens) (Fig. 2d). For these prevalent screens, the AI system achieved the lowest recall rate (7.1%, 95% CI: 6.7, 7.5) versus first human reader (11.8%, 95% CI: 11.3, 12.3) and consensus read (8.5, 95% CI: 8.0, 8.9), while also achieving the highest CDR (AI: 10.0 versus R1: 9.19 per 1,000; difference: 0.81, 95% CI: −0.03, 1.64). For incident cases, the AI achieved the highest CDR but also the highest recall rate. These results were largely consistent at the individual service level (Extended Data Fig. 3a–f).

### Earlier diagnosis through outperformance on interval and next-round cancers

The AI system correctly identified 25.0% (95% CI: 20.4%, 30.0%) of future interval cancer cases, with 88.0% of these localized to the correct breast and 58.1% localized to the precise lesion. For next-round cancers that were only identified at the subsequent asymptomatic screening visit 3 years later, the AI system correctly identified 25.1% (95% CI: 22.1%, 28.1%) of cancer cases, again with 85.7% of cases correctly localized to the relevant breast and 53.1% localized to the precise lesion.

### AI outperformed for women’s first screens and invasive cancers, with no concerning disparities across relevant clinical and sociodemographic subgroups tested in an exploratory analysis

**Fig. 3:**
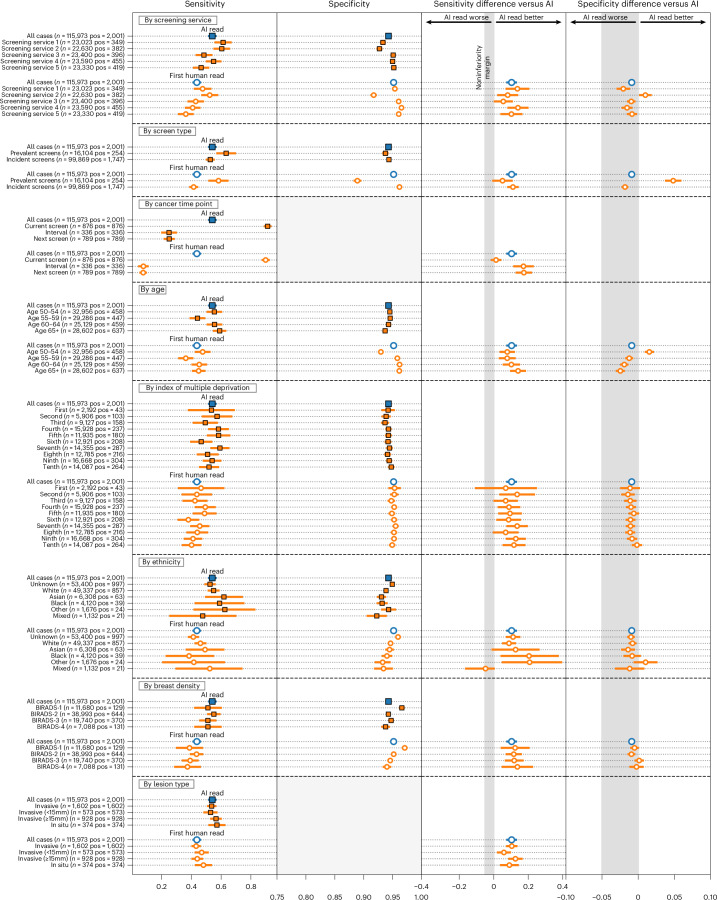
Sensitivity and specificity performance breakdown by subgroups of interest in breast cancer screening. CIs for specificity are too small to visualize for many subgroups. Lesion size analysis considers the largest lesion for each case. Error bars represent the 95% CIs. Source data

We observed no notable differences in performance between the AI and the first human reader across the subgroups tested (Fig. 3). This included age, index of multiple deprivation, ethnicity and breast density. Two subgroups were borderline for sensitivity and failed noninferiority at a prespecified 5% margin, index of multiple deprivation (IMD) decile 1 (AI versus R1 difference: +0.070, 95% CI: −0.103, 0.244; *n* = 2,192) and mixed ethnicity (AI versus R1 difference: −0.048, 95% CI: −0.160, 0.000; *n* = 1,132), although both were groups with few positive cases, limiting the strength of statistical conclusions possible.

AI specificity was within a 5% noninferiority margin for all groups, with the exception of women attending screening for the first time and age group 50–54, where AI specificity was significantly higher. AI generally exceeded first human reader sensitivity, particularly for women over 65 years of age.

**Extended Data Fig. 4:**
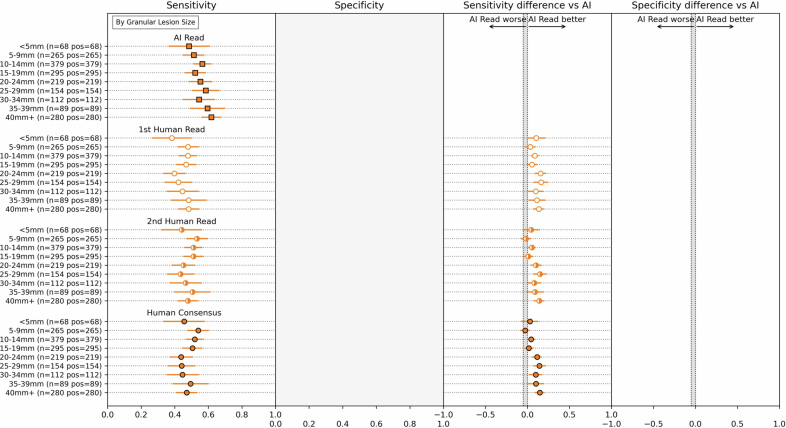
Sensitivity by lesion size. The graph shows sensitivity by lesion size subgroups, against all reads, 1st, 2nd, and consensus reads. Error bars represent 95% confidence intervals. Source data

The distribution of disease detected with AI tended to favor higher-risk over lower-risk cancers. Compared to the first reader, the AI system achieved higher sensitivity for higher-risk cancers (0.55 versus 0.44; difference: 0.109, 95% CI: 0.083, 0.135; superiority *P* < 0.001) and noninferior sensitivity for lower-risk cancers (0.53 versus 0.47; difference: 0.052, 95% CI: −0.021, 0.125; noninferiority *P* = 0.003). For invasive cancers alone, the AI system achieved superior sensitivity compared to first, second and consensus decisions (0.54 versus 0.43, 0.46 and 0.46, respectively, *P* < 0.001 for all). When considering maximum lesion size per case, AI sensitivity performed favorably versus human readers across the range but especially outperformed for 20–30-mm lesions (Extended Data Fig. 4). Because of the low prevalence of cancer, CIs for many subgroups were large despite the large size of the study.

Performance was essentially consistent across Hologic, GE and Siemens devices included in the study. However, within Hologic, we noted that cases imaged using the newer Hologic Selenia Dimensions (*n* = 4,692) demonstrated a distribution shift compared to the older Hologic Lorad Selenia (*n* = 77,840), resulting in a higher recall rate of 10.9% (95% CI: 10.0, 11.8) versus 6.3% (95% CI: 6.1, 6.4). Full results are presented in Supplementary Table 2.

**Extended Data Fig. 5:**
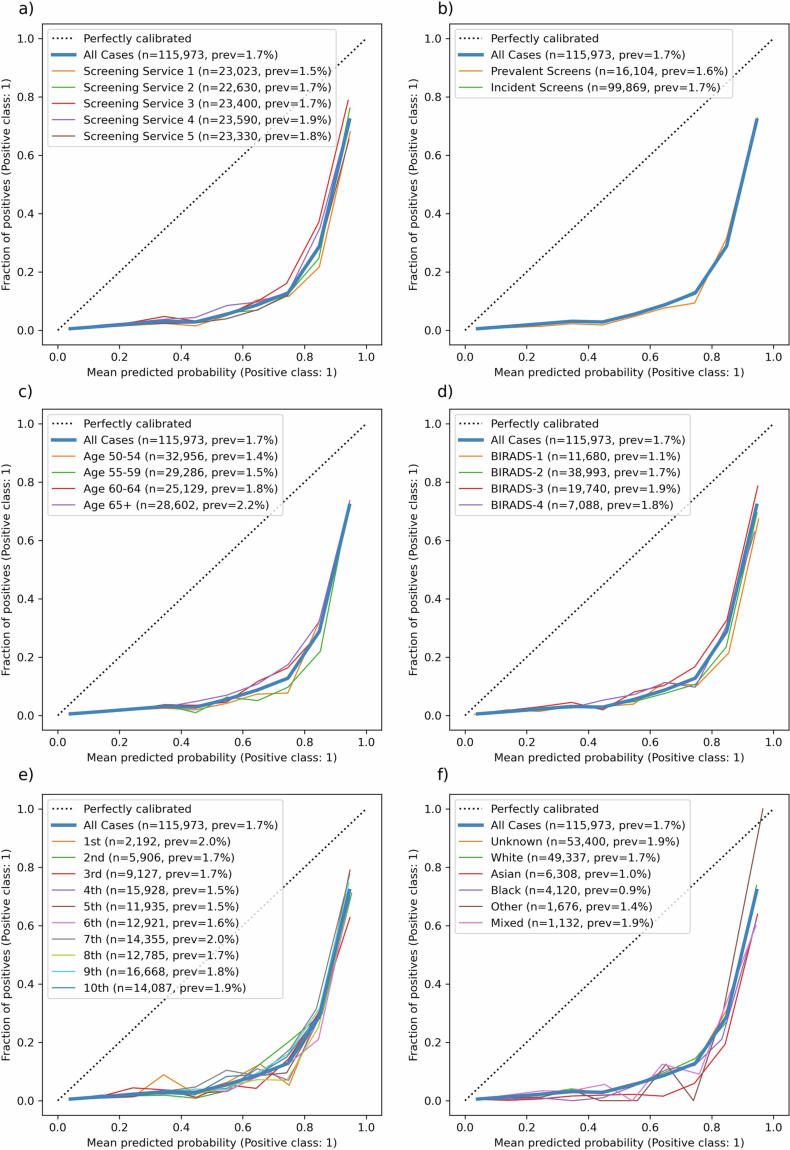
Calibration curves. Calibration curves of the model against different subgroups of interest, alongside overall numbers and percentage prevalence of cancer by: **a**) individual screening services (N = 115,973 participants), **b**) prevalent vs incident screens (N = 115,973 participants), **c**) age group (N = 115,973 participants), **d**) BIRADS score (N = 115,973 participants), **e**) indices of multiple deprivation (N = 115,973 participants), **f**) ethnicity subgroup (N = 115,973 participants). Source data

We assessed model calibration across different subgroups of interest (Extended Data Fig. 5a–f). Overall, there were no concerning disparities between subgroups within the range of the operating points (OPs) selected for this study. We noted that the Asian ethnicity and age 50–59 subgroups were somewhat overcalled compared to the others, although these were most evident at higher model OPs, outside of the range set for each site.

### AI-enabled screening offers increased CDR with reduced reader time required, despite higher arbitration burden

We considered the clinical and operational effect of replacing one of the two historical readers before arbitration. Using AI as a second reader resulted in a 32.1% reduction in total reader time required (195,983 versus 288,616 equivalent reads), while CDR was increased by 17.7% (from 8.7 to 10.2 per 1,000) or 20.2% (from 8.5 to 10.2 per 1,000) for sites that arbitrate all recalls or arbitrate only discordances, respectively.

**Extended Data Fig. 6:**
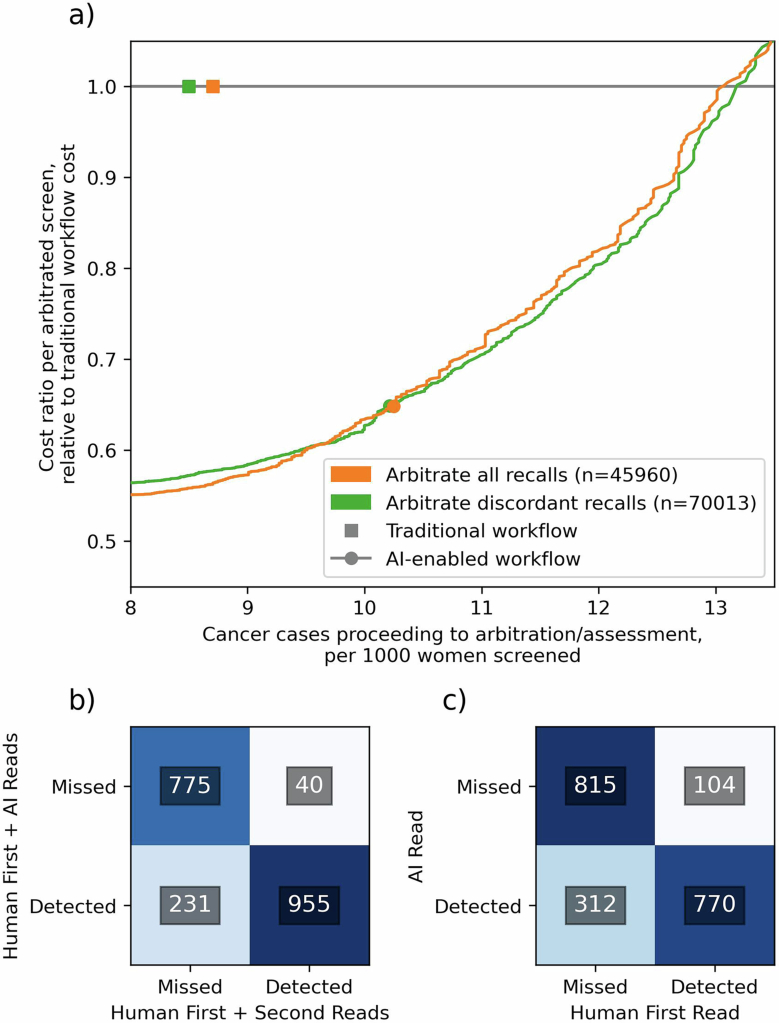
Double-reader workflow costs and confusion matrices for cancer cases. **a**) Demonstration of the effects on overall double-reader workflow cost (expressed in terms of reader time) when altering the model OP, acting as the second reader. We assumed that an arbitration read required 5x the total reader time of a single screening read. Cost is expressed as a ratio vs the human-only traditional workflow cost, and both arbitration strategies (all recalls, N = 45,960 participants, vs discordant only recalls, N = 70,013 participants) are presented in orange and green. We can conclude that an AI-enabled workflow results in a greater proportion of cases proceeding to arbitration, but that reader time in the range of our study’s OP remains reduced vs a human-only workflow. **b**) A confusion matrix from the retrospective study of true cancers detected by either of the first two human readers, or either of the first human + AI system. It shows the uplift in true cancers detected by the AI system, and the overlap in cases between the two paradigms. Of the 40 cancers detected by human double read but missed by the human + AI approach, 35 (88%) were deemed high risk, 3 low, and 2 unknown. Of the 231 cases detected by the human+AI approach, but missed by human double read, 215 (93%) were deemed high risk, 7 low risk, and 9 unknown. This suggests that the distribution of cancers is subtly shifted towards higher risk tumour types. Note that this is ignoring arbitration, and only reflects whether either reader flagged a case as positive. **c**) the same analysis for true cancers detected by the first human read vs the AI system. Source data

Overall, including cases unable to be processed by the AI, which retain their traditional double-read workflow, the number of total human screening reads performed before arbitration was reduced by 46.4% (133,943 versus 249,916 reads), while arbitration reads required were increased by 60.3% (12,408 versus 7,740 reads). Some services use radiographers to perform screening reads but not arbitration reads; thus, estimates of overall workforce cost will vary on the basis of local variation. An example cost sensitivity analysis across the range of model OPs is presented in Extended Data Fig. 6a.

The complementary nature of human and AI reading is highlighted in the detection patterns, with substantial but incomplete overlap between cancers identified by each approach (Extended Data Fig. 6b,c). Of the 40 cancers detected by human double reads but missed by the human + AI approach, 35 (88%) were deemed high risk. Of the 231 cases detected by the human + AI approach but missed by human double reads, 215 (93%) were deemed high risk. This suggests that the distribution of cancers is subtly shifted toward higher-risk tumor types when incorporating AI into the reading workflow.

### Prospective observational deployment

**Fig. 4:**
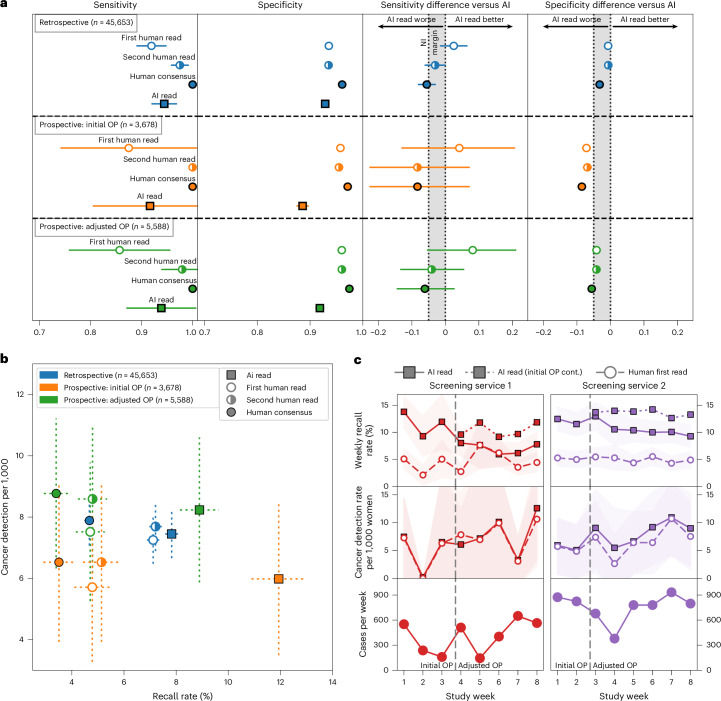
Performance for the prospective observational deployment of the AI system. **a**, The top segment (*n* = 45,653) reflects retrospective study cases from both screening services included for comparison, reanalyzed using the prospective study’s shorter 3-month ground truth. The middle segment (*n* = 3,678) reflects cases read at the initial deployment OP (OP1 until the OP update was performed). The bottom segment (*n* = 5,588) reflects cases read under the adjusted, more specific OP (OP2, latter 4 weeks). **b**, CDR versus recall rate, comparing the AI to human readers, using a within-episode ground truth (that is, for screen-detected cancers only, not including future interval cancers and those detected at the next round), across retrospective (*n* = 45,653), prospective at initial OP (*n* = 3,678) and prospective at adjusted OP (*n* = 5,588). **c**, Recall rate and CDR by week of the study. Dotted lines reflect performance had the Initial OP been continued for the remainder of the study. Error bars and shaded areas reflect the 95% CIs. Source data

Two screening services were included in the prospective deployment covering 12 screening sites across London. Characteristics of the dataset included are summarized in Table 2, with a data flow diagram in Extended Data Fig. 1b. In total, 43 women opted out of participating in the study. While not powered for significance, AI and human reader performance is shown in Fig. 4a,b.

**Table 2:** Datasets generated by the two screening services in the prospective deployment

	Screening service 1	Screening service 2	Both
**All cases**	3,566	7,312	10,878
**Included cases**	3,220	6,046	9,266
**Period 1: initial OP**	1,358	2,320	3,678
**Period 2: adjusted OP**	1,862	3,726	5,588
**Normal**	3,197	5,996	9,193
**Screen-detected cancers** (***n***, **%)**	23 (0.71%)	50 (0.83%)	73 (0.79%)
Age*	59 (55–64)	58 (54–63)	59 (54–64)

*Median, with IQRs in brackets.

Source data

### Adaptive OP selection

We implemented an iterative OP calibration process, setting initial thresholds on the basis of available historical data, followed by monitoring of recall rates, as described fully in the Methods. After approximately 2 weeks, we reviewed the initial metrics available including primarily recall rate. Service 1 had an AI recall rate of 11.3% (human first reader 3.8%), while service 2 had an AI recall rate of 12.3% (human first reader 5.3%). These were both above our target recall rates; thus, we adjusted the OP using the prospectively collected data. The second period of the study had AI recall rates for service 1 of 6.7% (human first reader: 4.7%) and service 2 of 10% (human first reader: 4.7%). There was substantial week-to-week variation in cohort, as shown in Fig. 4c, highlighting the challenges in detecting drift in this type of low-prevalence screening population.

### Prospective deployment maintained accuracy despite a distribution shift

**Extended Data Fig. 7:**
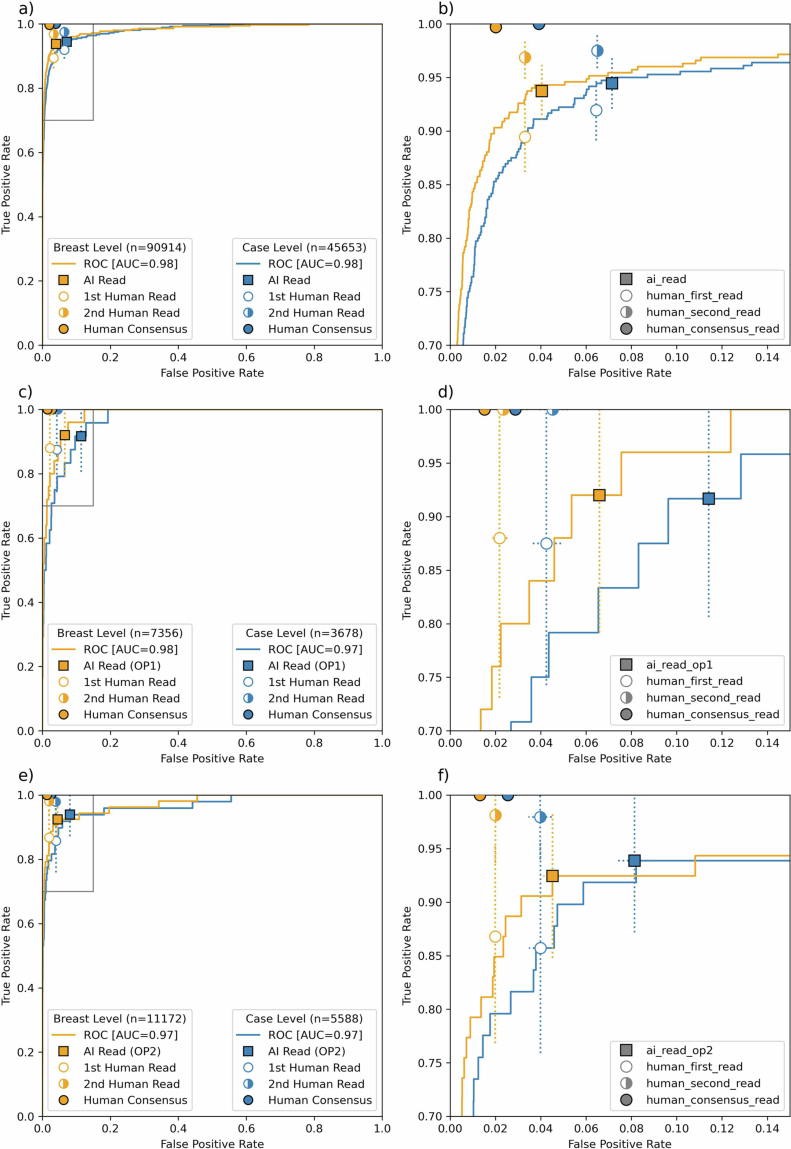
ROC plots for the prospective feasibility study. Ground truth is within-episode (cancer within 3 months), as we do not have longer-term follow up. **a**) shows matched ground truth results from the retrospective study. **c**) is the first period of the study at the initial OP (OP1). **e**) is the second period of the study at the adjusted OP (OP2). **b**), **d**) and **f**) are the zoomed regions of **a**), **c**) and **e**) respectively. Error bars represent 95% confidence intervals. Source data

Across both sites, time from screen to completed AI read was 17.7 min (interquartile range (IQR): 8.5–37.6 min), while time from screen to first human read was 2.08 days (IQR: 1.00–3.81 days). Relative accuracy was maintained in the prospective deployment compared to the retrospective study (breast-level AUC: 0.98 for both; Extended Data Fig. 7a–f and Fig. 4a, top row versus second and third rows) when reanalyzing using a comparable ground-truth definition. When deployed at the screening sites, the initial OP was set to an overly sensitive threshold, resulting in a higher recall rate and associated lower specificity. This highlighted a distribution shift between the original data used to set the OP from 2016 and the newer deployment time period in 2023, with both human and AI being affected (Fig. 4b). Marked variations in cohort can be seen from observing AI and human performance data in week-to-week plots (Fig. 4c), highlighting the challenges in closely monitoring AI accuracy and safety after deployment.

### Updated national guidelines and technical functionality would be required

The National Health Service (NHS) Breast Screening Programme (BSP) mandates a double-read workflow using human readers. In our AI-enabled workflow, an AI system replaces the second reader. However, as the AI cannot process all cases (for example, for cases that do not meet the intended use criteria or because of technical failure), the ability to invoke a human second reader remains necessary. At the time of the study, the National Breast Screening System (NBSS) software lacked functionality to automatically write back AI results and required an ‘assessment reason’ for recalls, which AI does not currently provide. Therefore, to ensure that the BSP is AI ready from a technical perspective, changes will be required to both national program guidelines and the IT system.

### Local variation is currently widespread in screening

Despite elements of national standardization, the BSP allows for site-specific variability in other aspects of the workflow (Supplementary Table 4). This includes some flexibility in reading practices, devices and staffing allocations. This allows local services to adapt workflows to their unique circumstances such as local reading performance goals and staffing levels, which needs to also be considered for AI workflows.

### Migration to digital workflows with strict data standardization will facilitate integration

**Extended Data Fig. 8:**
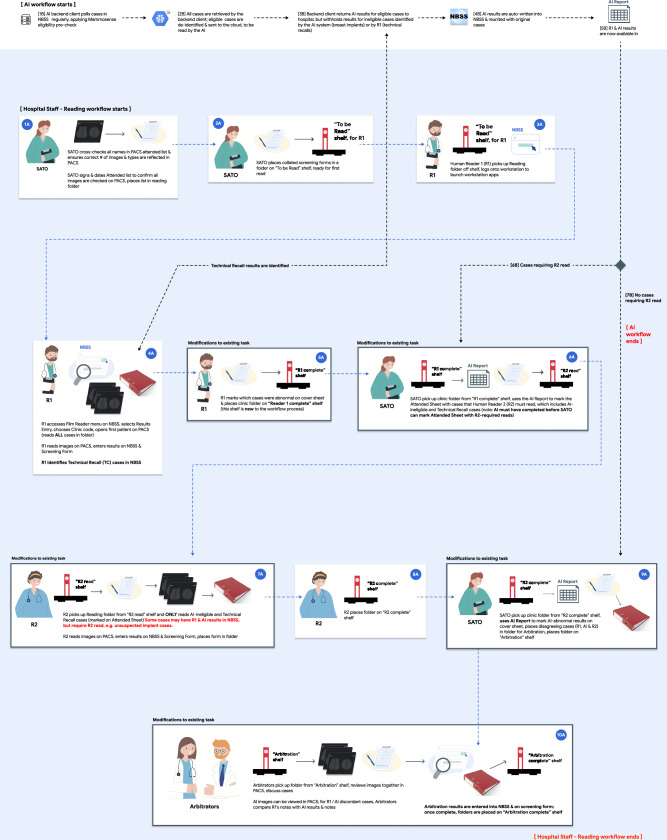
Storyboard showing the way in which an AI breast tool can be implemented into a paper breast screening workflow. Storyboard produced through hands-on workshops, interviews, and group sessions including radiologists, radiographers, screening administration staff, and user experience researchers and designers.

An important factor for the feasibility of AI adoption is workflow digitization and standardization. At the time of research, eight of nine services interviewed relied on paper to drive the workflow (Supplementary Table 4). Readers relied upon client worksheets and physical processes to indicate which reading step was needed. Despite introducing redundancy and complexity, paper documentation was viewed as an important failsafe to check that the correct results were sent. AI cannot interact with physical documents; thus, full digitization would simplify an AI-enabled workflow (Extended Data Fig. 8). Furthermore, full standardization of workflow data collection (for example, including DICOM tags) would also facilitate AI integration. However, achieving this level of digitization will require extensive investment and effort.

Further details about the workflow design process are available in Supplementary Note 1.

## Discussion

Our study demonstrates that AI can match or exceed specialist radiologist performance in breast cancer screening across diverse UK populations and workflows. Notably, the system detected 25% of cancers that would otherwise present as intervals or at next screening, suggesting potential for earlier diagnosis and improved outcomes. The AI system demonstrated higher sensitivity and noninferior specificity compared to the first reader at the case level. Importantly, we did not find systematic trends suggesting harmful bias across subgroups. The AI system particularly excelled in women’s first screens, improving CDR while also dramatically reducing false positives. Prospective deployment at 12 screening sites allowed us to test an iterative approach to OP selection and consider key issues such as AI monitoring for performance and safety. While we retrospectively found that AI can perform as well as or better than human readers, our prospective feasibility results highlight the challenges in implementation.

Our comprehensive evaluation framework included breast-level and lesion-level analyses, alongside detailed subgroup analyses, and provided a rigorous approach to assessing AI performance in breast cancer screening. We found that traditional case-level metrics do not fully convey the nuanced performance of AI systems or the more granular localization information that specialists rely upon for effective human–computer collaboration. We propose that breast-level and, ideally, lesion-level analysis should become the standard metric for comparison to convey actionability of AI results. Our rigorous definition of ground truth based on 39 months of follow-up, including interval and next-round cancers, provides a comprehensive AI comparison to humans, although absolute sensitivity will appear lower than values reported in existing studies as a function of their shorter ground-truth period. Additionally, our careful evaluation of the validity of the intersection over union (IoU) metric contributes to the methodological rigor of this approach.

This study corroborates previous findings that breast AI can operate at least on par with human specialists^2–4,9^. Furthermore, we show that AI systems can significantly outperform human readers in first screens where no prior images are available compared to its noninferior performance in subsequent screens. This insight aligns with UK national targets, which allow for a 2.3-fold higher referral rate in first screens because of the lack of prior imaging for comparison^10^. AI may have a future role in reducing false-positive recalls for this important group of women. Similarly, the AI’s superior performance was particularly evident in detecting invasive cancers, the most clinically consequential subtype affecting outcomes^11^. This is an important positive shift in distribution of disease detected, bringing focus to cases that are most likely to cause future mortality.

The potential for AI systems to cause harm because of preventable blind spots that could go unnoticed is a notable concern, as highlighted by prior experiences with breast computer-aided detection software^12^. The implications of such harm are profound at scale and require careful consideration, including ongoing monitoring to maintain fairness and prevent performance degradation because of data drift. Our research found that accuracy and AI calibration curves were consistent across our primary subgroups of interest. However, achieving real-time fairness monitoring is challenging, as evidenced by our study, where even 2 months of data across two services provided insufficient sample sizes for conclusive fairness analysis. To address this, a multifaceted approach is necessary, incorporating a cascade of fairness metrics, analyzing data at various time resolutions and using rolling windows to ensure adequate sample sizes for robust evaluation. Furthermore, UK national breast screening must enhance its collection of crucial fairness attributes, such as ethnicity, to facilitate the systematic and continuous monitoring of equity in AI-powered breast cancer screening.

Although the present work focuses on validation, the AI system itself was trained on a deliberately broad corpus spanning different geographies, screening sites, vendors and acquisition protocols. During training we benchmarked cohort characteristics against national screening statistics to verify coverage of key demographic and technical subgroups and supplementing the corpus or applying class‑balanced sampling wherever possible. Future releases should formalize these fairness audits by setting minimum thresholds for each protected attribute and expanding the training pool further to actively enrich underrepresented groups and further mitigate bias.

Setting an appropriate OP threshold, which determines where the model’s cancer score triggers a cancer recall decision, is a complex task with technical, clinical workflow and capacity considerations. Our prespecified OP strategy prioritized sensitivity at the expense of specificity, leading to higher CDRs at the expense of higher recall rates. Post hoc analysis suggested that adjusting the AI threshold to match human specificity levels could reduce false positives while maintaining competitive CDRs. It is not safe to assume that the same OP will work for every deployment and careful consideration is required of technical, clinical and population factors that may shift over time^13^. Given the drift observed in our study, with changes in machine type resulting in a doubling of the recall rate, our study emphasizes the need for a phased, iterative approach to AI deployment to ensure that model thresholds are carefully calibrated to the local environment. AI tools will also require continuous monitoring to ensure ongoing AI system safety, effectiveness and fairness for all, supported by new regulatory frameworks including predetermined change control plans. Our study had 8 years of lag between datasets used for tuning and prospective testing. We believe that this lag exposed a number of sources of drift, including device shift (for example, introduction of Selenia Dimensions) and human behavior shift (human readers became more specific, especially at service 2), combined with differences in case mix (prospective cases for the adjusted OP period had a higher prevalence of cancer compared to the initial OP period). We anticipate that moderate drift can be compensated using OP adjustments, while substantial changes in imaging acquisition may require model retraining.

Full workflow digitization and data standardization will enable more safe and efficient deployment. However, at the local level, service variability necessitates workflow flexibility. Early validation of integration solutions with a range of sites will ensure solution generalizability. Successful AI deployment into the UK screening program requires a coordinated effort across multiple stakeholders including local breast screening services, policymakers and technology deliverers for safe and effective implementation. Additionally, ongoing real-time fairness monitoring remains a critical but challenging component of deployment, requiring centralized oversight, careful consideration of statistical thresholds and clearly defined performance proxies to promptly identify and address biases or disparities.

Our study had a number of limitations. Despite the large size of our retrospective cohort, some subgroup sizes remained limited, leading to wide CIs for sensitivity. In particular, for socioeconomic status (IMD), the borderline result in decile 1 was not supported by a broader trend across adjacent deciles, suggesting that this was likely because of statistical variability rather than a systematic performance deficit. The mixed ethnicity subgroup particularly illustrates the challenge of very small subgroups, with only 21 positive cases and nearly complete concordance between human and AI readers, making reliable inference difficult irrespective of statistical methodology. This subgroup analysis is further complicated by inconsistent or incomplete data collection at NHS sites, highlighting the critical importance of accurate demographic data collection to robustly assess subgroup performance. Larger, more diverse datasets will ultimately be needed to assess subgroup fairness with confidence. Using our prespecified clinical noninferiority margin of five percentage points, we estimate that roughly 150–200 screen-detected cancers per subgroup (or approximately 10,000–15,000 examinations) are required to keep the 95% CI half-width within ±5 points and, thus, test equivalence directly. Should a model’s point estimate already exceed human performance by more than this margin, fewer cases may suffice; however, the proposed threshold provides a conservative target for future work.

This study did not assess human–computer interaction to explore the impact of AI on radiologists’ decision making on accuracy and overall workflow efficiency but this is addressed in a companion study^14^. While we were able to estimate reader time burdens, we were not able to assess the effect of AI on downstream costs including assessment clinics, diagnostics and investigations, which would require an interventional study. Future studies exploring these aspects are crucial for understanding the effect of AI on the entire screening system and for conducting robust health economics and outcomes research.

Optimal OP selection and ideal update frequency remain open questions, with a need to determine whether an approach with higher recall and higher CDR, albeit with more false positives and potentially lower user trust, is preferable to an approach with lower recall and higher specificity. Independent, robust platforms to monitor performance in real time will be essential to mitigate potential risks resulting from data drift in deployment. In our calibration analysis, we noted that the between-subgroup calibration was acceptable but the model was not perfectly calibrated to generate a disease probability. Future work should explore whether adding a calibration model could help standardize OP selection across sites and help understand the factors with the greatest effect on distributional differences. Our prospective study would ideally have run for longer than 8 weeks to assess performance metrics changing over time and longer analyses will be required to give confidence about AI stability.

In this study, we endeavored to achieve a sample that was representative of the target screening population to comprehensively assess fairness. However, to achieve the highest-quality negative ground truth, we excluded women without a follow-up negative screen. This may have removed some underserved women from our study sample who were unable to attend regular screening for socioeconomic reasons and reflects the wider challenge of achieving equitable screening uptake in population screening.

While specificity is a conventional metric for evaluating diagnostic accuracy, it may mask clinically important differences that become more apparent when expressed as false-positive rates. Even small reductions in specificity can substantially increase the relative number of individuals without disease undergoing unnecessary further investigations. Lastly, it will be important to understand how increasing user experience with AI systems affects specialists’ ability to make the highest-accuracy decisions and develop strategies for user training to minimize ramp-up time.

In conclusion, this study demonstrates AI’s potential to substantially improve breast cancer screening efficiency and accuracy, particularly for first-time participants. However, successful implementation will require adaptive threshold management, continuous performance monitoring and careful workflow integration to ensure equitable benefit across all populations.

## Methods

### Study design

‘AIMS’ (AI in Mammography Screening) is a multiphase research program to evaluate an AI system for potential deployment within UK national breast cancer screening (Fig. 1a–c). This study comprised two phases: (1) retrospective multicenter evaluation, leading to (2) prospective observational feasibility deployment. A companion virtual clinical trial accompanies this study to assess overall AI-enabled screening system performance^14^. Further information on research design is available in the Nature Portfolio Reporting Summary linked to this article, and in the study protocols, attached in the Supplementary Information.

### Phase 1: retrospective multicenter study

The retrospective multicenter study was performed at five screening services: Cornwall Breast Screening Service, Leicester and Rutland Breast Screening Service, Oxford Breast Imaging Centre, South West London Breast Screening Service (SWLBSS) and West of London Breast Screening Service (WoLBSS). These services are henceforth referred to by randomly assigned identifiers services 1–5. Women aged 50–70 were eligible for study inclusion if they underwent routine breast cancer screening as part of the BSP. Study exclusion criteria were poor diagnostic quality imaging (requiring repeat imaging), women not undergoing routine screening (for example, high/moderate-risk special screening and those with personalized stratified follow-up), presence of breast implants, incomplete or nonstandard acquisitions (for example, imaging beyond the standard four views) and lack of follow-up that precluded ground-truth determination as defined below. The study protocol was approved by the East Midlands Nottingham Research Ethics Committee (22/EM/0038) and BSP Research Advisory Committee (BSPRAC_0093). The study was registered with UK Clinical Study Registry (ISRCTN; 60839016).

### Phase 2: prospective multicenter technical feasibility study

The prospective multicenter technical feasibility study was performed at two screening services: SWLBSS and WoLBSS, comprising 12 screening sites. Services 1 and 2 in the retrospective study map to services 1 and 2 in the prospective study. Women aged 50–70 were included if they underwent routine screening as part of national breast screening during study dates November 27, 2023 to January 19, 2024 (WoLBSS) or December 4, 2023 to February 9, 2024 (SWLBSS). Exclusion criteria matched phase 1, except for a shorter follow-up period for ground truthing, given the prospective nature of the study. The study protocol was approved by the East Midlands Nottingham Research Ethics Committee (22/EM/0198), BSP Research Innovation and Development Advisory Committee (BSPRAC_0093b) and NHS England’s Research Advisory Committee (BSPRAC_0093). The NHS Confidentiality Advisory Group approved the study for an opt-out consent approach under section 251 of NHS Act 2006 (22/CAG/0124). The study was registered with the ISRCTN (88754382).

### Patient and public involvement

A study-specific patient and public involvement group included women across the UK, who provided regular advice and input to protocol design and study execution. Two group members provided representation on our steering committee and joined regular team meetings.

### AI system

**Extended Data Fig. 9:**
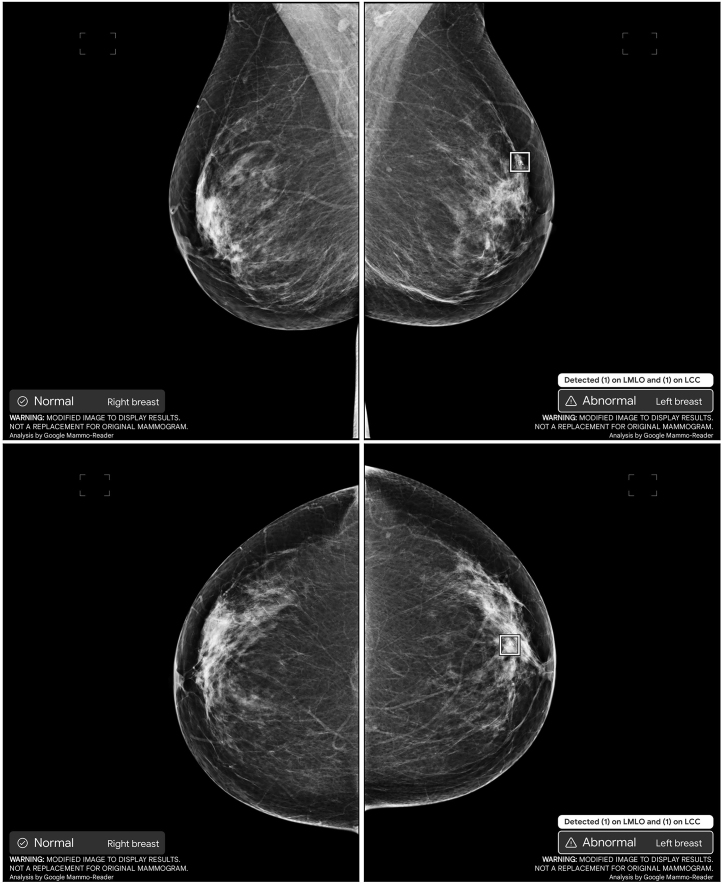
Example output of the AI system. Images represent a correct AI cancer detection for a case that was not recalled by the human readers at this screen, but was screen-detected at the next round, 3 years later. This was a biopsy confirmed invasive G2 cancer, diagnosed 36 months after this screen. Top images are mediolateral oblique (MLO) and bottom images are craniocaudal (CC).

The breast cancer screening AI system (version 1.2, Google) is an updated version of the version 1.0 model described previously^9^. Architectural improvements are described in Supplementary Note 1. The AI system considers four standard full-field digital mammography images and returns a case and breast-level decision alongside suspicious regions of interest (ROIs), thresholded by a preselected OP. An example AI output is presented in Extended Data Fig. 9. The AI system used prespecified exclusion criteria including mammogram size below 3,000 × 2,000 pixels, missing views, additional views beyond the standard screening four views, cases marked as a technical recall by reader 1 and cases with breast implants.

### Retrospective standalone diagnostic performance evaluation

We randomly selected 25,000 women per screening service aged 50–70 who underwent routine screening during 2016 and who had a subsequent screening attendance at 24–39 months or a documented cancer within 39 months. We allowed up to 39 months to accommodate slippage in the intended 36-month screening interval used in the UK. Two services without sufficient cases in 2016 were extended chronologically backward into 2015. Women under 67 years of age without follow-up were replaced with women who did, matched by age and whether it was a first or subsequent screen. Any women whose images were previously used to train or test the AI system were replaced with other women. We found that interval cancer follow-up data can be incomplete in local service databases; thus, we added a random selection of known interval cancers to bring each service to their reported interval cancer rate, as obtained from the national Screening History Information Management (SHIM) system. Where necessary, the selection year range was extended to 2011–2018 to ensure that adequate interval cancers could be included. Data were collected from each screening service using the OPTIMAM mammography image database infrastructure^15^. Full curation methodology is presented in Supplementary Note 1.

### Derived data used for subgroup analysis

Breast density was calculated for mammograms acquired using Hologic devices using validated software developed by Royal Surrey^16^. Indices of multiple deprivation^17^ were calculated from postcode data before deidentification. Ethnicity data were obtained from routinely collected breast screening data, where available. Lesion size information was obtained from surgery and assessment records, with the largest lesion per case selected for cases with multiple lesions. We defined ‘higher risk’ as all invasive cancers and high-grade in situ cancers, while ‘lower risk’ was defined as low/intermediate-grade in situ cancers.

### OP selection methodology

A service-specific approach to OP selection was designed to account for local differences including clinical workflows, screening ethos, population demographics and types of mammography systems. We used chronologically separate tuning sets with no overlap of women with any other test/tune dataset, collected before the study commencement, to select an OP for each site that maximized sensitivity within clinical and operational tolerance without modifying the actual weights of the underlying model. Full selection methodology is described in Supplementary Note 1.

### Prospective feasibility study

Integration of the AI system was observational, not interventional, and did not influence mammogram assessments or recall rates. Therefore, the results presented in this study reflect what might happen if the AI system was implemented into routine screening practice. The AI system was hosted in a secure cloud environment. Integrations with local site NBSS and PACS systems were achieved through a locally hosted relay (SmartBox, Royal Surrey), which scanned NBSS for the presence of new clinic lists and then retrieved and pseudonymized associated screening mammograms and clinical data, before passing it to the AI system for processing.

### OP determination and continuous monitoring

Changes in characteristics of clinical data on which AI classifications are made are inevitable over time because of factors such as changing clinical practices, populations, equipment and postprocessing of images^18^. This ‘distribution shift’ is a real-world challenge for AI deployment^13^. To address this, we developed a process to set the initial OP and then monitor subsequently.

First, we set an initial OP using historical data from the retrospective study. We reviewed performance at both services after 2 weeks. Given that follow-up outcomes were not yet available, we used recall rate as a proxy metric. We targeted a recall rate such that AI did not exceed first reader by >2 absolute percentage points and the predicted arbitration rate remained <2.5 times the human-only arbitration rate. We updated the system OP and began a new period of monitoring.

### Statistics and reproducibility

For the retrospective study, our primary endpoint was noninferiority (5% absolute margin) of the AI system for cancer detection sensitivity (true positives (TP)/TP + false negatives (FN)) and specificity (true negatives (TN)/TN + false positives (FP)) at the case level, compared to first reader decisions, measured against a 39-month ground truth, as defined below. The 5% margin was agreed and prespecified by the Trial Management Group before study initiation and is stringent compared to historical breast screening literature^19–22^. We prespecified the first reader for our primary comparison, as this compares the AI to a typical human reader most fairly. Statistical testing was performed using one-sided tests at the 0.025 significance level (after correcting for multiple comparisons using the Holm–Bonferroni method). CIs on the difference were Wald intervals^23^ and a Wald test was used for noninferiority^24^. Both used the Obuchowski variance estimate^25^. If noninferiority was shown, a one-tailed superiority test was planned to follow without loss of power or requirement for multiple testing^26,27^. Superiority comparisons were conducted using Obuchowski’s extension of the two-sided McNemar test for clustered data. Clusters were defined to group screens read by the same reader, with a ceiling to the number of reads included in each cluster to avoid deidentifying high-volume individual readers. The data met the requirements of the paired binary tests used (Wald and McNemar). Data collection and analysis were not performed blind to the conditions of the experiments.

Secondary endpoints included noninferiority analyses against second and consensus reads and breast-level noninferiority analyses against all readers for cancer detection sensitivity and specificity at the case level. The analyses followed the same methods as in the primary endpoints. CDR, recall rate, positive predictive value (PPV) and negative predictive value (NPV) for AI models and all readers were also reported. Exploratory analyses included lesion-level analyses (localization free-response ROC (FROC)) and subgroup analyses (sensitivity, specificity and AUC) for fairness. Detailed subgroups can be found in Fig. 3. Multiplicity adjustment for secondary and exploratory endpoints was not performed and there was no defined level of significance for any associated *P* values. Case-level CIs for sensitivity, specificity, CDR and recall rate were calculated by Wald CIs where there were at least 50 samples. Case-level CIs for PPV and NPV and groups with fewer than 50 samples were calculated by bootstrapping. Breast-level and lesion-level CIs were calculated by bootstrapping at the case level. Bootstrap-based tests were used to assess noninferiority and superiority at the breast level. All bootstrapping was performed with 10,000 iterations.

Lesion information was annotated by screening staff at services 1 and 2 using a digital labeling tool (RiViewer, Royal Surrey), assisted by the full patient record including diagnostic imaging and biopsy results. We used IoU > 0.1 as our automated metric to determine whether an ROI appropriately captured the ground-truth lesion, following previous work^9^. A specialist breast radiologist (R.S.) reviewed all IoUs between 0 and 0.30 to ensure robustness of this automated metric and determine whether ROIs would allow successful arbitration and assessment. Full details are provided in Supplementary Note 1. Final clinical decisions were used in lesion-specific analyses. Localization FROC analysis was performed for unique lesions, requiring correct identification in either or both views.

When assessing a breast at the lesion level, for unifocal cancers, we required the lesion to be correctly localized in at least one view to register a true positive. For multifocal breasts with at least one ROI with an IoU > 0, we reviewed each case clinically to determine whether the AI output constituted a lesion-level hit for each breast.

The study’s primary endpoint was powered at a site level with a target power of at least 80%. This required approximately 25,000 women per site, assuming a population prevalence of 200 cancer cases per site.

The study used a stratified random sampling approach tailored to each screening service’s characteristics. For services 1, 2 and 4, an initial random sample of 25,000 women was selected from 2016 screening episodes. Services 3 and 5, with smaller annual volumes below 30,000, required random sampling from the combined 2015–2016 period to achieve the target sample size.

The study’s statistical framework incorporated several key distributional assumptions. The expected interval cancer rate was determined from each service’s SHIM system data for the relevant study period, with a default rate of 3.0 per 1,000 women applied when service-specific data were unavailable, following established literature. When initial sampling yielded insufficient interval cancers relative to expected rates, additional cases were randomly selected from an expanded timeframe spanning 2011–2018 to ensure representative CDRs. Our analysis assumed a standard 3-year follow-up period would adequately capture screening outcomes, with a 39-month allowance to accommodate typical scheduling variations.

Data exclusion criteria were systematically applied across all services to ensure data quality and validity. All sites excluded women with invalid or missing dates, unrecorded ages and episodes outside the age range of 50–70 years or nonscreening episodes. Women younger than 68 years without confirmatory follow-up mammograms were replaced with women who did have a follow-up mammogram from a wider year range (2011–2018), matched by episode outcome, screening sequence and age. Women aged 68 years and above were exempted from follow-up requirements, reflecting their transition out of routine screening eligibility. For services 1 and 2, women whose images were previously used for AI training or AI OP optimization were also replaced to prevent contamination.

For the prospective study, our primary objectives were to demonstrate successful technical integration and assess automated eligibility checks. Women aged 50–70 undergoing routine breast screening at an eligible screening service were enrolled consecutively, following the study’s automated inclusion and exclusion criteria. Through the study’s opt-out consent design, the data distribution was intended to reflect the local center’s routine screening population as closely as possible. High-risk screening, incomplete screening, women with implants, technical recalls, studies with invalid DICOM files and studies with missing or additional views were excluded automatically. Times to first human read and AI read were measured using timestamps from NBSS and the AI system and summarized with medians and IQRs. Although the study was powered as a feasibility study and, thus, was not intended to demonstrate accuracy, measures of recall rate (TP + FP/all eligible cases), CDR (TP/all eligible cases, per 1,000 women), sensitivity (TP/(TP + FN)) and specificity (TN/(TN + FP)) were performed. We planned to run the study for a 4–8-week period, where we estimated that we would screen approximately 10,000 women observationally. All cases were followed for 3 months after their visit to ascertain the ground truth. Given the prospective nature of the study, it was not feasible to replicate the longer 39-month ground truth of the retrospective study.

### Clinical workflow AI integration

To understand the implications and nuances of integrating AI into a typical NHS breast cancer screening workflow, we conducted workflow mappings, workflow codesign and workshops with two services and interviews with seven radiologists from other NHS services. These exercises were designed to surface key workflow implementation challenges related to anticipated changes.

### Effect on clinical workflow and cancer detection outcomes

**Extended Data Fig. 10:**
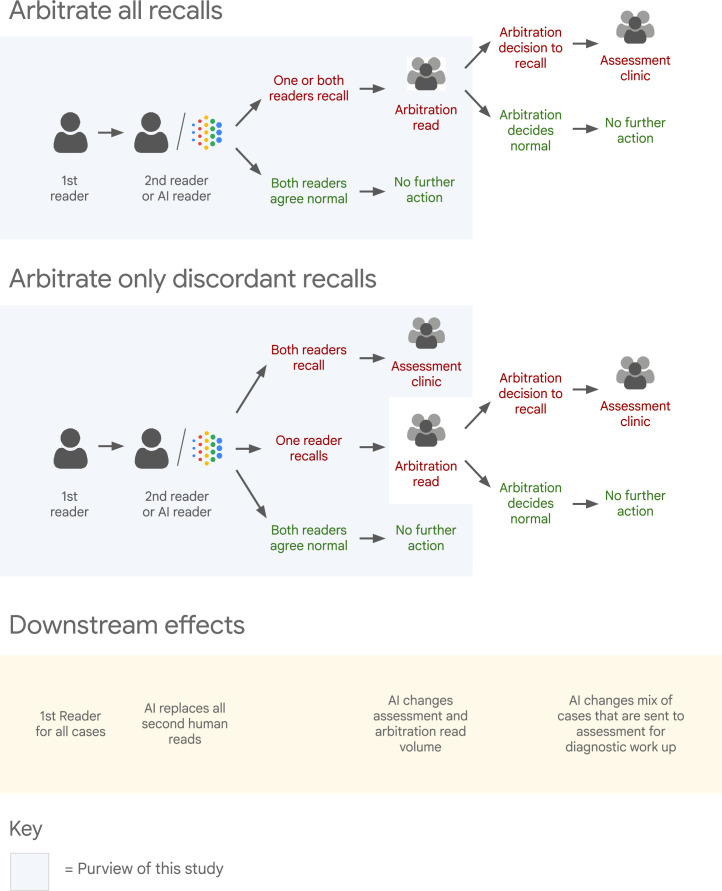
Illustration of the two different arbitration workflows and how introducing AI as a second reader might affect them. The blue regions show the purview of this study, as we do not yet know how readers will respond to the AI in arbitration clinics. In this study, we are able to express screening outcomes in terms of clinical workload (for example change in arbitration and assessment clinic volumes) and cancer detection rate to the point of assessment or arbitration.

We estimated the downstream implications of introducing AI as the second reader, including arbitration and assessment clinic load. Given the retrospective nature of the primary study, we could only model the number of cancers going to arbitration and assessment for both human and AI-enabled arms and estimate the associated human time required. Extended Data Fig. 10 shows both arbitration methods in detail, alongside the boundaries of what was measurable in this study. Derived from previous work, we assumed the overall time cost of arbitration was equivalent to five single reads (average of 132 s for an arbitration read versus 25 s for a single radiologist read)^28^. The prospective observational study was not powered or intended to make conclusions about effects on workflows and outcomes.

### Ethics and inclusion statement

All partners drove the research design to ensure that it was locally relevant. Roles and responsibilities were agreed between collaborators at the outset of the project and researchers at each collaborating site undertook lead roles. The study was approved by local and national ethics review committees as described. There was no risk to participants or researchers from this research.

### Reporting summary

Further information on research design is available in the Nature Portfolio Reporting Summary linked to this article.

## Supplementary information

Supplementary Information Supplementary Note 1 and Study protocol documents.

Reporting Summary

Supplementary Tables Supplementary Tables 1–4.

## Source data

Source Data Tables 1 and 2, Figs. 2–4 and Extended Data Figs. 2–7 Statistical source data.

## Data Availability

The images and clinical data used in this publication are from the OPTIMAM imaging database and are not publicly available because of restrictions imposed by OPTIMAM ethical approval. Instead, the images and data can be accessed after a formal data access request and review by a Data Access Committee and implementation of a Data Sharing Agreement (DSA). Applications for access to the data can be made online (https://medphys.royalsurrey.nhs.uk/omidb/getting-access/). The application, review and agreement process can take anywhere from 2 to 12 weeks depending on the applicant’s desire to customize the template DSA. All other data supporting the findings of this study are available from the corresponding authors on reasonable request. Source data are provided with this paper.

## Code availability

Statistical analysis and plotting was performed using open-source and publicly available Python packages (numpy version 2.3.3, pandas version 2.3.2, scipy version 1.16.1, sklearn version 1.7.1 and matplotlib version 3.9.1), using the methodology outlined in the Methods. All code was executed in Python (version 2.7.18). The AI system (version 1.2, Google) was evaluated but not developed in this study. Code for generating the primary figures is available from GitHub (https://github.com/Google-Health/google-health/tree/master/breast_cancer_aims). The code used for training the models has a large number of dependencies on internal tooling, infrastructure and hardware and its release is, therefore, not feasible. However, implementation details are described in sufficient detail in Supplementary Note 1 to support replication with nonproprietary libraries. The AI system is not currently available for external use but interested parties should contact the corresponding authors to be notified about external availability.
