# Supplementary material for: Diagnostic accuracy, fairness and clinical implementation of AI for breast cancer screening: results of multicenter retrospective and prospective technical feasibility studies
Source: Nat Cancer. 2026 Mar 10;7(3):494–506. doi: 10.1038/s43018-026-01127-0 (PMC13035471; doi:10.1038/s43018-026-01127-0)
Supplement: Supplementary file 1 — Supplementary Note 1 and Study protocol documents. [file 43018_2026_1127_MOESM1_ESM.pdf]

nature cancer

Supplementary information https://doi.org/10.1038/s43018-026-01127-0

Diagnostic accuracy, fairness and clinical implementation of AI for breast cancer screening: results of multicenter retrospective and prospective technical feasibility studies

In the format provided by the authors and unedited

Supplementary Note 1

Model architecture

The AI system used in this evaluation was created by Google (v1.2, Google LLC), and is an updated version of the v1.0 model described previously [1].

The previous three-model ensemble architecture was subsequently improved to combine the different approaches in a more intuitive way. Instead of three separate models performing the tasks in parallel, we unified their functionality into a new hybrid architecture:

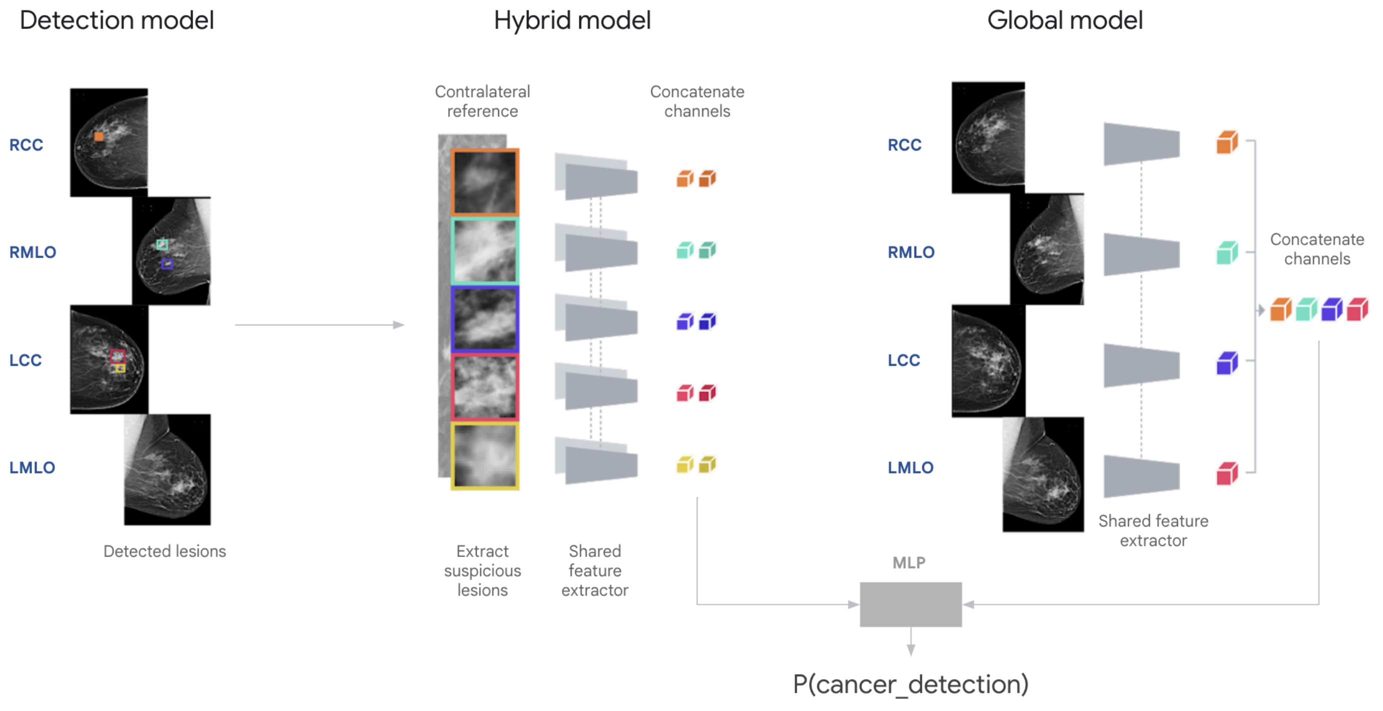

Supplementary Figure 1: Hybrid architecture employed for the study.

As displayed in Supplementary Figure 1, the updated model has the following components:

- **Global model:** this model is similar to the v1.0 case model, designed to take in 4 mammogram views as input and produce a case-level cancer score. But instead of directly using the case-level scores, we leverage its intermediate feature embeddings from the last layer to provide global view information for the hybrid model introduced below.
- **Detection model:** this model is similar to the stage 1 of the v1.0 lesion model which aims to detect bounding boxes of the lesions from each of the views.
- **Hybrid model:** this model is similar to the stage 2 of the v1.0 lesion model, designed to predict lesion-level cancer scores for the regions detected by the detection model. Instead of just

leveraging the zoomed-in image patches, this new hybrid model also takes the global information from the case model as an auxiliary input when predicting the scores.

This new structure enables a joint inference with both the high resolution image information from detected image patches and the global information from all 4 views.

In addition to the above model structural changes, we shifted the focus from case-level cancer prediction to lesion-level cancer prediction for the final output. This allows more accurately performed ROI-level scoring which reduces false positive ROIs and facilitates better human interpretation of the AI outputs. The final case-level prediction is computed by taking the maximum score of the detected boxes.

We also employed the following changes to further improve the model:

- While the overall datasets and labels are identical to v1.0 model, we increased the negative cases by 3x in the training data by relocating 50k cancer negative patients from the tune set to the train set for the OPTIMAM dataset (original training split can be found in Extended Data Figure 9 in McKinney 2020). The larger quantity of cancer negative examples in training helps the model to reduce false positives and the remaining 12,866 patients in the tune set still provides sufficient signal for model development.
- Switching to more advanced backbones:
  - **Global model:** we upgraded from ResNet-50 to ResNet-BiT [2].
  - **Detection model:** we upgraded from RetinaNet to EfficientNet-D7 [3].
  - **Hybrid model:** we upgraded from MobileNetV2 to ResNet-BiT [2].

Image preprocessing and augmentation remained the same as the v1.0 model.

The OP of the model was selected for each site using a separate tuning set collected from women screened at the respective site before 2015 to ensure a temporal separation. Full OP selection methodology is described in Supplementary Materials. There was no overlap of women included in tuning and test sets. OP selection was fixed and agreed by the authors for each site before beginning any test set analysis.

Data curation methodology

We followed guidance from the UK's National Screening Committee [4], which stated:

"Large test sets … should use external validation in either consecutive or randomly selected mammograms; these test sets should also be generalisable to the UK screening population…. and allow long-term symptomatic follow-up of women with negative screening results during the screening interval (i.e., 3 years)."

Therefore, our planned selection process for this study was to select a random selection of women from 2016. This would permit 3 years follow up in 2019, before COVID-19 disrupted screening practices in 2020. Women aged 67 without 3 years' follow-up (39 months, to allow 3 months for slippage of the ideal 3 year interval) were replaced with women with 3 year follow-up examination, matched by age and ethnicity.

Women aged 68+ were permitted to have no follow up screen, as they would not typically be invited back as part of national screening at this age.

Challenges related to data selection

We wished to achieve a representative sample from each site, importantly including interval cancers (the cancers that would ideally have been detected sooner).

Not all interval cancers had been recorded by the screening site, onto NBSS, and so for some services we fell short of the required number. The expected interval cancer rate was determined for each screening service from the national Screening History Information Management system (SHIM) for the relevant time period of the study, if provided by the service. If it was not available, then 3.0 per 1,000 women was used based upon previous literature [5]. Additionally, not all women had returned for their subsequent mammograms, meaning that they did not have a robust negative ground truth determination.

Case Selection – Services 1 and 2

Prior to selection all episodes were categorised as either screen-detected cancer, prior to screen-detected cancer, prior to interval cancer, normal with subsequent normal follow-up mammogram, normal without subsequent follow-up mammogram.

The episodes were then filtered to ages 50-70 years and screening episodes. Any women with invalid or missing dates, or with no age recorded were excluded. The AI developer had used 10,000 cases from each service in order to select the OP which is optimal for the recall rate for the local site. The vendor advised that they would do this prior to clinical implementation at any NHS Breast Screening Programme services, and therefore this mimicked the clinical situation. Therefore, all episodes for these women from any year were removed prior to selection of the study dataset.

The initial sample of 25,000 women per service was randomly selected from 2016. At both services, the interval cancers in NBSS were lower than reported to SHIM. Therefore, the additional prior to interval cancers to reach the numbers reported by SHIM were selected from a wider year range 2011-2018.

There was a proportion of women with normal mammograms, but no subsequent normal mammogram to confirm the negative ground truth. These included women older than 67 years who do not have a subsequent screening episode because they have 'aged out' of the screening age range, and women who are younger than 68 years but did not return for a mammogram for a variety of reasons. To ensure a high quality ground truth, for women younger than 68 years these women were replaced with women who did have a follow-up mammogram from a wider year range 2011-2018. The women were matched by episode outcome, whether it was first of subsequent mammogram and by age (for Service 1 within +/- 1 year and for Service 2 within +/- 3 years). It was not possible to match on ethnicity.

For Service 2, there was also a proportion of women who had been used to train the AI tool. These were also replaced with women who had not been used to train the AI tool.

Case selection – Services 3 to 5

Prior to selection all episodes were categorised as either screen-detected cancer, prior to screen-detected cancer, prior to interval cancer, normal with follow-up, normal without follow-up.

The episodes were then filtered to ages 50-70 years and screening episodes. Any women with invalid or missing dates, or with no age recorded were excluded. Service 4 screen >30,000 women per year, and therefore the initial dataset of 25,000 women was selected from 2016. Services 3 and 5 screen <30,000 women per year, and therefore the initial dataset of 25,000 was randomly selected from 2015 and 2016. OP selection and matching was performed as per Services 1 and 2 above.

Validating IoU as an AI localisation accuracy metric

Services 1 and 2 performed additional lesion-level annotations for each cancer for this study, yielding 682 cancer positive cases for analysis. We performed a manual review to assess the effectiveness of IoU as a metric for effective localisation. Out of 8 cases between IoU of 0-0.1, one case represented a true positive localisation of a cancer despite the small overlap, while the other 7 were judged a miss. Of 14 cases were between IoU 0.1-0.2, 12 were considered true positives and 2 were judged localisation misses. All 29 cases between IoU 0.2 - 0.3 were considered true positive localisations. Multifocal cases are also clinically reviewed to provide a clinical determination as to whether the lesions that were hit constituted a successful breast level hit.

A sample of results are displayed in Supplementary Figure 2, demonstrating IoU metrics alongside clinical review decisions. A broader selection of cases and their IoUs is shown on the following page in Supplementary Figure 3.

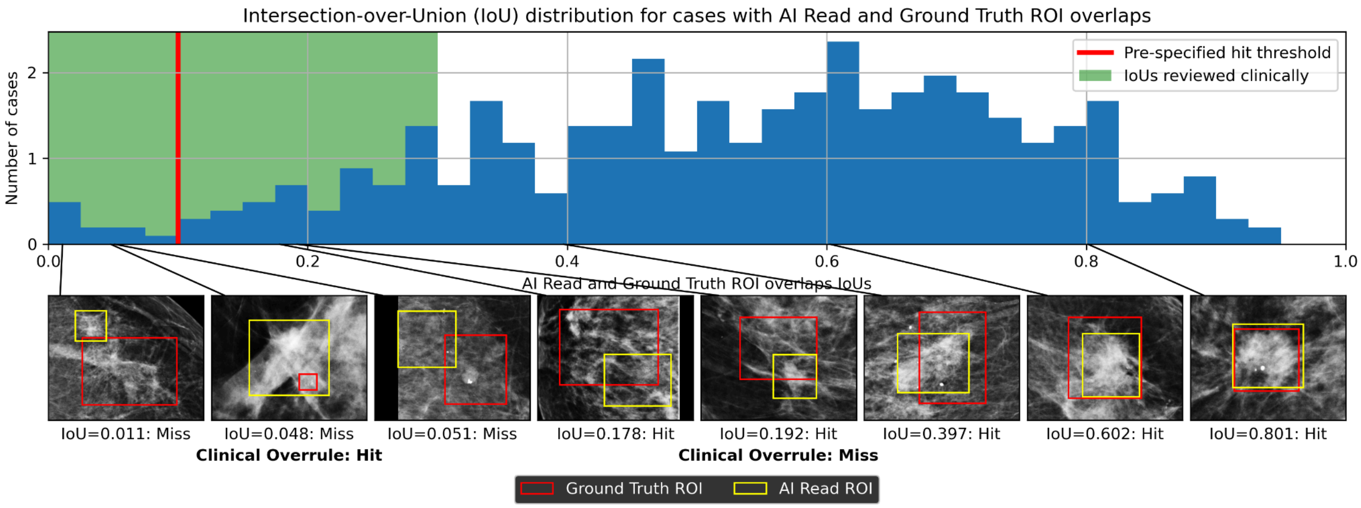

Supplementary Figure 2: Distribution of IoU for all cancer cases. Top: a histogram of bucketed IoU scores. Bottom: example cases sampled across the distribution. We had pre-selected IoU 0.1 and above as our "hit" threshold, following from previous work. To ensure correctness, all cases below 0.3 were reviewed by a breast radiologist (region highlighted in green). Example cases that were overruled after clinical review are shown in bold with the label "Clinical Overrule".

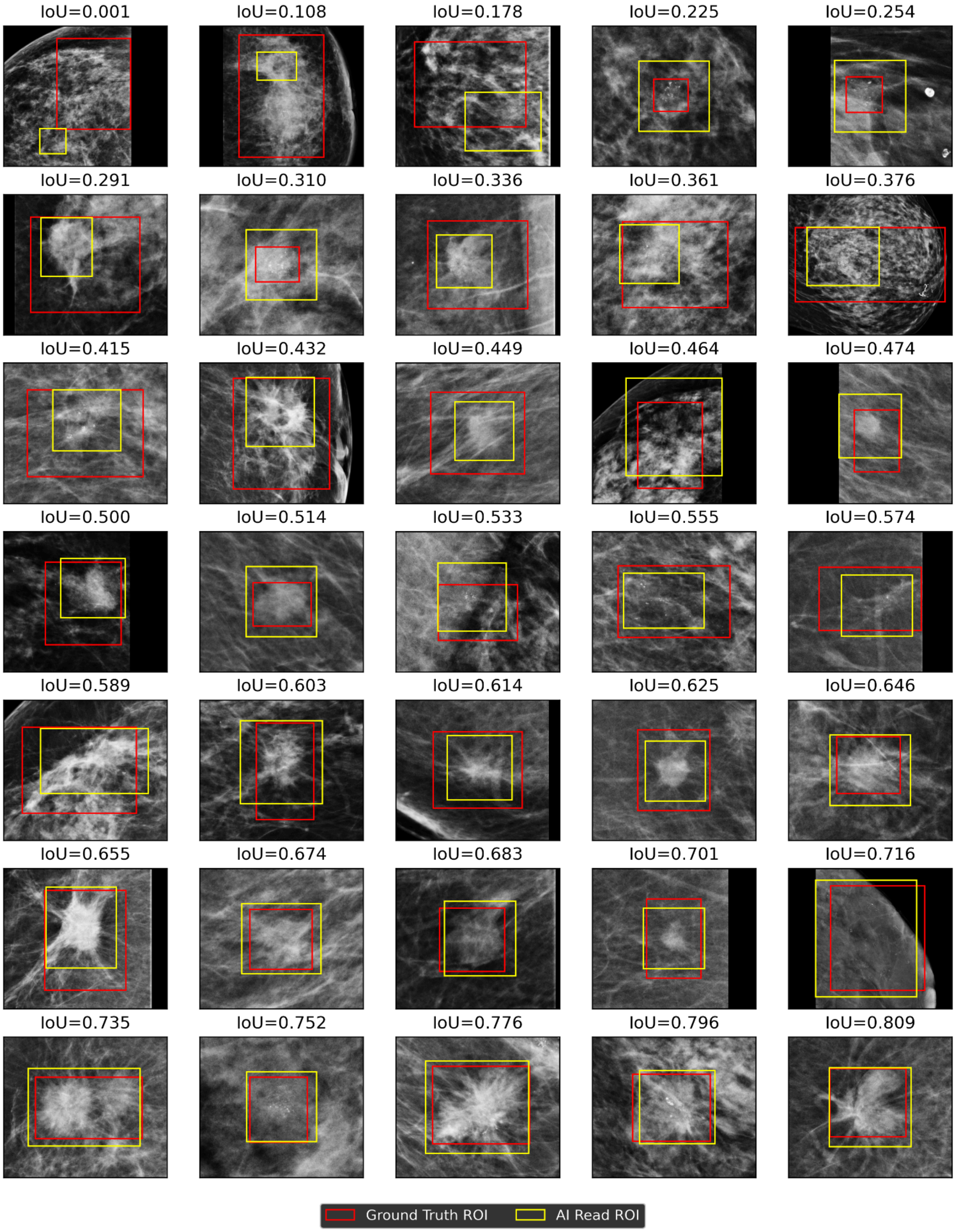

Supplementary Figure 3 - A random selection of cancer cases that the AI recalled as cancer, with AI regions of interest overlaid (yellow) compared to human labelled ground truth (red). IoU is shown for each case. False positive ROIs (e.g. with no corresponding expert marked cancer) would result in an IoU of 0 and are not included here.

Operating point selection methodology

The model outputs a case-level score between 0 and 1, where 0 is most likely to be normal, and 1 is most likely to be suspicious for cancer. An operating point (OP) is the threshold at which the model classifies cases into normal and abnormal. OPs were selected for each site using a tune set collected from 2015 and earlier, excluding any unique women who were in the main test sets that were sampled from 2016 and later, to aim for chronological separation between tune and test sets. OPs were agreed and recorded by the authors before any test set analysis was initiated.

OP selection was performed according to a set of predefined “satisficing” criteria:

1. Maximise chances to meet study endpoints:
    a. Minimum 95% power for model vs. first reader non-inferiority with a 5% margin for sensitivity and specificity
    b. Minimum 95% power for model vs. second reader reader non-inferiority with 5% margin for sensitivity and specificity
    c. Minimum 95% power for model vs. consensus reader non-inferiority with 5% margin for sensitivity and specificity
2. Should not increase the number of arbitration cases more than 2.5 times.
3. Minimum 90% power for sensitivity of first+model superior to first+second human readers, on cases sent to arbitration/assessment (dependent on site policy for arbitrate only discordant or all recalls).

When the above criteria can not be met simultaneously, we relax 1.b and 1.c to minimum 80% power.

For each candidate OP, we perform patient-level bootstrapping to compute power related criteria above. To make the selection more robust to unseen data, we added a small amount of noise (uniform sampling from (-0.025, 0.025)) to the model’s case-level predictions for each bootstrap sample. The number of arbitration cases are computed based on simulated first reader + AI, and first reader + second reader scenarios given the site's arbitration strategies.
Each criteria item generated an OP range for each site. The final OP was then selected by research team consensus from the overlapping ranges to achieve the optimal OP for the study, mostly favouring lower OP for higher sensitivity for better chance of detecting more cancers.

This approach resulted in OP thresholds for Services 1 - 5 of 0.312, 0.38, 0.38, 0.4, 0.5 respectively.

Survey of screening service practices

As part of our work to understand the landscape of screening services in England and Wales, we interviewed seven screening services to understand how their screening workflows operate. We were interested to learn more about whether readers are blinded to each others’ reads, how cases are selected for arbitration (e.g. are all recalls arbitrated, or just the discordant reads), how abnormalities are recorded, what information is collected, and whether PACS digital markings are included in their processes. Interviews were conducted virtually with members of screening staff at each screening service, and a summary is presented in Supplementary Table 4.

Workflow mapping design workshops

As described in the study manuscript, we performed a number of workflow mapping design workshops to understand the clinical workflows associated with breast cancer screening in the UK.

Screening Service 2 was selected for AI-integrated workflow design exploration to understand the implications of integrating an AI into the existing double read system. Using existing workflow protocols and interviews with screening hospital staff, including radiologists and administrative staff, we mapped in detail the screening workflow.

Following that, an AI-integrated workflow was codesigned iteratively with AI model experts and screening staff. The design covered the steps for screen-reading and arbitration. The AI workflow was then reviewed in a workshop by the screening team, including the screening director.

Following our iterative design approach with Service 1, we identified three significant changes required to incorporate the AI into the workflow. Firstly, for the majority of reads there is no human second reader as the AI provides that step digitally and updates the NBSS record with the results. Secondly, a human second reader is still required to read the cases that have not been read by the AI (7% of cases). These are automatically flagged in NBSS after the AI has entered its results, as they do not have a second read recorded. Thirdly, this service was keen to retain the paper workflow even with AI integration, to minimise changes and risk of introducing new errors. Screening papers are kept in folders, one for each screening 'clinic', containing screening forms and other administrative papers (Extended Data Figure 8). Since the AI is not able to move and sort paper, the second reader is required to still be responsible for this step. Therefore, after reading, the human second reader would pull out the abnormal cases from a folder of cases and mark each case that requires an arbitration read in an arbitration audit sheet. This extra step would also need to be performed by the second human reader for clinics where the AI had read all cases successfully.

A representative screening team member from Service 1 reviewed the AI-integrated workflow generated in collaboration with Service 2. Overall, few adjustments were made. The team agreed that the productivity gains from introducing AI could be negated by the additional paperwork required, and that a digital workflow would be optimal. Digital workflow management functionality is already possible in the NHS via NBSS, although it has not commonly been used to date (Supplementary Table 4).

A workshop review with Service 1 determined that only minor changes from the final version of the Service 2 workflow would be required to implement the design, despite the two services having different workflow protocols and arbitration strategies. Rather than pulling out all abnormal cases for arbitration, the human second reader would instead need to pull out only discordant cases for arbitration, alongside cases that were already due to be recall

References

1. McKinney, S. M. *et al.* International evaluation of an AI system for breast cancer screening. *Nature* **577**, 89–94 (2020).
2. Kolesnikov, A. *et al.* Big Transfer (BiT): General Visual Representation Learning. in *European Computer Vision Association*.
3. Tan, M., Pang, R. & Le, Q. V. EfficientDet: Scalable and Efficient Object Detection. (2019).
4. Taylor-Phillips, S. *et al.* UK National Screening Committee's approach to reviewing evidence on artificial intelligence in breast cancer screening. *Lancet Digit Health* **4**, e558–e565 (2022).
5. Bennett, R. L., Sellars, S. J. & Moss, S. M. Interval cancers in the NHS breast cancer screening programme in England, Wales and Northern Ireland. *Br. J. Cancer* **104**, 571–577 (2011).

DocuSign Envelope ID: 8C37849F-C375-42F4-8310-CC1C11A57E2D

Imperial College London

Artificial Intelligence in Mammography Study (AIMS)

Clinical validation of an artificial intelligence system to improve the quality, efficiency and experience of breast cancer screening

Protocol v4.0, 21 August 2023

Main sponsor: Imperial College London

Funders: NIHR Artificial Intelligence (AI Award 2020 Phase 3) Competition

IRAS Project ID: 303782

REC reference: 22/EM/0038

Contents

1 Study information **7**

1.1 Key study contacts 7

1.2 Investigators 7

1.3 Funding and support in kind 8

1.4 Role of trial sponsor and funder 8

1.5 Protocol contributors 8

1.6 Patient engagement in research programme 9

1.7 Study steering committee 9

2 Introduction **10**

2.1 Background 10

2.2 Rationale for current study 11

3 Study objectives **12**

3.1 Primary objective 12

3.2 Secondary objectives 12

4 Study design **12**

5 Part A: Retrospective diagnostic accuracy study **13**

5.1 Study aim 13

5.2 Dataset curation and study setting 13

5.3 Ground truth determination 14

5.4 Study endpoints 14

5.5 Participant entry 14

5.5.1 Inclusion criteria 14

5.5.2 Exclusion criteria 15

5.5.3 AI-system eligibility criteria 15

5.6 Methods and statistical analysis 15

5.7 Consent 16

5.8 Presentation of results 16

5.9 Identification of biases 16

6 Part B - Simulated usage of the AI system by readers in arbitration panels using retrospective data **17**

6.1 Study aim 17

6.2 Dataset curation 17

6.3 Study endpoints 18

DocuSign Envelope ID: 8C37849F-C375-42F4-8310-CC1C11A57E2D

6.4 Methods 18
6.5 Statistical analysis 20
6.6 Participant recruitment and identification 22
6.7 Consent 22
6.8 Presentation of results 23
7 Patient and public engagement workshops 24
7.1 Aim 24
7.2 Methods 24
8 Data management 25
8.1 Data flows 25
8.2 Retention of data 26
9 Timescales 27
10 Ethical and regulatory considerations 28
10.1 Research Ethics Committee review 28
10.2 Peer review 28
10.3 Public and patient involvement 29
10.4 Protocol compliance 29
10.5 Monitoring and audit 29
10.6 Indemnity 29
10.7 Sponsor 30
10.8 Funding 30
10.9 Financial and other competing interests 30
10.10 Amendments 30
10.11 Assessment and management of risk 31
10.12 Potential for unintended bias 31
11 Expected outcomes of the study, Patient and Public Engagement and Dissemination 32
12 References 33
13 Appendix 1 34

DocuSign Envelope ID: 8C37849F-C375-42F4-8310-CC1C11A57E2D

This protocol has regard for the HRA guidance and order of content

RESEARCH REFERENCE NUMBERS:

- Public Health England Research Advisory Committee Ref: BSPRAC_0093

TRIAL REGISTRY NUMBER AND DATE: ISRCTN60839016 6th June 2022

DocuSign Envelope ID: 8C37849F-C375-42F4-8310-CC1C11A57E2D

PROTOCOL VERSION NUMBER AND DATE

Version Number	Date	Comment
1.0	5 January 2021	Final protocol submitted to REC
2.0	6 June 2022	Amendment for additional human factors work
3.0	24th November 2022	Follow-up for negative cases changed to 24 months
4.0	21 August 2023	Update information about ISO certification and addition of team member

DocuSign Envelope ID: 8C37849F-C375-42F4-8310-CC1C11A57E2D

Imperial College London

Signature page

The undersigned confirm that the following protocol has been agreed and accepted and that the Chief Investigator agrees to conduct the trial in compliance with the approved protocol and will adhere to the principles outlined in the Medicines for Human Use (Clinical Trials) Regulations 2004 (SI 2004/1031), amended regulations (SI 2006/1928) and any subsequent amendments of the clinical trial regulations, GCP guidelines, the Sponsor's (and any other relevant) SOPs, and other regulatory requirements as amended.

I agree to ensure that the confidential information contained in this document will not be used for any other purpose other than the evaluation or conduct of the clinical investigation without the prior written consent of the Sponsor

I also confirm that I will make the findings of the trial publically available through publication or other dissemination tools without any unnecessary delay and that an honest accurate and transparent account of the trial will be given; and that any discrepancies and serious breaches of GCP from the trial as planned in this protocol will be explained.

For and on behalf of the Study Sponsor:

Signature: Date:

DocuSigned by:
Cheuk Fung Wong
E6ED866F416A4F8...
....

30-Aug-2023
....../....../......

Name (please print):

Cheuk Fung Wong
....

Position: Research Governance and Quality Assurance Manager
....

Chief Investigator:

Signature: Date:

DocuSigned by:
Ara Darzi
447CB46CAAB7464...
....

30-Aug-2023
....../....../......

Name: (please print):

Ara Darzi
....

Imperial College London

1 Study information

1.1 Key study contacts

Chief Investigator	Prof Ara Darzi, Lead for Research Translation, Director Institute of Global Health Innovation, Imperial College London, a.darzi@imperial.ac.uk
Sponsor	Institute of Global Health Innovation, Imperial College London
Funder(s)	NIHR Artificial Intelligence (AI Award 2020 Phase 3) Competition

1.2 Investigators

**Name**	**Specify project role**
Ara Darzi	Overall Chair and Lead for Imperial College London
Hutan Ashrafian	Lead for Research Translation
Deborah Cunningham	Co-lead for Imperial NHS Trust
Hema Purushothaman	Co-lead for Imperial NHS Trust
Mamatha Reddy	Co-lead for St George's NHS Trust
Lisanne Khoo	Co-lead for St George's NHS Trust
Lucy Warren	Lead for Royal Surrey NHS Foundation Trust
Fiona Gilbert	Co-Investigator and Scientific advisor
Anna Lawrence-Jones	PPI Lead, Imperial College
Shravya Shetty	Google Health engineering lead
Christopher Kelly	Google Health clinical lead
Jeremy Miles	Statistician, Google Health

Name(s) and address(es) of all medical and/or technical department(s) and/or institutions involved in the project, including any data processors and/or collaborators that will process the data:

- Imaging and Diagnostics Team, Google Health

Imperial College London

- Department of Surgery, Imperial College London
- Breast Screening Service, Imperial College Healthcare NHS Trust
- Breast Screening Service, St George's University Hospital NHS Foundation Trust
- Department of Medical Physics, Royal Surrey NHS Foundation Trust
- Department of Radiology, Cambridge University

1.3 Funding and support in kind

FUNDER(S)	FINANCIAL AND NON FINANCIAL SUPPORT GIVEN
NIHR Artificial Intelligence (AI Award 2020 Phase 3) Competition	Financial
Google Health	Google staff costs across engineering, research scientists, user experience researchers, information governance team, security and privacy team<br>Technical infrastructure costs

1.4 Role of trial sponsor and funder

Imperial College London will act as the main Sponsor for this study. Delegated responsibilities will be assigned to the NHS trusts taking part in this study. The study Sponsor assumes overall responsibility for the initiation, management and funding of this study. The Sponsor is responsible for the design of this study, in addition to its conduct, data analysis and interpretation, and subsequent manuscript writing and dissemination of study findings. The Sponsor controls the final decision regarding any of these aspects of the study.

Funding - NIHR Artificial Intelligence (AI Award 2020 Phase 3) Competition. Google will not receive grant funding from the AI Award, and will cover the costs of its staff, technology, and infrastructure.

1.5 Protocol contributors

A wide range of disciplines have contributed to protocol development, including breast radiologists, clinical researchers, user experience/human factors researchers, medical physicists, engineering, statisticians, and patient engagement experts:

- Imperial College London: Ara Darzi, Hutan Ashrafian, Rachita Mallya, Anna Lawrence-Jones
- Imperial College Healthcare NHS Trust: Deborah Cunningham and Hema Purushothaman
- St George's University Hospitals NHS Foundation Trust: Mamatha Reddy, Lisanne Khoo

- Royal Surrey NHS Foundation Trust: Mark Halling-Brown, Lucy Warren, Kenneth C. Young
- Cambridge University: Fiona Gilbert
- Google Health: Christopher Kelly, Lee Kupferman, Scott McKinney, Megumi Morigami, Rory Sayres, Amir Kiani, Shravya Shetty, Yetunde Ibitoye, Sam Fishman, Richard Sidebottom, Marcin Sieniek, Reena Chopra, Jeremy Miles

1.6 Patient engagement in research programme

Public representatives have been involved in all our original research. Two lay partners with experience of breast cancer participated in every stage of the initial model development, from training data specification and use-case, to evaluation strategy, iteration of our experiments, and preparing our publications.

We have recently recruited two additional lay partners to the steering committee to expand diversity of input. Lay partners are involved in decision making, design, and dissemination, and have been involved in the development of this research protocol. We have begun a series of workshops with wider groups of people that attend mammography screening or have been affected by cancer to discuss ideas, concerns, and expectations for the project.

1.7 Study steering committee

A study steering committee has been formed consisting of representatives from each organisation, alongside public representatives. This committee will meet at least monthly to ensure that the study is progressing well, review progress, and prepare relevant reports for the Sponsor.

2 Introduction

This project aims to evaluate the potential for artificial intelligence (AI)-enabled NHS breast screening to increase accuracy, safety, cost-effectiveness, and clinician/patient experience, while demonstrating evidence of clinical feasibility.

2.1 Background

1 in 8 women will be diagnosed with breast cancer in their lifetime. Breast screening aims to find cancers early, where treatment is more successful. In the UK, two readers assess each mammogram (x-ray of the breast), with disagreements reviewed by a panel. However, a radiologist workforce crisis threatens our screening programme's long term sustainability.

The UK is facing a 44% shortage in radiologists by 2025 (The Royal College of Radiologists 2021), while it is estimated that only 18% of screening hubs have sufficient staff to cope with current double reading requirements on a 3-yearly cycle (Public Health England 2016). If invitations were to be extended to women aged 47 to 73, as being investigated by the AgeX trial, the demands on the service would increase further (Moser et al. 2011). The negative impact of COVID-19 on breast screening in 2020 only adds to the increased burden expected over the coming years as services catch up. Breast Cancer Now estimated that almost 1 million women have missed their breast screen during the pandemic (Breast Cancer Now 2020), and Macmillan's analysis of the rate of recovery suggests it would take 20 months to work through the current backlog if activity was increased to 10% above pre-pandemic levels (Macmillan Cancer Support 2020). In addition, there are calls for the UK to adopt two-yearly screening, mirroring most other European countries, which would be unfeasible given current workforce pressures, without compromising quality. We believe AI-enabled screening can play an important role in future-proofing the UK's Breast Screening Programme.

Google's Mammo-Reader is an AI-powered independent mammography reader product for double-read breast cancer screening workflows. It analyses two-dimensional full field digital mammography to give a normal/abnormal screening determination, and highlights suspicious regions of interest. In a study published in Nature (McKinney et al. 2020), the AI system was able to demonstrate performance close to that of double reading with arbitration (statistically non-inferior) and superior to the first reader. We believe that deploying this technology as a second reader has the potential to: (1) ultimately improve patient outcomes through improved accuracy and reduced variability; (2) modify the reader / radiologist workload mitigating the current workforce challenges and even allow expansion to alternative screening strategies such as biannual or personalised stratified approaches; and (3) reduce time to results, improving patient experience. In addition, the discrepancy between a radiologist and the AI system is greater than two humans, suggesting that it may be possible to detect a greater number of cancers (higher overall sensitivity), albeit at a cost of greater number of cases for arbitration.

The AI system is intended to be deployed as a second reader within the UK breast screening programme, integrated securely into the National Breast Screening System (NBSS) and clinical imaging PACS. In our primary proposed workflow, a screen reading expert will read all cases first. Once the first reader has submitted their assessment, and eligibility checks have been completed the AI system's assessment will be made available. The AI system's decision will be compared to

the first reader. In the estimated 10% disagreements, these cases will be referred to an arbitration panel for final determination (following existing local site arbitration rules). We believe this workflow promotes safety by ensuring all AI system decisions require independent agreement from human readers to influence a woman's care, and that all disagreements are reviewed by expert arbitration panels.

This mammography product builds upon Google/DeepMind's world-leading expertise in pioneering artificial intelligence technologies, unique experience in scaling multiple products and services to billions of users around the world, and world-leading Cloud infrastructure with significant experience in healthcare applications. Complementing Google's technology platform, Google Health's cross-functional regulatory, information governance, quality and clinical infrastructure provides a unique environment to build safe, secure, robust, reliable, fair, and accurate clinically validated products to achieve patient impact at scale.

The AI system has been developed under a Quality Management System adhering to CE certification requirements, and is currently both ISO 13485 and ISO 27001 compliant.
The certifications will expire in early 2024 after the anticipated study completion date.

2.2 Rationale for current study

This project includes two main components. Firstly, we will compare the AI system to radiologists in large historical populations at two NHS sites with diverse multicultural patient populations. This will allow detailed assessments of accuracy; ensure fair, equitable performance; and enable modelling of workforce and economic impacts. This will complement other evaluation studies being performed by Google.

Secondly, we will perform a large diagnostic study to re-read historical mammograms from approximately 50,000 women across two hospitals to explore how their expert radiologists in arbitration panels interact with the AI system when used in place of the second radiologist. This will explore the complex human factors involved, measure overall system accuracy of AI-enabled screening, and enable assessment of NHS health economic impacts.

Interventional use of the AI system within the health system cannot be commenced before comprehensive modelling of likely clinical, workflow, and economic impacts. This proposal outlines our plans to translate this ground-breaking research towards real world patient impact through retrospective diagnostic accuracy studies and large scale consensus panel reader studies. The next phase of this work will include prospective observational feasibility studies to test the AI system running 'silently' within each hospital. Through this work, we aim to provide the necessary evidence to support progression to future interventional use in a way that delivers measurable benefits to public health.

3 Study objectives

Based upon our recent published work (McKinney et al. 2020) and subsequent progress towards development of a regulated medical device, we hypothesise that:

- Our novel AI system for breast cancer screening demonstrates the appropriate accuracy, safety, acceptability, and cost effectiveness required for use as an independent reader within the NHS breast screening programme

3.1 Primary objective

a) To test accuracy of the system acting as an independent second reader in large retrospective NHS breast screening datasets, compared against historical clinical reader accuracy, using cases from multiple NHS screening sites and mammography devices, assessed against a robust ground truth of biopsy-proven cancer over 39 months.

3.2 Secondary objectives

a) Assess the performance and human factors of a simulated specialist arbitration panel when the AI system is used as the second reader
b) Perform detailed analyses to identify underperformance in clinical or demographic subgroups, and any unintended biases. Understand the types of cancers detected by AI to allow an assessment of potential changes to the balance of benefits and harms including potential overdiagnosis.
c) Perform detailed clinical, workflow, productivity and economic analyses to assess overall benefit to the screening service
d) Understand patient and public ideas, concerns and expectations of AI-enabled breast cancer screening through workshops and surveys

4 Study design

The study consists of two components: Part A: retrospective diagnostic accuracy study, and Part B: simulated usage of the AI system by readers in arbitration panels using retrospective data.

The anticipated study start date is 1st January 2022. The duration of the study is 24 months.

5 Part A: Retrospective diagnostic accuracy study

5.1 Study aim

The retrospective diagnostic accuracy study aims to evaluate accuracy, generalisation and fairness of the AI system, compared to contemporaneous clinical decisions, using large historical pseudonymised screening datasets from at least two UK screening sites with a range of clinical practice, collected previously during routine screening, with subsequent longitudinal cancer follow up. This study will allow a precise evaluation of AI system performance, bias, and safety using datasets where long-term cancer outcomes are available for accurate ground truth determination.

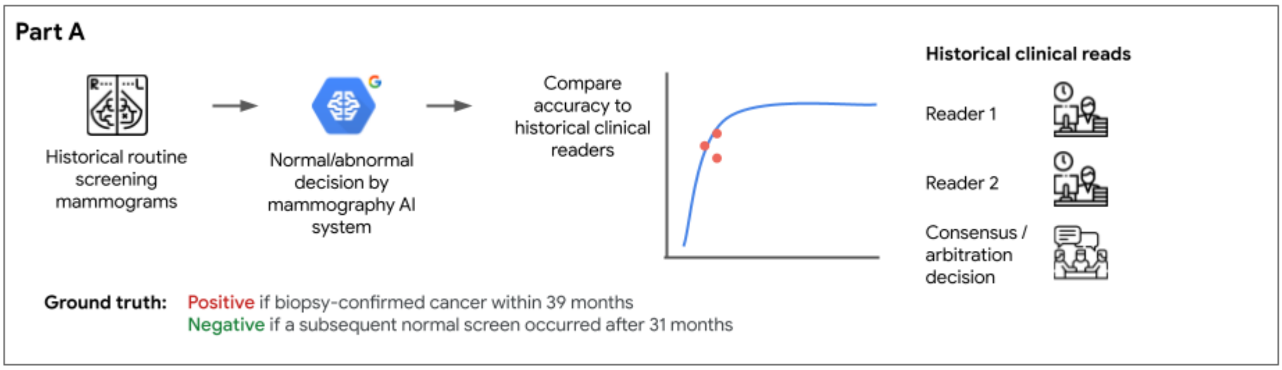

5.2 Dataset curation and study setting

A test dataset from each screening site will include at least 25,000 women per site aged between 50 and 70 who have previously attended for routine screening from January 2016 onwards. Cases will be selected randomly in order to curate a dataset that is representative of the screening population. Datasets will include all screening mammograms relevant to the selected screening visit, including technical recalls comprising mediolateral oblique and craniocaudal views of the left and right breasts, all reader opinions including the arbitration result (if applicable), and metadata associated with follow-up treatment. These data have been collected in the course of normal clinical care, and no primary data will be collected. No data from women included in datasets used for AI system development will be included in these test datasets.

Importantly, imaging and clinical data will be collected under existing ethical permissions for the OPTIMAM database (REC references 14/SC/0258 and 19/SC/0284), using existing infrastructure and technical methodology. No additional permissions are being sought for Part A. Data will be shared with Google Health under existing ethical approvals, existing OPTIMAM steering group approvals, and an existing data sharing agreement.

The OPTIMAM Mammography Image Database (Halling-Brown et al. 2021) is a large curated and centralised database of mammogram images and associated clinical data from multiple NHS Breast Cancer screening sites. The database contains unprocessed and processed medical images, associated expert-determined region of interest annotations and clinical data relating to screen detected and interval cancers. The process of image collection, annotation and storage is almost fully automated and is very adaptable, allowing for quick and easy expansion to disparate imaging sites. The ongoing collection of data is funded by Cancer Research UK and the database

is hosted and managed by Royal Surrey NHS Foundation Trust. The resource has been designed to be shared for research purposes. However, the consortium will update the OPTIMAM database steering committee of the research being undertaken.

Data will be collected from two NHS screening services at St George's University Hospitals NHS Foundation Trust and Imperial College Healthcare NHS Trust. We may enrol additional sites that are part of the OPTIMAM database if required to achieve acceptable confidence intervals and diversity of representation for the study's secondary endpoints.

Google Health will not receive identifiable patient information, and all dataset collection and linkage will take place on hospital premises (see Section 8. Data management).

5.3 Ground truth determination

Cases will be considered positive if they received a biopsy-confirmed diagnosis of cancer within 39 months following the screening visit. Negative cases will require a negative result from the study screening visit, and another negative result at the subsequent screening visit at least 24 months later.

5.4 Study endpoints

Primary endpoints for the study will be:

- AI system cancer detection sensitivity and specificity (diagnostic accuracy matrix) compared to first, second and consensus reader decisions, measured against ground truth definition above.

Secondary endpoints will include:

- Subgroup performance by factors including cancer type and grade, primary tumour size, patient age, breast density, prior cancer, prevalent and incident screens, ethnicity, device manufacturer, socioeconomic status, and screening site.
- AUC-ROC for cancer detection, positive predictive value, negative predictive value, cancer detection rate, case recall rate.
- System performance in confirmed interval cancers (percentage of historical interval cancers that the AI system flagged for recall, and qualitative agreement of the localisation in the original screening mammogram with the presence/absence of true radiological evidence).
- AI system localisation performance (if lesion position data available).
- Analysis of failure cases.
- Percentage of women that meet the eligibility criteria.
- Simulations of workforce impact assessment and health economic modelling.

5.5 Participant entry

5.5.1 Inclusion criteria

- Women undergoing routine breast cancer screening (age 50-70) as part of the national breast screening programme from January 2016 onwards.

- Mammography images acquired using Hologic/Lorad, Siemens, or GE devices.

5.5.2 Exclusion criteria

- None

5.5.3 AI-system eligibility criteria

In the first instance, the AI system is intended for use in routine screening patients that make up the bulk of the screening programme workload. The following criteria apply for a case to be deemed eligible to be read by the AI. The number of cases deemed ineligible will be recorded.

Eligible

- Women undergoing routine breast cancer screening (age 50-70), as part of the national breast screening programme.
- Mammography images acquired using Hologic/Lorad, Siemens, or GE devices.

Ineligible

- Women attending an assessment clinic or symptomatic clinic (i.e. not routine screening).
- Women undergoing annual screening due to:
    - High risk (lifetime risk >30% - e.g. faulty BRCA1, BRCA2, TP53)
    - Moderate risk (lifetime risk 17-30%)
    - Personal stratified follow up (e.g. indeterminate B3 lesions)
- Presence of breast implants.
- Screens with incomplete (<4 standard screening views - e.g. due to abandoned screen)
- Poor diagnostic quality imaging (which would be repeated).
- Non-standard acquisitions beyond the routine 4 screening views.
- For negative or benign cases, women without a negative follow up screen approximately 3 years later (at least 24 months after initial screen), as this would preclude determination of a robust ground truth.

These eligibility checks will be identified by checking DICOM labels (e.g. for implants, views and number of images available) and NBSS reports for technical repeats and recalls, annual screens, and diagnosis and follow-up screen results. Eligibility checks will be applied in future prospective feasibility testing of the AI system within an NHS workflow in a similar manner. In our future work, we plan to address these excluded cohorts to increase the eligible population available to the AI system.

5.6 Methods and statistical analysis

We plan to replicate the methodology used previously at two NHS screening sites with diverse patient populations (McKinney et al. 2020).

Cancer will be defined as biopsy-confirmed cancer within 39 months of index mammogram, while healthy cases will require an additional normal mammogram at the next screen. The study's primary endpoint will be powered at a site level using a cancer sensitivity non-inferiority margin of 5% and target power of at least 80%. This will require approximately 50,000 women overall that meet the eligibility criteria (25,000 per site), with a population prevalence of 400 cancer cases

overall (200 per site). We may enrol additional OPTIMAM sites or collect additional data from existing sites if required to achieve acceptable confidence intervals for our secondary endpoints.

For each site, we will compare the sensitivity and specificity of the readers with that of a pre-specified thresholded score from the AI system. The pre-specified threshold will be based on the performance of the AI system on a separate retrospective dataset that does not overlap with the cases included in this study, and will be optimised to mirror a radiologist in terms of balanced sensitivity and specificity. Confidence intervals on the difference will use Wald intervals (Fagerland, Lydersen, and Laake 2014), and a Wald test will be used for non-inferiority (Liu et al. 2002). Both will use the Obuchowski variance estimate (Obuchowski 1998). For non-inferiority comparisons, we plan to use a 5% absolute margin (as used in our Nature paper, and generally more stringent than the literature). Superiority comparisons on the UK data will be conducted using Obuchowski's extension of the two-sided McNemar test for clustered data. We will use a statistical significance threshold of 0.05, and will correct for multiple comparisons using the Holm–Bonferroni method.

To evaluate the stand-alone performance of the AI system, the area under the receiver operating characteristic (AUC-ROC) curves will be estimated using the normalised Wilcoxon (Mann–Whitney) U statistic (Mann and Whitney 1947). Non-parametric confidence intervals on the AUC-ROC will be computed with DeLong's method (DeLong, DeLong, and Clarke-Pearson 1988).

We will perform an additional analysis using a relaxed ground truth, where a follow up negative screen is not required. This is because women who are attending for their final screen around the age of 70 will not have a confirmatory negative screen. However, non-screen-detected cancers detected within a 3 year window following the screen will be available through NBSS, and so positive cases will still be appropriately recorded.

5.7 Consent

This study will only involve secondary analysis of anonymised datasets curated as part of the OPTIMAM database (REC references 14/SC/0258 and 19/SC/0284). Informed consent is not sought because we do not obtain or store identifiable data. The only processing of patient identifiable data is the de-identification process itself which takes place at the point of collection on a dedicated research server, in an automated manner, performed by the OPTIMAM project under their existing approved processes.

5.8 Presentation of results

Results will be presented graphically through ROC plots and confusion matrices, alongside standard sensitivity, specificity, positive/negative predictive value, cancer detection rate and recall rate measures.

5.9 Identification of biases

To avoid unintended future harm from the AI system, we will carefully analyse the data to identify the presence of any hidden stratifiers in the data that may lead to suboptimal performance in certain patient/disease groups.

6 Part B - Simulated usage of the AI system by readers in arbitration panels using retrospective data

6.1 Study aim

This study of retrospective breast cancer screening mammography aims to understand the impact of introducing an AI system as a second reader into a double reader workflow, through a large-scale end-to-end study of arbitration performed at two diverse NHS sites. Each site will re-assess their own historical arbitration cases in a simulated setting, using their own mammography readers, and their own arbitration clinical pathways as part of the study.

Study aims include:

- To assess end-to-end clinical efficacy of the double-reading screening system using AI as one of the two readers at two hospitals through large-scale simulated arbitration panels.
- To understand if and how UK readers will change their recall behaviour when AI is incorporated into the screening pathway
- To understand the impact on interval cancers of incorporating AI into the pathway
- To understand whether clinical pathway variability in current practice has an impact on cancer detection with AI through inclusion of two screening centres with different clinical workflows
- To understand human factors implications, including identification of potential areas of bias, and develop appropriate mitigations.
- Quantify workflow impacts and perform a full health economic assessment.

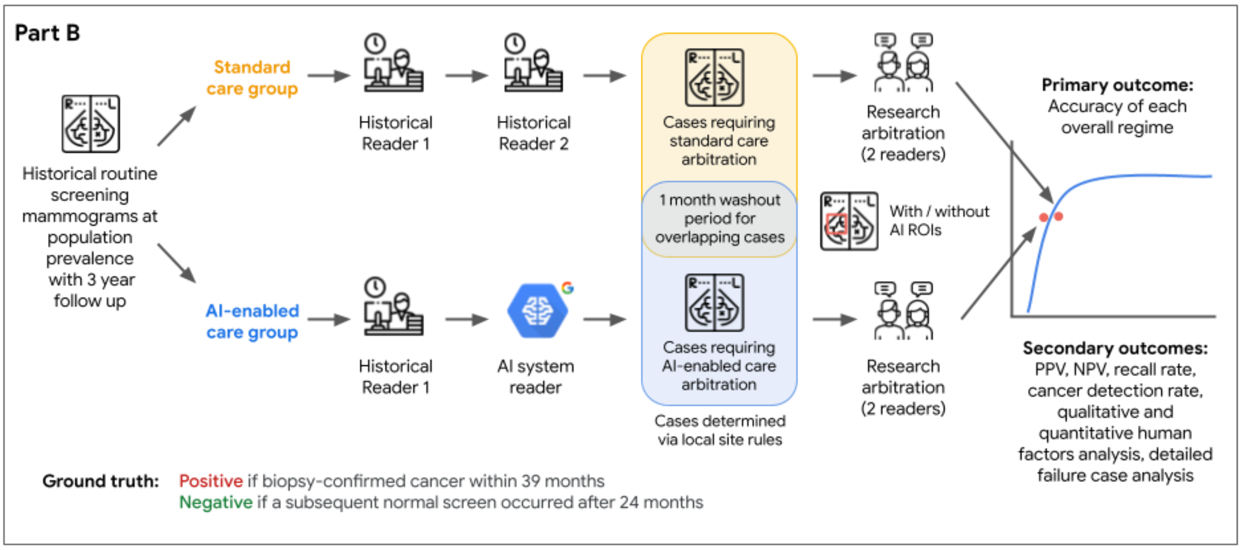

6.2 Dataset curation

Cases in the Part A dataset that previously required arbitration, or that would require arbitration where AI was used as the second reader will be included in the Part B study. Each site will use its own historical data and clinical readers for the reader study.

In order to ensure a realistic arbitration reading setting for the Part B study, we will need to retrieve and transcribe the paper notes that historical readers used to record their opinion onto new study-specific sheets that have been de-identified with a study number only, and only include the required information for each arm of the study. This will ensure that the arbitration sheets are anonymised to the Readers and researchers involved in the study.

NHS Trust-employed research radiographers (part of the care team) will perform the curation of the study arbitration sheets for each case. Patients in the part B study will undergo re-identification by the local site using the pseudonym lookup table accessed by the Trust's research radiographer through the internal web portal of the research server. T
h radiographers account, the web portal will automatically output a pseudonym lookup (NHS numbers and pseudonyms). The research radiographer will then use the NHS numbers (hospital numbers may be required in some circumstances) to access paper and electronic records, for the purpose of transcribing historical clinical notes from prior screening visits into a de-identified format. See Section 8 "Data management" for further details on data processing.

For the Reader study itself, film readers will only access anonymised data.

6.3 Study endpoints

- **Primary endpoints:** Sensitivity and specificity of cancer detection using a 39 month follow up window, compared between the standard care group and the AI-enabled care group.
- **Secondary endpoints including:** Positive predictive value, negative predictive value, cancer detection rate, case recall rate, number of arbitrations in both arms, average time taken to arbitrate a single case, accuracy analysis by all relevant subgroups mentioned in Part A, health economic modelling based upon study findings, difference between study arbitration decisions and historical arbitration decisions, differences in accuracy measures between readers with different levels of experience.

6.4 Methods

- **Dataset:** Same dataset as defined in Part A, with the same inclusion and exclusion criteria.
- **Reader participants:** Radiologist and radiographer film readers will be recruited at each site and grouped into local arbitration pairs. From initial investigations, we anticipate approximately 10 readers grouped into 5 pairs per site will be feasible, performing one research arbitration session of up to 2 hours per week. Each reader will be consented prior to participation. Readers will undertake research sessions outside of their normal working hours so clinical throughput is not impacted. Reading sessions will be paid at the readers' usual rate.
- **Two arbitration methods (standard / AI-enabled):** Cases will be determined for arbitration for both AI-enabled and standard care using local site protocol - for example, St

George's arbitrates all cancer recalls, Imperial arbitrates only discordant recalls. The standard care group is essential to control for the possibility that readers will perform differently in a research setting vs their usual clinical setting.

  - **"AI-enabled care group":** Historical Reader 1 with AI system as Reader 2
  - **"Control standard care group":** Historical clinical Reader 1 and Reader 2

- **Reader study summary:** Readers will be onboarded including practice introductory cases, training to understand the AI system (features, benefits, limitations) and the RiViewer software used for reviewing the cases (see Appendix 1 for more details). Arbitration panels of two readers each will read groups of AI-enabled care cases and standard care cases as they would in clinical practice. Each case will be read with mammography images accompanied by anonymised prior mammograms (if available), historical notes from both original clinical readers (for standard group), or original human reader 1 plus a graphical display of suspicious regions of interest from the AI system (for AI-enabled group). The readers will be asked to complete an electronic Case Report Form for each case, including recall decision, confidence score, highlighted suspicious regions of interest if applicable, along with general comments about the case. For cases included in both groups, cases will be read in a randomised order between arms, with a washout period of at least 1 month between reads.
- **AI viewer:** The AI output will be displayed as a DICOM image on a separate monitor. The output displays the breast-level assessment (either normal or abnormal), and will be the same for both views of the same breast. Regions of interest (ROIs) will be overlaid on the mammogram images, with additional text providing information on the number of ROIs and on which image they have been detected.
- **Human factors research:**
  - Analyses of case report form data, including comments and confidence scores, provided during each arbitration panel decision.
  - Explore additional human factors with readers before, during, and after participating in the study. This may include reviewing additional cases, submitting surveys and/or participating in semi-structured interviews/focus groups about perception of AI vs human reader, such as:
    - Perceived accuracy of the AI system
    - Perceived task load of arbitration with the AI compared to without
    - Perceived self-efficacy for arbitration readers in each arm

    Interviews will be conducted via video call between readers and the research team, and may be audio or video recorded for the purposes of transcription into an anonymised format. Recordings will be deleted after transcription.
- **Post-hoc detailed failure analysis:**
  - Review of failure cases, including using subsequent positive mammograms to identify regions of interest in false negative mammograms.
  - Review of all interval cancers and associated thematic analyses.

    - Quantitative review of arbitration readers' bias towards incorrect AI decisions.
- **Health economic evaluation:**
    - In collaboration with health economist specialists at Google Health and Institute of Global Health Innovation, perform detailed health economic modelling based upon results from Part A and B of this project, in order to provide information to the national screening programme to help evaluate AI technologies.

6.5 Statistical analysis

The arbitration reader study has two arms: traditional workflow (historical R1 + historical R2 → study arbitration) and an AI-enabled workflow (historical R1 + study AI R2 → study arbitration). Each case is evaluated according to both regimes. The study will compare the final screening decisions reached for all cases, including the research arbitration panel if applicable, between the two study arms.

The study will be powered for sensitivity using a 5% non-inferiority margin, with at least 80% power. The same dataset as used in part A of approximately 50,000 women (25,000 from both Imperial College Healthcare NHS Trust and St George's University Hospitals NHS Foundation Trust) that meet the AI eligibility criteria will be included in the study, resulting in an estimated 4,000–6,000 arbitration cases per site, varying depending on local arbitration rules.

Difference between the two arms will be analysed using a matched-pair comparison via a Wald test (Liu et al. 2002). We aim to achieve non-inferiority on sensitivity of cancer detection, so the power calculation is based upon the number of positive cases required. Given the low prevalence of cancer at screening, any specificity comparisons will be abundantly powered.

To perform the power calculation, we first generate R1, R2, and AI model decisions according to the joint distribution observed from a similar OPTIMAM dataset previously used for model validation:

R1	R2	M	*p*
0	0	0	0.23
0	0	1	0.085
0	1	0	0.026
0	1	1	0.046
1	0	0	0.017
1	0	1	0.014
1	1	0	0.089
1	1	1	0.49

Key: 1 = recall, 0 = no recall. n = 1250. p = observed joint distribution in the dataset (# of positive cases where R1=x, R2=y, M=z) / (# of positive cases total)

Next we generate arbitration reads for each arm based on the marginal recall rate observed among arbitrated positives and an agreement parameter *k*. Concretely, this is the rate of agreement between the arbitration reads in the two arms, assuming they were both to arbitrate a given case. We've evaluated values of 0.7, 0.8 and 0.9. We note that the agreement rate between model and arbitration reader on discordant positives is already 68%, so this is likely to set a floor on the actual value of *k*. Our power calculations are therefore based upon a likely lowest agreement value of 70%.

There are two assumptions of this simulation:

1. Cases are arbitrated if:
   a. For Imperial:
      i. Control: R1 ≠ R2
      ii. Experiment: R1 ≠ model
   b. For St George's:
      i. Control: R1 and/or R2 recalls the case
      ii. Experiment: R1 and/or model recalls the case
2. The arbitration decision is independent of *which* of R1 or R2 (or the model) asked for recall.

Imperial College Healthcare NHS Trust: only discordant cases are arbitrated

Simulation results where only discordant cases are arbitrated are as follows:

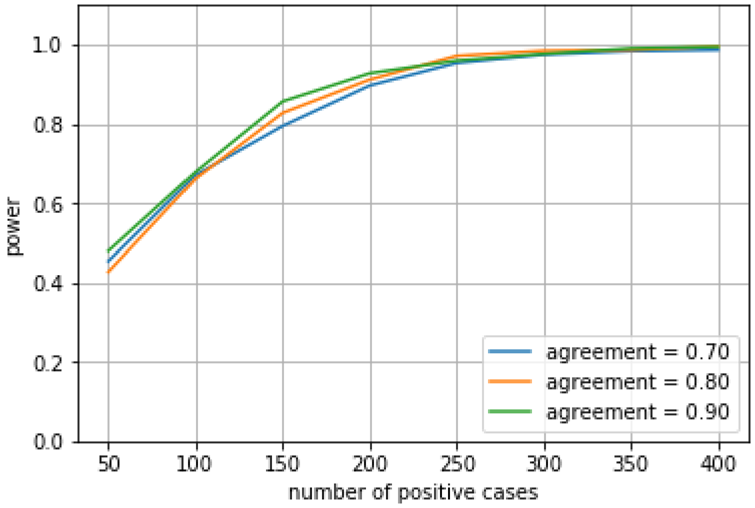

Under this arbitration regime, our modelling suggests that the power is relatively robust to the agreement parameter.

Therefore for any reasonable agreement value above 70%, the study is expected to achieve at least 90% power with minimum 200 cancer positive cases from Imperial.

St George's University Hospitals NHS Foundation Trust: all recalls are arbitrated

Simulation results where all cancer recalls are arbitrated are as follows:

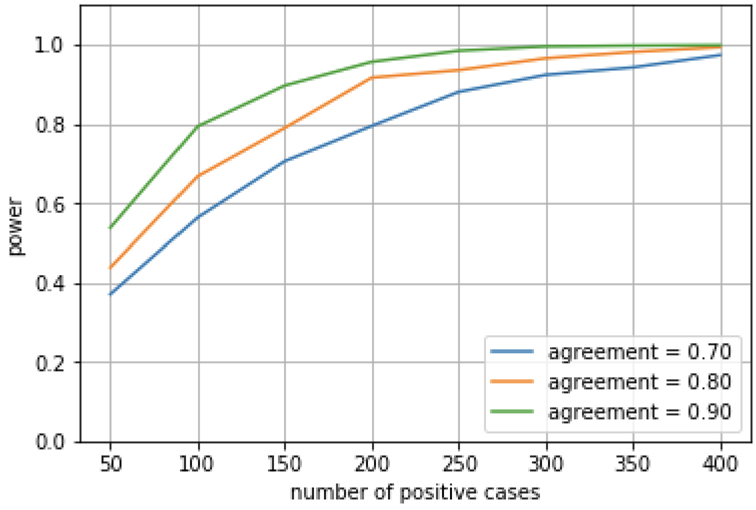

In this setting where all recalls are arbitrated, the power appears more dependent on the rate of agreement between the arbitration reads in the two arms, assuming both arms arbitrate a given case. We do not yet have pilot data to reliably estimate this agreement rate, and there are no previous studies upon which we can base our assumptions. Assuming an agreement of 80%, the study is expected to achieve 90% power with 200 cancer positive cases from St George's. If the agreement between arms was 70% (our previously stated expected minimum), then 200 cancer positive cases would still give the study at this site 80% power.

6.6 Participant recruitment and identification

Screening participants: All data used for this study will be historical and collected in the course of routine clinical care. The OPTIMAM team at Royal Surrey NHS Foundation Trust will select cases and collect data for this study through the OPTIMAM project. Participants will be selected from the available routine screening population who meet eligibility criteria in order to achieve target sample sizes and to be representative of the screened population. Publicity including posters at screening sites will inform patients of the OPTIMAM project (through which the data will be collected).

Film reading readers: Participants in the reader study will be voluntarily recruited from both clinical sites. Readers must be either breast screening radiologists or film reading radiographers that participate in arbitration. There is no other exclusion criteria. The sites involved in this study will be funded for the readers' time through the AI Award. There will be no direct incentives provided to the readers. Readers can withdraw at any time without giving a reason, but any information about the readers or any research data that has already been provided will be kept and used for analysis.

6.7 Consent

Screening participants: Informed consent is not sought because we do not obtain or store identifiable data. This study will involve secondary analysis of anonymised imaging datasets

curated as part of the OPTIMAM database (REC references 14/SC/0258 and 19/SC/0284). OPTIMAM's de-identification process takes place at the point of collection on a dedicated research server, in an automated manner, performed by the OPTIMAM project under their existing approved processes. Posters and information are clearly available at screening sites to notify women of the sites' involvement in this project. In addition to the OPTIMAM dataset, Trust research radiographers will curate de-identified arbitration working sheets from historical records. Once curated, these de-identified sheets would be considered anonymised to the Readers and research team, as they do not have access to the pseudonym keys required.

Film reading readers: Readers will be asked to consent before involvement in the reader study. Participant information will be provided with a concise summary of their involvement in the study, including familiarisation with this protocol. Informed consent forms will be completed before beginning the study.

6.8 Presentation of results

On completion of the study, the data will be analysed and tabulated, and a final study report will be prepared. The study report will be published in a peer-reviewed academic journal with open access as soon as possible following the report finalisation.

Imperial College London

7 Patient and public engagement workshops

7.1 Aim

- To understand public/patient ideas, concerns and expectations about the use of AI in breast screening mammography, in parallel with Parts A and B.

7.2 Methods

We successfully received Imperial College London Research Ethical Committee Approval on 17/02/2021 to conduct patient and public involvement and engagement workshops for our study titled 'Assessment of patient and public acceptability of an artificial intelligence (AI) based mammographic screening tool to improve quality, efficiency and experience of breast cancer screening' (ICREC reference: 21IC6635). We have started and will continue to run workshops with diverse groups of up to 14 participants who have had experience of breast cancer or have previously experienced routine mammography screening. These workshops are co-facilitated by a lay partner. We will discuss patients' ideas, concerns and expectations for the project, which will feed into project design. All lay partners will be appropriately trained and have access needs supported. Due to COVID-19, we may have to carry out meetings remotely. To ensure our opportunities are inclusive, we will add a phone number to the workshop advert and advertise through community groups. We will send guidance and pay for any dongles for those without internet access. Members will be paid £25/hour and £5 for any online interaction. We will evaluate the impact of our PPI using the GRIPP2 academic tool and asking for feedback.

DocuSign Envelope ID: 8C37849F-C375-42F4-8310-CC1C11A57E2D

8 Data management

Data management for OPTIMAM which supplies data required for the retrospective study (part A) and reader study (part B) are described in detail in the separate “Management protocol for the OPTIMAM Image Database” v2.6 (14/10/2019, REC reference 19/SC/0284). Briefly, this protocol provides a comprehensive outline of the purpose, operation, methods, policies and governance of the OPTIMAM Image Database. It describes the procedures used to collect and store mammographic images for research.

Data will be pseudonymised by the Royal Surrey NHS Foundation Trust team at the point of collection using DICOM 142 supplement compliance tools. This supplement is a standard for de-identification of data in DICOM files. Pseudonym lookup tables will be maintained on secure servers at the clinical collection sites and access will be restricted to staff involved in the patients' clinical care or data managers with specific approval to access the patient data for the purpose of pseudonymisation. The data managers will have NHS letters of access permitting the level of access required for the pseudonymisation process. All data shared with research staff will have already been de-identified and they will have no access to the pseudonym lookup tables. To avoid re-identification the patient data within the database is restricted to year of birth and sex. Attempts to identify subjects are forbidden by data access agreements. Access to the pseudonymisation lookup lists held securely at the clinical sites is limited to staff with responsibility for clinical care or data managers with specific approval from the clinical site.

8.1 Data flows

For both screening sites, Imperial College Healthcare NHS Foundation Trust and St George’s University Hospitals NHS Foundation Trust, data from the NBSS database (1) and PACS (2) will be curated and pseudonymised (4) under existing ethical permissions for the OPTIMAM database (OMI-DB) (REC reference 19/SC/0284), using existing infrastructure and technical practices. This retrospective dataset will undergo pseudonymisation a second time by the OPTIMAM team, providing a unique set of anonymised identifiers for the purpose of this study (5). The dataset will then be transferred to and analysed by the Google AI system on secure cloud computing infrastructure (6, 7, 8).

DocuSign Envelope ID: 8C37849F-C375-42F4-8310-CC1C11A57E2D

Data flow diagram for both Part A and B

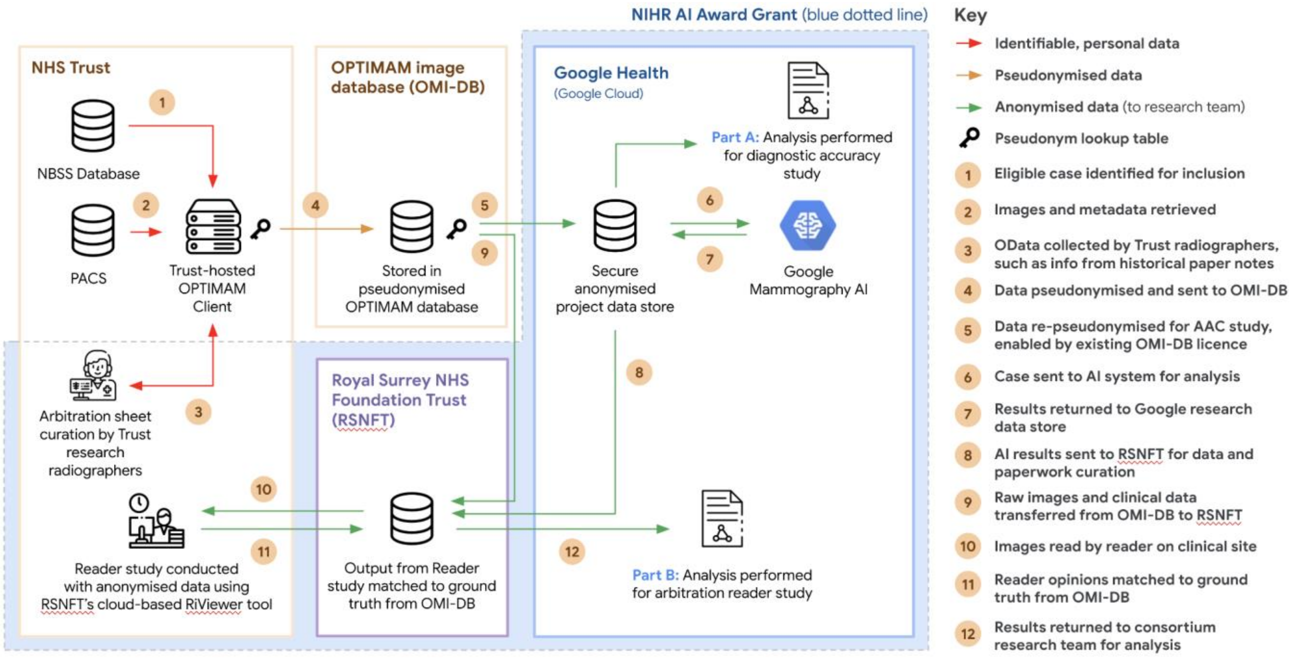

For Part B, an estimated 10% of cases will undergo arbitration in a reader study. For this work, these cases will be re-identified at each Trust using the pseudonym lookup table provided by OMI-DB to the local clinical sites. Only the local clinical sites will carry out the re-identification, and any identifiable data will only be accessible by the Trust research radiographer. The research radiographer will use the NHS Number (hospital number may be required in some circumstances) to access the paper or electronic screening records for these patients to transcribe historical clinical notes from prior screening visits onto a new anonymised format to ensure blinding to the original human read (3). The data will be pseudonymised again by the OPTIMAM team for the reader study, retaining the unique set of anonymised identifiers as for Part B (4, 5). The dataset will be transferred to secure Google Cloud storage, and onwards to Royal Surrey NHS Foundation Trust (8) to conduct the reader study at the local Trust sites using their cloud-based RiViewer software (see Appendix 1 for more details) (9). The output from the reader study will be matched to the ground truth from the OMI-DB (10), and returned to Google Cloud storage (11) for analysis of the collected data.

8.2 Retention of data

Anonymised research data generated by the study will be archived after study completion for a period of 10 years, as per Imperial College London policy. Data will be stored securely at the Institute of Global Health Innovation at Imperial College London. Access will be granted to the Principal Investigator and their deputies only, with security controlled through two-factor authentication. Google Health will retain anonymised study data for a maximum period of 10 years. The study dataset will be stored in dedicated, encrypted, secure health research storage, with two-factor authentication, and strict access control lists that are limited to researchers directly working on this research study.

DocuSign Envelope ID: 8C37849F-C375-42F4-8310-CC1C11A57E2D

9 Timescales

The study design and site set up commenced in 2021. The studies described in this protocol are planned for 2022/23. The planned timescale and milestones are described in the following chart:

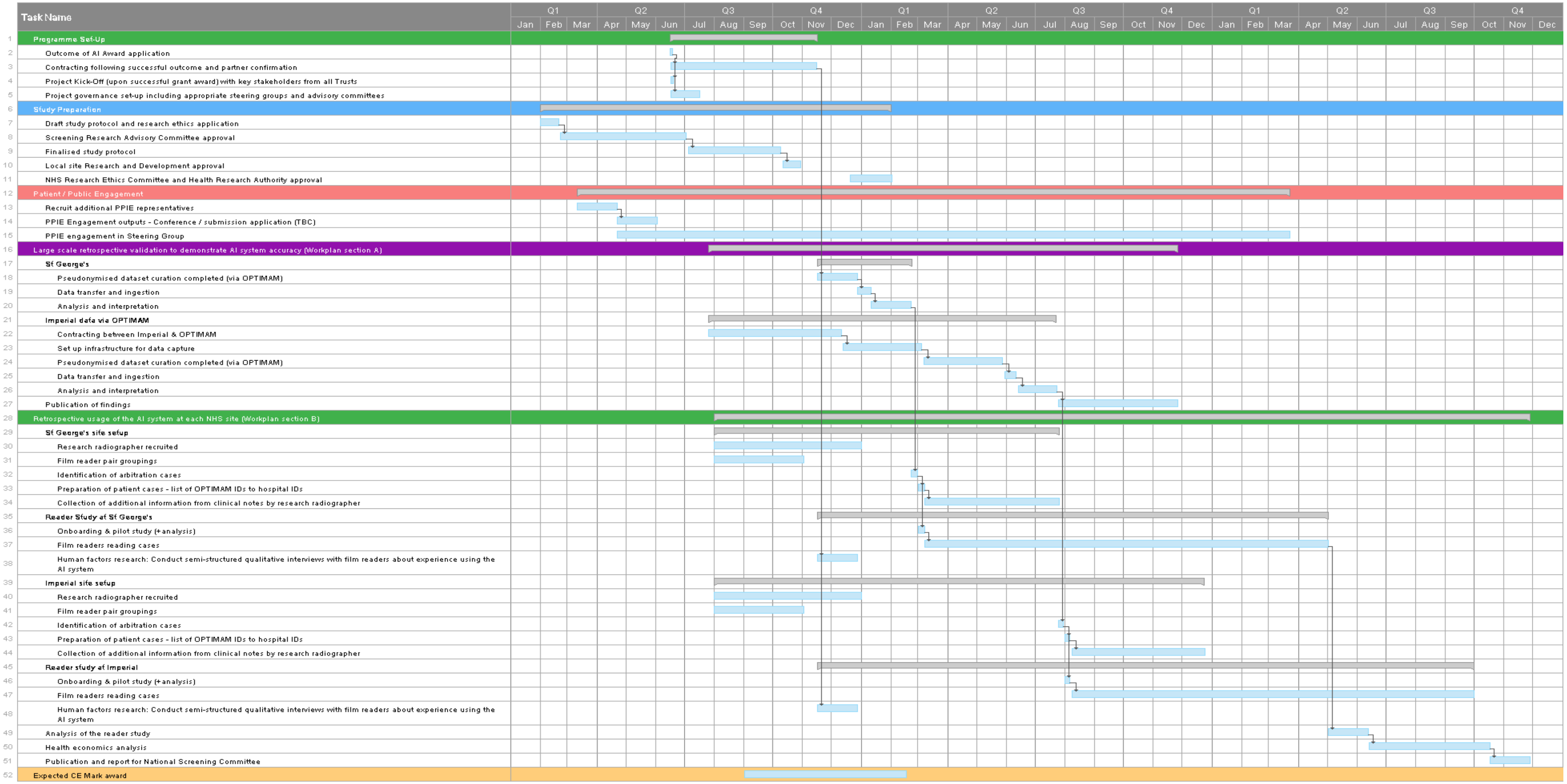

10 Ethical and regulatory considerations

10.1 Research Ethics Committee review

- The Study Coordination Centre has obtained approval from the Nottingham Research Ethics Committee (REC) and Health Research Authority (HRA) for the trial protocol, and other relevant documents.
- The study must also receive confirmation of capacity and capability from each participating NHS Trust before accepting participants into the study or any research activity is carried out. The study will be conducted in accordance with the recommendations for physicians involved in research on human subjects adopted by the 18th World Medical Assembly, Helsinki 1964 and later revisions.
- Substantial amendments that require review by REC will not be implemented until the REC grants a favourable opinion for the trial.
- All correspondence with the REC will be retained in the Trial Master File/Investigator Site File.
- An annual progress report (APR) will be submitted to the REC within 30 days of the anniversary date on which the favourable opinion was given, and annually until the trial is declared ended
- It is the Chief Investigator's responsibility to produce the annual reports as required.
- The Chief Investigator will notify the REC of the end of the study
- If the study is ended prematurely, the Chief Investigator will notify the REC, including the reasons for the premature termination
- Within one year after the end of the trial, the Chief Investigator will submit a final report with the results, including any publications/abstracts, to the REC.

10.2 Peer review

This study is funded by the Artificial Intelligence in Health and Care Award. As part of the application process for this award, the study underwent independent external review by the Accelerated Access Collaborative in partnership with NHSX and the National Institute for Health Research (NIHR), in addition to the Breast Screening Research Advisory Committee (RAC). The protocol underwent further review by the NHS Accelerated Access Collaborative Evaluation Advisory Group (EAG) in December 2021. The study has been reviewed by the Imperial College London Research Governance & Integrity (RGIT) peer review service.

10.3 Public and patient involvement

From project inception in 2016, we have had 2 lay partners with personal experience of breast cancer as part of a committee comprising approximately 10 UK breast screening radiologists and academics. They were involved in every stage of the initial development, from training data specification and use-case, to evaluation strategy and iteration of experiments, and when writing the manuscript for our publication. This ensured that our AI system meets the needs of the screening programme, radiologists and patients alike - classifying occurrence of cancer within 3 years (designed around the NHS programme interval), acting as second reader (advised by Public Health England as their most impactful application), and validated in a large representative dataset.

We will meaningfully involve lay partners throughout this project. We have recruited 2 additional lay partners to join our steering committee to increase the diversity of voices and in case members cannot attend. Lay partners will be directly involved in decision making, design and dissemination of findings. In addition, they will provide input into infographics and blogs aimed at the general public.

All lay partners will be appropriately trained and have access needs supported. Due to COVID-19, we may have to carry out meetings remotely. To ensure our opportunities are inclusive, we will add a phone number to the workshop advert and advertise through community groups. We will send guidance and pay for any dongles for those without internet access. Members will be paid £25/hour and £5 for any online interaction. We will evaluate the impact of our PPI using the GRIPP2 academic tool and asking for feedback.

10.4 Protocol compliance

Prospective, planned deviations or waivers to the protocol are not allowed under the UK regulations on Clinical Trials and must not be used.

Accidental protocol deviations must be adequately documented on the relevant forms and reported to the Chief Investigator and Sponsor immediately.

10.5 Monitoring and audit

The study may be subject to inspection and audit by Imperial College London under their remit as sponsor and other regulatory bodies to ensure adherence to GCP and the UK Policy Framework for Health and Social Care Research. The Chief Investigator will be responsible for the monitoring of the study.

10.6 Indemnity

- Given this is a data-only study, with no direct contact with participants, the potential legal liability of the sponsor(s) for harm to participants arising from the management, design, and conduct of the research is felt to be minimal.
- The study only involves sites that are covered by the NHS indemnity scheme.

- Imperial College holds Public Liability ("negligent harm") and Clinical Trial ("nonnegligent harm") insurance policies which apply to this trial. If a participant can demonstrate that they experienced harm or injury as a result of their participation in this trial, they will be eligible to claim compensation without having to prove that Imperial College is at fault. If the injury resulted from any procedure which is not part of the study, Imperial College will not be required to compensate them in this way. The participant's legal rights to claim compensation for injury where they can prove negligence are not affected.
- In addition, the study and protocol will be submitted to each participating site's Research & Development Department for the usual legal approvals.

10.7 Sponsor

Imperial College London will act as the main Sponsor for this study. Delegated responsibilities will be assigned to the NHS trusts taking part in this study.

10.8 Funding

NIHR Artificial Intelligence (AI Award 2020 Phase 3) Competition is funding the study. This includes funding to collect the datasets and run the studies at the NHS sites. Google will not receive grant funding from the AI Award, and will cover the costs of its staff, technology, and infrastructure.

10.9 Financial and other competing interests

Members of the study group will be required to declare:

- ownership interests that may be related to products, services, or interventions considered for use in the trial or that may be significantly affected by the trial,
- commercial ties requiring disclosure include, but are not restricted to, any pharmaceutical, behaviour modification, and/or technology company
- any non-commercial potential conflicts e.g. professional collaborations that may impact on academic promotion.
- A record of declarations will be kept by the Chief Investigator.

10.10 Amendments

- Amendments will be submitted to the relevant REC using forms provided by the HRA.
- The Chief Investigator will be responsible for the decision to amend the protocol and for deciding whether an amendment is substantial or non-substantial.
- Copies of amendment documentation will be provided to relevant stakeholders including R&D departments at each site.
- A record of changes will be maintained by the Chief Investigator.

10.11 Assessment and management of risk

Part A and B are studies using retrospective data >3 years old, and the algorithm will have no impact or influence on routine clinical care received by patients enrolled in this study. All women included in these studies will have had a subsequent screening round to qualify for inclusion, and so previously missed cancers are expected to be identified at this subsequent round. For this reason, the AI system and study is not considered to pose a direct risk to participants or the care they receive.

Ensuring the highest level of data security and privacy is a fundamental underlying theme of this project, as described in detail in Section 8. Great care will be taken to minimise any potential risk to patients due to data breach.

10.12 Potential for unintended bias

Google Health is very conscious of the potential for AI systems to propagate biases in healthcare. Blind spots in AI systems can reflect the worst societal biases, with a risk of unintended or unknown accuracies in minority subgroups. As a result, the AI system has been developed through the curation of large training datasets from two UK breast screening sites, encompassing scans of patients thought to closely represent the overall UK breast screening population. We believe that this methodology has minimised the possibility of algorithmic biases to date, and we have explored algorithm performance across many clinical and demographic subgroups.

Despite this, we need to achieve greater confidence about the AI system’s performance across various underrepresented subgroups that were not possible due to limited sample size in the original paper. Through the studies outlined in this application, we plan to more rigorously explore the performance of the system in larger, more diverse datasets, in order to ensure that any product created is safe for future deployment at scale.

11 Expected outcomes of the study, Patient and Public Engagement and Dissemination

Through this project we aim to gather evidence of standalone accuracy from two large scale validations of the AI system, quantify human factors when being used by clinicians, and quantify workflow impacts and perform a full health economic assessment. For the next stage of this work, we plan to demonstrate feasibility of real world integration into live clinical systems. We hope this strategy will provide appropriate evidence of feasibility, efficacy and safety for the National Screening Committee to consider a future major modification to the screening programme. This work will also provide evidence for the wider clinical community to understand the impacts of this technology. This project seeks to understand factors that influence public engagement in AI technology, and what may foster better confidence in AI systems.

This study has clear anticipated public health benefits. Results from clinical, workflow and economic analyses will be used to design future interventional studies, and make recommendations about safe future integration of the AI system into routine clinical practice, supporting decision making by NHS England and the National Screening Committee. All results will also be presented at a formally designated Patient and Public Involvement and Engagement (PPIE) group whose interim and final meeting comments will be audited, presented to the steering committee and included in the final presentation of this work.

We plan for this work to be published in peer-reviewed academic journals with open source access as soon as possible following completion, following the forthcoming STARD-AI guidelines for diagnostic accuracy in AI studies (being led by members of this project (Sounderajah et al. 2020)). It is intended that the study will be published as a multicentre study. Findings will also be disseminated through infographics and blogs aimed at the general public, with the assistance of our patient and public representatives.

DocuSign Envelope ID: 8C37849F-C375-42F4-8310-CC1C11A57E2D

12 References

Breast Cancer Now. 2020. “Press Play: Getting and Keeping Breast Cancer Services Back on Track.” https://breastcancernow.org/sites/default/files/final_breast_cancer_now_press_play_report.pdf

DeLong, E. R., D. M. DeLong, and D. L. Clarke-Pearson. 1988. “Comparing the Areas under Two or More Correlated Receiver Operating Characteristic Curves: A Nonparametric Approach.” *Biometrics* 44 (3): 837–45.

Fagerland, Morten W., Stian Lydersen, and Petter Laake. 2014. “Recommended Tests and Confidence Intervals for Paired Binomial Proportions.” *Statistics in Medicine*. https://doi.org/10.1002/sim.6148.

Halling-Brown, Mark D., Lucy M. Warren, Dominic Ward, Emma Lewis, Alistair Mackenzie, Matthew G. Wallis, Louise S. Wilkinson, Rosalind M. Given-Wilson, Rita McAvinchey, and Kenneth C. Young. 2021. “OPTIMAM Mammography Image Database: A Large-Scale Resource of Mammography Images and Clinical Data.” *Radiology. Artificial Intelligence* 3 (1): e200103.

Liu, Jen-Pei, Huey-Miin Hsueh, Eric Hsieh, and James J. Chen. 2002. “Tests for Equivalence or Non-Inferiority for Paired Binary Data.” *Statistics in Medicine* 21 (2): 231–45.

Macmillan Cancer Support. 2020. “The Forgotten ‘C’? The Impact of Covid-19 on Cancer Care.” https://www.macmillan.org.uk/assets/forgotten-c-impact-of-covid-19-on-cancer-care.pdf.

Mann, H. B., and D. R. Whitney. 1947. *On a Test of Whether One of Two Random Variables Is Stochastically Larger Than the Other*.

McKinney, Scott Mayer, Marcin Sieniek, Varun Godbole, Jonathan Godwin, Natasha Antropova, Hutan Ashrafian, Trevor Back, et al. 2020. “International Evaluation of an AI System for Breast Cancer Screening.” *Nature* 577 (7788): 89–94.

Moser, Kath, Sarah Sellars, Margot Wheaton, Julie Cooke, Alison Duncan, Anthony Maxwell, Michael Michell, et al. 2011. “Extending the Age Range for Breast Screening in England: Pilot Study to Assess the Feasibility and Acceptability of Randomization.” *Journal of Medical Screening* 18 (2): 96–102.

Obuchowski, Nancy A. 1998. “On the Comparison of Correlated Proportions for Clustered Data.” *Statistics in Medicine*. https://doi.org/10.1002/(sici)1097-0258(19980715)17:13<1495::aid-sim863>3.0.co;2-i.

Public Health England. 2016. “NHS Breast Screening Programme: National Radiographic Workforce Survey 2016.” https://assets.publishing.service.gov.uk/government/uploads/system/uploads/attachment_data/file/564515/Final_radiographic_workforce_report_25-10-16_colinbabb_gateway_number_2016416.pdf.

Sounderajah, Viknesh, Hutan Ashrafian, Ravi Aggarwal, Jeffrey De Fauw, Alastair K. Denniston, Felix Greaves, Alan Karthikesalingam, et al. 2020. “Developing Specific Reporting Guidelines for Diagnostic Accuracy Studies Assessing AI Interventions: The STARD-AI Steering Group.” *Nature Medicine* 26 (6): 807–8.

The Royal College of Radiologists. 2021. “Clinical Radiology: UK Workforce Census 2020 Report.” https://www.rcr.ac.uk/system/files/publication/field_publication_files/clinical-radiology-uk-workforce-census-2020-report.pdf.

Imperial College London

13 Appendix 1

RiViewer PACs-less, Vendor-Neutral Image Viewer
RiViewer is a bespoke software tool developed by the Royal Surrey NHS Foundation Trust which enables remote observer studies, collaborative viewing sessions and training. It is an application designed to allow workstation-independent, PACS-less viewing and interaction with de-identified medical images (e.g., for observer studies). RiViewer is a standalone tool designed to stream images from a cloud environment to a Radiology Workstation to allow study participants to view medical images, interact with them on studies and annotate ground truth.

Regions of interest (ROIs) can be identified by a user and any associated information about a mark, an image or a study can be added. The questions and settings can be easily configured by the researcher depending on the needs of the research. The extensible nature of the design allows for specific functionality and hanging protocols to be available for each study. Panning, windowing, zooming, and moving through slices are all available while modality-specific features can be easily enabled e.g., quadrant zooming in mammographic studies.

RiViewer is designed to stream images from cloud storage, allowing remote access by radiologists at any site with a workstation and internet connection. Due to the advanced workstation-style functionality, the simple deployment on heterogeneous systems over the internet RiViewer has been used for running remote paperless observer studies and can provide a training infrastructure and coordinating remote collaborative viewing sessions.

For Part B, RiViewer will be used to allow image readers at the clinical sites to define the ground truth on the images collected (locations of biopsy-proven lesions) and to view the images during both arms of the arbitration study. In the AI-enabled group, this will involve displaying the overlaid secondary capture objects outputted by the AI system (the location-based prediction).

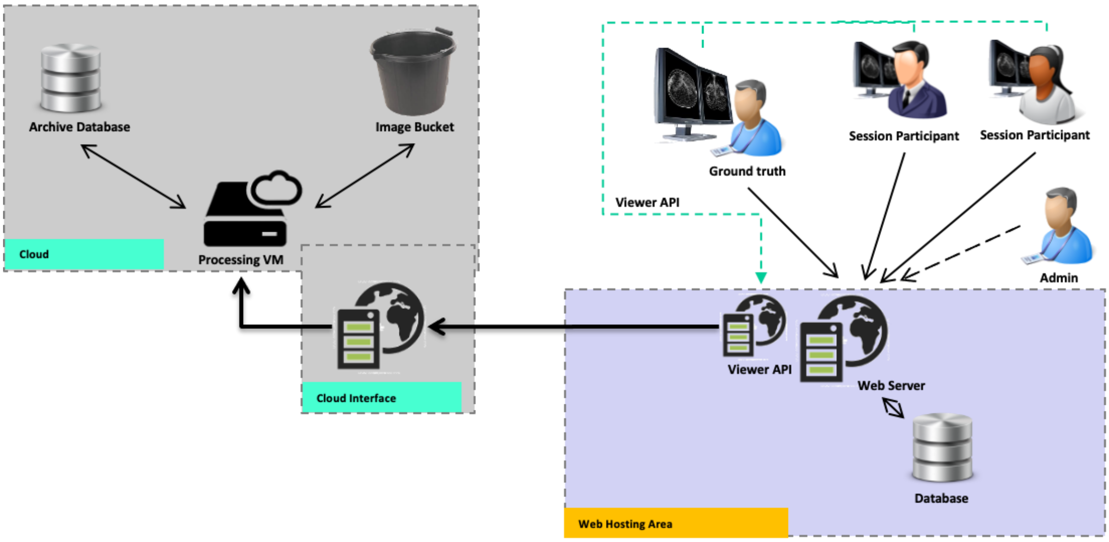

Figure 1: RiViewer Dataflows

RiViewer is a standalone executable, which launches a compiled Java program, bundled with its own Java Runtime Environment. This means that RiViewer does not need installation, does not create any system files, or interact with any system files. It runs within its own folder system. This makes it very simple to install on a workstation. The Zip file needs to be downloaded and extracted in a suitable location on the file system.

Reading sessions (for the arbitration study or ground truth) are prepared in the cloud. A user logs into RiViewer, which queries the cloud API to discover the reading session assigned to that user. The session properties file is downloaded which contains a list of the images that make up the session. RiViewer uses the user authentication tokens to request the images from the cloud and stream them to the local system. Users can view existing ROIs or overlays and are able to draw new ROIs and answer questions. The ROIs or questions answered are sent to the cloud API for storage in the reading sessions database.

DocuSign Envelope ID: 3755EDA5-CDD3-4119-BD41-A6AF70DAAAAC

Artificial Intelligence in Mammography Study (AIMS) Part C

Feasibility of an artificial intelligence system to improve the quality and efficiency of breast cancer screening

Protocol v4.0, 09 August 2023

**Sponsor**	Imperial College London
**Sponsor reference**	22SM7705
**Funder**	National Institute for Health Research-NHSx/Accelerated Access Collaborative
**Funder reference**	AI_AWARD02288
**IRAS number**	307842
**ISRCTN**	88754382

Contents

1 Study and general information 8
1.1 Sponsor 8
1.2 Funding 8
1.3 Trial Registration 9
1.4 Research Reference Numbers 9
1.5 Trial Administration 9
1.6 Key study contacts 9
1.7 Investigators 10
1.8 Protocol contributors 11
1.9 Summary of Trial 11
1.10 Lay summary 13
2 Introduction 14
2.1 Background 14
2.2 Rationale for current study 15
3 Study objectives 16
3.1 Primary objectives 16
3.2 Secondary objectives 16
4 Selection of Sites and Investigators 17
4.1 Principal investigator's qualifications & agreements 17
4.2 Adequate resources 18
4.3 Required trial documentation 18
4.4 Approval and activation 18
5 Participant entry 19
5.1 Inclusion criteria 19
5.2 Exclusion criteria 19
6 Assessment and follow-up 19

7 Study design and setting 19

7.1 Integration with hospital IT systems for automated metadata and image collection via Royal Surrey SMART box 20

7.2 AI system 21

7.3 Web PACS UI 22

7.4 Research-only Trust hosted DICOM store 22

7.5 Data flow diagram 23

7.6 AI-system eligibility criteria 24

7.7 Study sites 25

7.7.1 St George's University Hospitals NHS Foundation Trust 25

7.7.2 Imperial College Healthcare NHS Trust 26

7.8 Pilot of technical integration 27

7.9 Main feasibility study 27

7.10 End of study definition 27

7.11 Study data collected 28

7.12 Study outcome measures 33

7.12.1 Primary outcome measures 33

7.12.2 Secondary outcome measures 33

7.13 Statistical analysis 33

7.14 Presentation of results 34

8 Consent 34

8.1 Opt-out process 35

9 Data management 36

9.1 Data encryption and security 36

9.2 Integration qualification process 37

9.3 Data storage at study completion 38

10 Oversight and Trial Committees 38

10.1 Trial Management Group (TMG) 38

10.2 Trial Steering Committee (TSC) 39
10.3 (Independent) Data Monitoring Committee (I[DMC]) 39
10.4 Patient and Public Involvement Advisory Groups 39
10.5 Role of Study Sponsor 39
11 Patient and Public Involvement and Engagement (PPIE) 39
11.1 PPIE Strategy 39
11.2 PPI contributors 40
11.3 Protocol design and study setup 40
11.4 PPI in the ongoing running of study 40
11.5 PPIE Workshops 41
11.5.1 Aim 41
11.5.2 Methods 41
11.6 Reporting and evaluating impact of PPI 41
12 Ethical and Regulatory considerations 41
12.1 Regulatory compliance 42
12.2 Protocol compliance 42
12.3 Site compliance 42
12.4 Ethical conduct 42
12.5 Research Ethics Committee review 43
12.6 Peer review 43
12.7 Indemnity 44
12.8 Financial and other competing interests 44
12.9 Protocol amendments 44
13 Quality Assurance and Control 44
13.1 Risk assessment and management of risk 44
13.2 Potential for unintended bias 45
13.3 Monitoring and audit 45
13.4 Confidentiality 46

DocuSign Envelope ID: 3755EDA5-CDD3-4119-BD41-A6AF70DAAAAC

14 Expected outcomes of the study, Patient and Public Engagement and Publication and Dissemination of results 46

15 References 48

16 Appendix 1 49

16.1 De-identification of DICOM Images 49

16.1.1 Pseudonymisation of DICOM tags 49

16.1.2 Private tags 49

16.1.3 UIDs 49

16.1.4 Nulling of DICOM tags 49

PROTOCOL VERSION NUMBER AND DATE

Version Number	Date	Comment
1.0	1st August 2022	Submitted to REC / CAG
2.0	4th October 2022	HRA approved version
3.0	22nd December 2022	- Clarification that integration system and Cloud-based Mammo-Client software is not CE certified (section 2.1)<br>- Change of secondary outcome measures to exploratory
4.0	09th August 2023	- CE marking for AI tool wording change<br>- Data flow diagram inserted as picture as original figure was missing wording and arrows<br>- Addition of new trial statistician

Imperial College London INSTITUTE OF GLOBAL HEALTH INNOVATION

Google Health

NHS St George's University Hospitals NHS Foundation Trust

NHS Royal Surrey NHS Foundation Trust

NHS Imperial College Healthcare NHS Trust

Signature page

The undersigned confirm that the following protocol has been agreed and accepted and that the Chief Investigator agrees to conduct the trial in compliance with the approved protocol, GCP guidelines, the Sponsor's (and any other relevant) SOPs, and other regulatory requirements as amended.

This protocol describes the AIMS Part C study and provides information about procedures for entering participants. Every care was taken in its drafting, but corrections or amendments may be necessary. These will be circulated to investigators in the study. Problems relating to this study should be referred, in the first instance, to the Chief Investigator.

This study will adhere to the principles outlined in the UK Policy Framework for Health and Social Care Research. It will be conducted in compliance with the protocol, the Data Protection Act and other regulatory requirements as appropriate.

I agree to ensure that the confidential information contained in this document will not be used for any other purpose other than the evaluation or conduct of the clinical investigation without the prior written consent of the Sponsor

I also confirm that I will make the findings of the trial publicly available through publication or other dissemination tools without any unnecessary delay and that an honest accurate and transparent account of the trial will be given; and that any discrepancies and serious breaches of GCP from the trial as planned in this protocol will be explained.

**For and on behalf of the Study Sponsor**	
Name (please print):	Cheuk Fung Wong
Role:	Research Governance and Quality Assurance Manager
Signature:	DocuSigned by: Cheuk Fung Wong E6ED866F416A4F8...
Date:	18-Oct-2023
**Chief Investigator**	
Name (please print):	Ara Darzi
Signature:	DocuSigned by: Ara Darzi 447CB46CAAB7464...
Date:	18-Oct-2023

1 Study and general information

This document was constructed using The Imperial College Research Governance and Integrity Team (RGIT) Protocol Template. Every care has been taken in drafting this protocol, but corrections or amendments may be necessary. These will be circulated to the registered investigators in the trial.

This protocol has regard for the HRA guidance and order of content

1.1 Sponsor

Imperial College London is the study Sponsor and will undertake and enforce those sponsor duties set out in the UK policy Framework for Health and Social Care. Sponsor assumes overall responsibility for the initiation, management, and funding of this study. The Sponsor is responsible for the design of this study, in addition to its conduct, data analysis and interpretation, and subsequent manuscript writing and dissemination of study findings. The Sponsor controls the final decision regarding any of these aspects of the study.

1.2 Funding

Funding - NIHR Artificial Intelligence (AI Award 2020 Phase 3) Competition. Google will not receive grant funding from the AI Award, and will cover the costs of its staff, technology, and infrastructure.

FUNDER(S)	FINANCIAL AND NON FINANCIAL SUPPORT GIVEN
Artificial Intelligence (AI) Award 2020 Competition 2	Financial
Google Health	Google staff costs across engineering, research scientists, user experience researchers, information governance team, security and privacy team<br>Technical infrastructure costs

Research participants will not receive any payments, reimbursement of expenses or any other benefits or incentives for taking part in this research.

Individual researchers will not receive any personal payment over and above normal salary, or any other benefits or incentives, for taking part in this research.

1.3 Trial Registration

This trial has been registered with the ISRCTN, where it is identified as ISRCTN88754382.

1.4 Research Reference Numbers

Public Health England Research Advisory Committee Ref: BSPRAC_0093

1.5 Trial Administration

Please direct all queries to the Trial Manager at IGHI Coordinating centre in the first instance; clinical queries will be passed on to the relevant site clinicals team via the Trial Manager

1.6 Key study contacts

**Coordinating Site (ICL/IGHI)**		
Imperial College London<br>Institute of Global Health Innovation<br>10th Floor, Queen Elizabeth the Queen Mother Wing<br>St Mary's Campus<br>London<br>W2 1NY	Email: aimstrial@imperial.ac.uk	
**ICL/IGHI Team**		
Project Lead	Hutan Ashrafian	Email: h.ashrafian@imperial.ac.uk
Research Manager	Inês Baptista	Email: i.baptista@imperial.ac.uk
Trial Manager	Aminata Sy	Email: a.sy@imperial.ac.uk
Clinical Research Fellow	Rachita Mallaya	Email: r.mallya@imperial.ac.uk
**Chief Investigator**		

Prof Ara Darzi<br>Lead for Research Translation<br>Director Institute of Global Health Innovation<br>10th Floor, Queen Elizabeth the Queen Mother Wing<br>St Mary's Campus<br>London<br>W2 1NY	Email: a.darzi@imperial.ac.uk
**Co-Investigators**	
Hutan Ashrafian	Lead for Imperial College London
Deborah Cunningham	Co-lead for Imperial NHS Trust
Hema Purushothaman	Co-lead for Imperial NHS Trust
Mamatha Reddy	Co-lead for St George's NHS Trust
Lisanne Khoo	Co-lead for St George's NHS Trust
Lucy Warren	Lead for Royal Surrey NHS Foundation Trust
Fiona Gilbert	Co-Investigator and Scientific advisor
Anna Lawrence-Jones	PPI Lead, Imperial College
Shravya Shetty	Google Health engineering lead
Christopher Kelly	Google Health clinical lead
Jeremy Miles	Statistician, Google Health

1.7 Investigators

Name(s) and address(es) of all medical and/or technical department(s) and/or institutions involved in the project, including any data processors and/or collaborators that will process the data:

- Imaging and Diagnostics Team, Google Health
- Department of Surgery, Imperial College London
- Breast Screening Service, Imperial College Healthcare NHS Trust
- Breast Screening Service, St George's University Hospital NHS Foundation Trust
- Department of Medical Physics, Royal Surrey NHS Foundation Trust
- Department of Radiology, Cambridge University

1.8 Protocol contributors

A wide range of disciplines have contributed to protocol development, including breast radiologists, clinical researchers, user experience/human factors researchers, medical physicists, engineering, statisticians, and patient engagement experts:

- **Imperial College London:** Ara Darzi, Hutan Ashrafian, Rachita Mallya, Anna Lawrence-Jones, Aminata Sy
- **Imperial College Healthcare NHS Trust:** Deborah Cunningham and Hema Purushothaman
- **St George's University Hospitals NHS Foundation Trust:** Mamatha Reddy, Lisanne Khoo
- **Royal Surrey NHS Foundation Trust:** Mark Halling-Brown, Lucy Warren, Kenneth C. Young
- **Cambridge University:** Fiona Gilbert
- **Google Health:** Christopher Kelly, Megumi Morigami, Rory Sayres, Shravya Shetty, Yetunde Ibitoye, Sam Fishman, Richard Sidebottom, Marcin Sieniek, Reena Chopra, Jonathan Dixon, Martin Ho, Jeremy Miles

1.9 Summary of Trial

**Summary information type**	Summary details
**Acronym or short title**	AIMS Part C
**Long title of trial**	Feasibility of an artificial intelligence system to improve the quality and efficiency of breast cancer screening
**Version**	2.0
**Date**	09-Aug-2023

DocuSign Envelope ID: 3755EDA5-CDD3-4119-BD41-A6AF70DAAAAC

**ICL ID #**	22SM7705
**ISRCTN #**	ISRCTN#88754382
**IRAS #**	307842
**Study design**	Feasibility/ pilot study
**Setting**	NHS Tertiary care
**Type of participants to be studied**	Women attending breast cancer screening programme during the study periods will be provided with an information leaflet about the research in the post alongside their appointment confirmation letter. The leaflet will clearly outline details of how to opt-out should they wish. All women who do not opt-out of this study or are not on the national opt-out database will be included in the study.
**Ancillary studies**	Public and Patient Involvement and Engagement
**Sponsor**	Imperial College London
**Interventions to be compared**	Study will demonstrate read-only 'silent' integration of the AI system, in a non-interventional manner, at each participating site interfacing with the other technical systems used within the breast screening programme (NBSS and the Trust PACS), explicitly avoiding any potential for AI outputs to influence patient care.
**Primary outcome measure(s)**	• Time taken for the system to return results<br>• Number of cases correctly excluded during eligibility checks, and reasons for exclusion<br>• Number and types of failures, such as model errors, software errors, integration errors, use errors, and hardware errors encountered
**Exploratory outcome measure(s)**	• Accuracy measures including AI recall rate<br>• AI sensitivity and specificity with respect to arbitrated recall decisions<br>• AI sensitivity for biopsy-proven cancer<br>• AI specificity for biopsy or diagnostic imaging-proven benign lesions.

Study duration	Minimum of 4 weeks; maximum of 8 weeks.
**Estimated number of participants to be studied**	Up to ~14000 women
**Participating sites**	Imperial College Healthcare NHS trust, St. George's NHS Foundation Trust, Royal Surrey NHS Foundation trust (non-clinical site)
**Collaborators**	Google Health, Imperial College London, Imperial College Healthcare NHS Trust, St. George's NHS Foundation Trust and Royal Surrey NHS Foundation Trust
**Duration**	2 years
**Funder**	NIHR-NHSx/ACC
**Chief Investigator**	Professor Ara Darzi

1.10 Lay summary

In the UK breast screening programme, two expert readers (radiologists) assess each mammogram (x-ray of the breast), with any disagreements in opinion reviewed by an arbitration panel of two further readers. However, a radiologist workforce crisis threatens the screening programme's long-term sustainability. Google's AI system identified cancer in mammograms with greater accuracy than specialists (Nature, 2020), suggesting potential to: reduce radiologist workload; increase service capacity; improve accuracy and outcomes, and reduce variability; and reduce time to results, improving patient experience. This project brings this initial research towards real world impact.

The objective of this study is to demonstrate feasibility for the technical integration of an artificial intelligence (AI) system into the standard clinical workflow. This is the third part (Part C) of a multi-stage project which overall aims to evaluate the potential for AI-enabled NHS breast screening to increase accuracy, safety, cost-effectiveness, and clinician/patient experience, while demonstrating evidence of clinical and technical feasibility.

Interventional use of the AI system within the health system cannot be commenced before comprehensive assessments of feasibility and safety, alongside modelling of likely clinical, workflow, and economic impacts. This proposal outlines our plans to translate this research towards real world patient impact through a prospective non-interventional feasibility study that will test the AI system running 'silently' within breast screening clinics at two NHS sites. We will design an AI integration strategy for each site and aim to provide the necessary evidence to form the basis for a proposed deployment framework to support progression to future interventional use in a way that delivers measurable benefits to public health.

2 Introduction

The objective of this study is to demonstrate feasibility for the technical integration of an artificial intelligence (AI) system into the standard clinical workflow. This is the third part (Part C) of a multi-stage project which overall aims to evaluate the potential for AI-enabled NHS breast screening to increase accuracy, safety, cost-effectiveness, and clinician/patient experience, while demonstrating evidence of clinical and technical feasibility. Part A aims to evaluate the accuracy, generalisation and fairness of the AI system using a large retrospective cohort, and Part B aims to understand the impact on expert readers of introducing an AI system as a second reader into a double reader workflow, through a large-scale end-to-end study of arbitration performed at two diverse NHS sites.

2.1 Background

1 in 8 women will be diagnosed with breast cancer in their lifetime. Breast screening aims to find cancers early, where treatment is more successful. In the UK, two radiologists assess each mammogram (x-ray of the breast), with disagreements reviewed by a panel. However, a radiologist workforce crisis threatens our screening programme's long term sustainability.

The UK is facing a 44% shortage in radiologists by 2025 (The Royal College of Radiologists 2021), while it is estimated that only 18% of screening hubs have sufficient staff to cope with current double reading requirements on a 3-yearly cycle (Public Health England 2016). If invitations were to be extended to women aged 47 to 73, as being investigated by the AgeX trial, the demands on the service would increase further (Moser et al. 2011). The negative impact of COVID-19 on breast screening in 2020 only adds to the increased burden expected over the coming years as services catch up. Breast Cancer Now estimated that almost 1 million women have missed their breast screen during the pandemic (Breast Cancer Now 2020), and Macmillan's analysis of the rate of recovery suggests it would take 20 months to work through the current backlog if activity was increased to 10% above pre-pandemic levels (Macmillan Cancer Support 2020). In addition, there are calls for the UK to adopt two-yearly screening, mirroring most other European countries, which would be unfeasible given current workforce pressures, without compromising quality. We believe AI-enabled screening can play an important role in future-proofing the UK's Breast Screening Programme.

Google's Mammo-Reader is an AI-powered independent mammography reader product for double-read breast cancer screening workflows. It analyses two-dimensional full field digital mammography (FFDM) to give a normal/abnormal screening determination, and highlights suspicious regions of interest. In a study published in Nature (McKinney et al. 2020), the AI system was able to demonstrate performance close to that of double reading with arbitration (statistically non-inferior) and superior to the first reader. We believe that deploying this technology as a second reader has the potential to: (1) ultimately improve patient outcomes through improved accuracy and reduced variability; (2) modify the reader / radiologist workload mitigating the current workforce challenges and even allow expansion to alternative screening

strategies such as biannual or personalised stratified approaches; and (3) reduce time to results, improving patient experience. In addition, the discrepancy between a radiologist and the AI system is greater than two humans, suggesting that it may be possible to detect a greater number of cancers (higher overall sensitivity), albeit at a cost of greater number of cases for arbitration.

The AI system is intended to be deployed as a second reader within the UK breast screening programme, integrated securely into the National Breast Screening System (NBSS) and clinical imaging PACS. In our primary proposed workflow, a human screen reading expert will read all cases first. Once the first reader has submitted their assessment, and eligibility checks have been completed the AI system's assessment will be made available. The AI system's decision will be compared to the first reader. In the estimated 10% disagreements, these cases will be referred to an arbitration panel for final determination (following existing local site arbitration rules). We believe this workflow promotes safety by ensuring all AI system decisions require independent agreement from human readers to influence a woman's care, and that all disagreements are reviewed by expert arbitration panels.

This mammography product builds upon Google/DeepMind's world-leading expertise in pioneering artificial intelligence technologies, unique experience in scaling multiple products and services to billions of users around the world, and world-leading Cloud infrastructure with significant experience in healthcare applications. Complementing Google's technology platform, Google Health's cross-functional regulatory, information governance, quality and clinical infrastructure provides a unique environment to build safe, secure, robust, reliable, fair, and accurate clinically validated products to achieve patient impact at scale.

The "Mammography AI system API" has been developed under a Quality Management System adhering to CE certification requirements, and is currently ISO 13485 and ISO 27001 compliant. The certification will expire early 2024 after the anticipated study completion date. The studies described in this application will use the AI system that will be submitted for regulatory approval. The integration pipeline that connects the AI system to the hospital systems via the "Royal Surrey SMART box" and the "Cloud-based Mammo-Client" software has not been developed under a Quality Management System and will not be CE certified. The Google Cloud infrastructure (on which the Mammo-Client software will run) is ISO 27001 compliant.

2.2 Rationale for current study

Interventional use of the AI system within the health system cannot be commenced before comprehensive assessments of feasibility and safety, alongside modelling of likely clinical, workflow, and economic impacts. This proposal outlines our plans to translate this research towards real world patient impact through a prospective non-interventional feasibility study that will test the AI system running 'silently' within breast screening clinics at two NHS sites.

Together with planned retrospective diagnostic accuracy studies (Part A) and large scale consensus panel reader studies (Part B) (REC reference: 22/EM/0038), and this prospective observational feasibility study (Part C), we will design an AI integration strategy for each site, and

aim to provide the necessary evidence to form the basis for a proposed deployment framework to support progression to future interventional use in a way that delivers measurable benefits to public health.

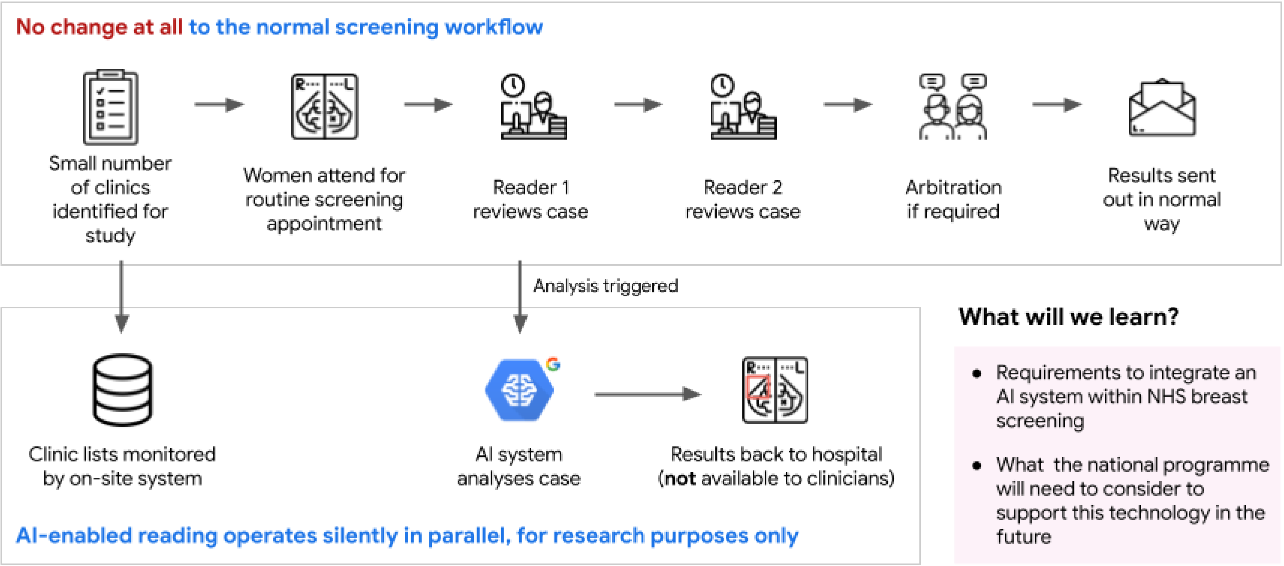

3 Study objectives

3.1 Primary objectives

- Demonstrate read-only 'silent' integration of the AI system at each site (interfacing with NBSS and the Trust PACS), explicitly avoiding any potential for AI outputs to influence patient care. The AI system will have no effect on patient outcomes as part of this study.
- To test the ability for the integrated system to correctly perform eligibility checks and provide AI results only for women who are eligible.

3.2 Secondary objectives

- Build an understanding of the current screening workflows at each site using human factors methodologies, and develop appropriate local integration strategies for future implementation of AI in an interventional setting
- Summarise thematic findings, including experiences and learnings from each site
- Perform detailed analyses of any failures and identify mitigation strategies

4 Selection of Sites and Investigators

In compliance with the principles of GCP, all institutions participating in the trial will complete a delegation log and forward this to ICL/IGHI. Each person working on the AIMS trial must sign off a section of this log indicating their responsibilities. ICL/IGHI must be notified of any changes to trial personnel and/or their responsibilities and an updated delegation log needs to be sent in to ICL/IGHI. An up-to-date copy of this log must be stored in the Investigator Site file at the institution and at ICL/IGHI Trial Master File (TMF).

Following substantial amendments, sites will be notified of relevant documents and training required and updated trial documentation will be sent to sites.

4.1 Principal investigator's qualifications & agreements

1. The investigator(s) should be qualified by education, training, and experience to assume responsibility for the proper conduct of the trial at their site and should provide evidence of such qualifications through an up-to-date curriculum vitae and/or other relevant documentation requested by the Sponsor, the REC, the IRB, and/or the regulatory authority(ies).
2. The investigator should be thoroughly familiar with the appropriate use of the AI system (if appropriate, as described in the protocol, and in other information sources provided by the Sponsor.
3. The investigator should be aware of, and should comply with, the principles of GCP and the applicable regulatory requirements. A record of GCP training should be accessible for all investigators.
4. The investigator/site should permit monitoring and auditing by the Sponsor, and inspection by the appropriate regulatory authority(ies).
5. The investigator is responsible for supervising any individual or party to whom the investigator delegates trial-related duties and functions conducted at the trial site.
6. If the investigator/institution retains the services of any individual or party to perform trial-related duties and functions, the investigator/institution should ensure this individual or party is qualified to perform those trial-related duties and functions and should implement procedures to ensure the integrity of the trial-related duties and functions performed and any data generated."
7. The investigator should maintain a delegation log of appropriately qualified persons to whom the investigator has delegated significant trial-related duties.
8. The investigator should sign an investigator statement, which verifies that the site is willing and able to comply with the requirements of the trial.

4.2 Adequate resources

1. The investigator should have sufficient time to properly conduct and complete the trial within the agreed trial period.
2. The investigator should have available an adequate number of qualified staff and adequate facilities for the foreseen duration of the trial to conduct the trial properly and safely.
3. The investigator should ensure that all persons assisting with the trial are adequately informed about the protocol, and their trial-related duties and functions.
4. The site should have sufficient data management resources to allow prompt data return/transfer to the trial management research

4.3 Required trial documentation

Summary of the required trial documentation for participating sites

Trial Documentation	Timing
Signed Clinical Research Agreement between Trust and Sponsor (or Variation if applicable)	Before site participation
Confirmation of capacity and capability	Before site participation
Trial personnel contact details	Before site participation
Signed Investigator Statement	Before site participation
Signature list & delegation of responsibilities	Before site participation
Site initiation training	Before site participation
Training log	Before sponsor greenlight to commence trial

4.4 Approval and activation

Site training will be performed prior to the activation of the site and will include all processes for the trial including but not limited to protocol training, data management procedures and frequency and expectations for any monitoring visits. A log of attendees will be kept in the TMF as a record of participants present at all types of training events.

Before a site can open to recruitment, formal Sponsor Site Green light will be completed in order to document that the site has met all the requirements to participate in the trial. Written confirmation of site activation will be sent to the PI.

5 Participant entry

5.1 Inclusion criteria

- Women undergoing routine breast cancer screening (age 50–70), as part of the national breast screening programme at Imperial College Healthcare NHS Trust and St George's University Hospital NHS Foundation Trust between the study dates.
- Mammography images acquired using Hologic/Lorad, Siemens, or GE devices.

5.2 Exclusion criteria

- Women that opt-out of this study.
- Women who have registered with the NHS national data opt-out.

6 Assessment and follow-up

There will be no assessments and follow up for this trial. The trial does not involve clinical assessments.

7 Study design and setting

The feasibility study is intended to add no additional burden to women attending for routine screening, and no additional time commitment for screening centre staff. No additional procedures, imaging, or study data will be collected from participants above and beyond routine clinical data.

Following appropriate ethical, information governance, data security, and other local site approvals, technical preparation work will begin at two sites: Imperial College Healthcare NHS Trust and St George's University Hospitals NHS Foundation Trust. Prior to the commencement of the study, each Trust will work with members of the Google Health team to qualify the integration (see 8.3).

Following successful technical integration, the system will be applied to all screening clinics at each site for the study. This study will demonstrate potential for the AI system's integration within the end-to-end double-read screening workflow through the following steps:

- AI system's on-premises client synchronisation with NBSS clinic list
- Ability to identify completed routine screening clinics in real-time ready for processing
- Automatic pseudonymisation of cases and submission to the Google AI system API for analysis
- Ability to write results from API to Cloud-hosted Web PACS UI for researchers to view results using pseudonymous identifiers.

- Ability to return results from API to on-premises client and link with original identifiable record on a separate Trust-hosted research server or research PACS system. Results will explicitly not be viewable by staff members providing standard clinical care.
- Ability to link results to the same original record for women who have more than one screening visit due to a technical recall during the study period.
- Enable the viewing of the results by the local research team once the first human reader has submitted their opinion onto NBSS, demonstrating that the AI system respects the order of analysis.

Clinics will run routinely with no change to existing practices. Following completion of the clinic, the system will automatically analyse cases (excluding patients that have opted-out) and provide an output to the local research team once the first human read is entered into NBSS.

7.1 Integration with hospital IT systems for automated metadata and image collection via Royal Surrey SMART box

The Trust-hosted Royal Surrey Secure Medical image Anonymisation for Research Trials (SMART) box is a piece of software developed by the Royal Surrey NHS Foundation Trust team that is already installed and available at the NHS sites to collect data from the breast screening program, and will be used for the purpose of this study. The SMART box automatically queries NBSS to identify clients involved in a study and subsequently pulls those clients images from PACS and carries out the de-identification process to pseudonymise the clinical data and imaging data used for the study.

The staff setting up the SMART box are named data managers with letters of access giving specific approval for access at the clinical site for this purpose describing the access to patient data required by the data managers. The data managers are full time NHS employees with a duty of confidentiality as regards patient information. The data managers will require access to Patient Identifiable Data (PID) during the setup and any maintenance periods. As far as possible the pseudonymisation process is done in an automated way which avoids the need for the data managers to see any patient identifiable data (PID). Once the automated systems are working, access to PID by the data managers is not necessary. The NHS numbers of the patients whose images are collected are automatically inserted into a lookup database residing on the research server at each main clinical site. Once de-identified and transferred, the original unanonymised images and data are deleted from the research server.

The mapping table between the study pseudonymous identifiers and the hospital identifiers will be stored in on-premises at the Trust. The pseudonyms will be stored in plain text and the other values will be encrypted with envelope encryption via a key management system that is under the control of the Trust. Data will be automatically pseudonymised by the Royal Surrey NHS Foundation Trust SMART box at the point of collection using DICOM 142 Supplement compliance tools. This Supplement is a standard for de-identification of data in DICOM files. Pseudonym lookup tables will be maintained on a secure research server at the NHS site, and access will be restricted to staff involved in the research study or data managers with specific approval to access

the patient data for the purpose of pseudonymisation. The data managers are trained in information governance policies of the host Trust and will have NHS letters of access permitting the level of access required for the pseudonymisation process. Clinical staff will not have access to the encrypted pseudonym lookup tables.

Cases for inclusion in the feasibility study are identified through querying the screening clinics recorded on the NBSS database at each local site following clinic completion. This process is automated and does not need any human intervention from the data managers. The NBSS connection is also used to collect the necessary clinical information about the case, and is recorded against the pseudonymous identifier. All fields recorded from NBSS are numerical/categorical, and no free text fields will be included.

The associated images are queried and retrieved from the site’s mammography PACS. The images are then pseudonymised and all identifiable DICOM tags are stripped or replaced with pseudonyms where necessary, following historical (REC approved) practices of the team at the Royal Surrey NHS Foundation Trust. This practice implements the Basic Application Level Confidentiality Profile with the Retain Patient Characteristics Option (to preserve the patient age in years) as per the DICOM PS3.15 2021e - Security and System Management Profiles (also referred to as DICOM Supplement 142). Further details of the de-identification process of DICOM images is given in Appendix 1.

The pseudonymised images are transmitted to the pseudonymised data Cloud store, hosted in the Google Cloud Platform environment, and then submitted to the AI system for analysis against the pseudonymous identifier. The AI result is then returned back to the pseudonymised intermediate data store and will also be visible in the Web PACS UI (see 5.2).

The Study implementation team are working with the NBSS IT System vendors on enhancements to better support AI systems in the breast screening workflow, for example to manage patient opt-in/out, to allow AI Read results to be recorded into NBSS, and eventually we hope to have NBSS orchestrate the whole AI read workflow. However the feasibility study described herein has been designed to be non-dependent on those enhancements by:

1. Managing patient opt in/out out-of-band to the NBSS system
2. By being observational in nature, no write back is required
3. Integrating with existing read-only interfaces designed to support programme performance reporting (so-called Crystal Reports interface), allows the integration to "bolt onto" the unmodified NBSS for the purpose of a limited scope study.

The secondary outputs of the study, around user-experience research into an idealised integration, will touch more on these future integration points.

7.2 AI system

The AI System is "cloud first", hosted in Google Cloud Platform environment (https://cloud.google.com/). This allows the advanced AI algorithm to be hosted on specialised

DocuSign Envelope ID: 3755EDA5-CDD3-4119-BD41-A6AF70DAAAAC

and well controlled hardware, relieves the need for customer hospitals to have to procure, install and maintain such hardware, and allows centralised monitoring and control of the performance of the AI for overall safety purposes.

The AI system requires a pseudonymous patient_id and case_id for each request; this maps to SxNumber and EpisodeRecordId/AccessionNumber in NBSS, respectively. Each case included in this study will use these two pseudonymous IDs to identify the case.

In order to perform the AI Read, the Cloud-based Mammo-Client will upload images to the Google Health pseudonymised Cloud store, use the Google Health "Mammo-Reader" API to perform the AI Read, and then retrieve the results and output images.

At the end of the study, the data held in the pseudonymised Cloud data store will be anonymised by deletion of the pseudonym lookup table by the Trust. The anonymised dataset will be transferred to both Imperial College London and Google Health research teams for the study analysis.

7.3 Web PACS UI

As the integration will not write back to the Trust hospital systems used in the breast screening program, the feasibility of viewing the results will be demonstrated with the use of Web PACS UI, a Cloud-hosted user-interface, to allow the user to query the database, including which clinics have been assessed, which cases require arbitration, which cases the AI result was withheld, and visibility of the AI outputs. All cases are connected to a study-specific pseudonymous identifier. The pseudonym lookup table will be available at the Trust site to specific members of the research team, who will be able to inspect identifiable information in NBSS and the screening PACS for participants by searching their pseudonymous identifier/NHS number in the lookup table. The Web PACS UI or pseudonym lookup table will not be viewable by staff members providing standard clinical care.

The research team may access and view the results, including the Trust's research radiographer, radiologists, named IT support engineers (either from RSNFT who have letters of access or the Trust, depending on local requirements), and the study coordinator. The AI outputs will be reviewed regularly to ensure all eligible cases have been read for each clinic session.

7.4 Research-only Trust hosted DICOM store

As a proof-of-concept, some cases will be returned with the AI output to Imperial College Healthcare NHS Trust via the RSNFT SMART box where final results will be written to a research-only DICOM store and stored in an identifiable format. The AI result will only be viewable by designated members of the research team after the first human reader (R1) has provided a read. Identifiable information will only be stored on the research DICOM store and will not be viewable by staff members providing standard clinical care.

DocuSign Envelope ID: 3755EDA5-CDD3-4119-BD41-A6AF70DAAAAC

The research team may access and view the results, including the Trust's research radiographer, radiologists, named IT support engineers (either from RSNFT who have letters of access or the Trust, depending on local requirements), and the study coordinator. The AI outputs will be reviewed to ensure all eligible cases have been read for each clinic session.

7.5 Data flow diagram

The data management process is outlined in the figure below.

Screening cases that meet the inclusion criteria for the study are identified by the Trust-hosted Royal Surrey SMART box, which retrieves the mammography images and associated metadata (Step 1). These are pseudonymised by the SMART box (Step 2), before the de-identified images and metadata are submitted to a Cloud-based pseudonymised intermediate data store (Step 3). The data is then submitted to the Cloud-based Client developed by Google Health (Step 4), which applies the eligibility criteria and moves the images to a pseudonymised Cloud store for analysis by the AI system (Step 5). The AI result is returned and is viewable in the Cloud-hosted Web PACS UI once the first human reader (R1) has completed their read (Step 6). The Trust's research team will use the Web PACS UI and the study-specific pseudonym look up table (stored only at the Trust site) to review cases (Step 7). At Imperial College Healthcare Trust only, AI results will be returned to a Trust-hosted research-only DICOM store and re-identified for designated members of the research team to review cases (Step 8). Clinical and imaging data from the query will not be stored by the AI system API. At the end of the study, an anonymised dataset including all images and metadata (including diagnostic and assessment data collected within the 3 month period after the woman has attended screening) will be exported to Google Health and onwards to Imperial College London for study analysis by the research team and archival purposes (Step 9).

DocuSign Envelope ID: 3755EDA5-CDD3-4119-BD41-A6AF70DAAAAC

Imperial College London INSTITUTE OF GLOBAL HEALTH INNOVATION

Google Health

NHS St George's University Hospitals NHS Foundation Trust

NHS Royal Surrey NHS Foundation Trust

NHS Imperial College Healthcare NHS Trust

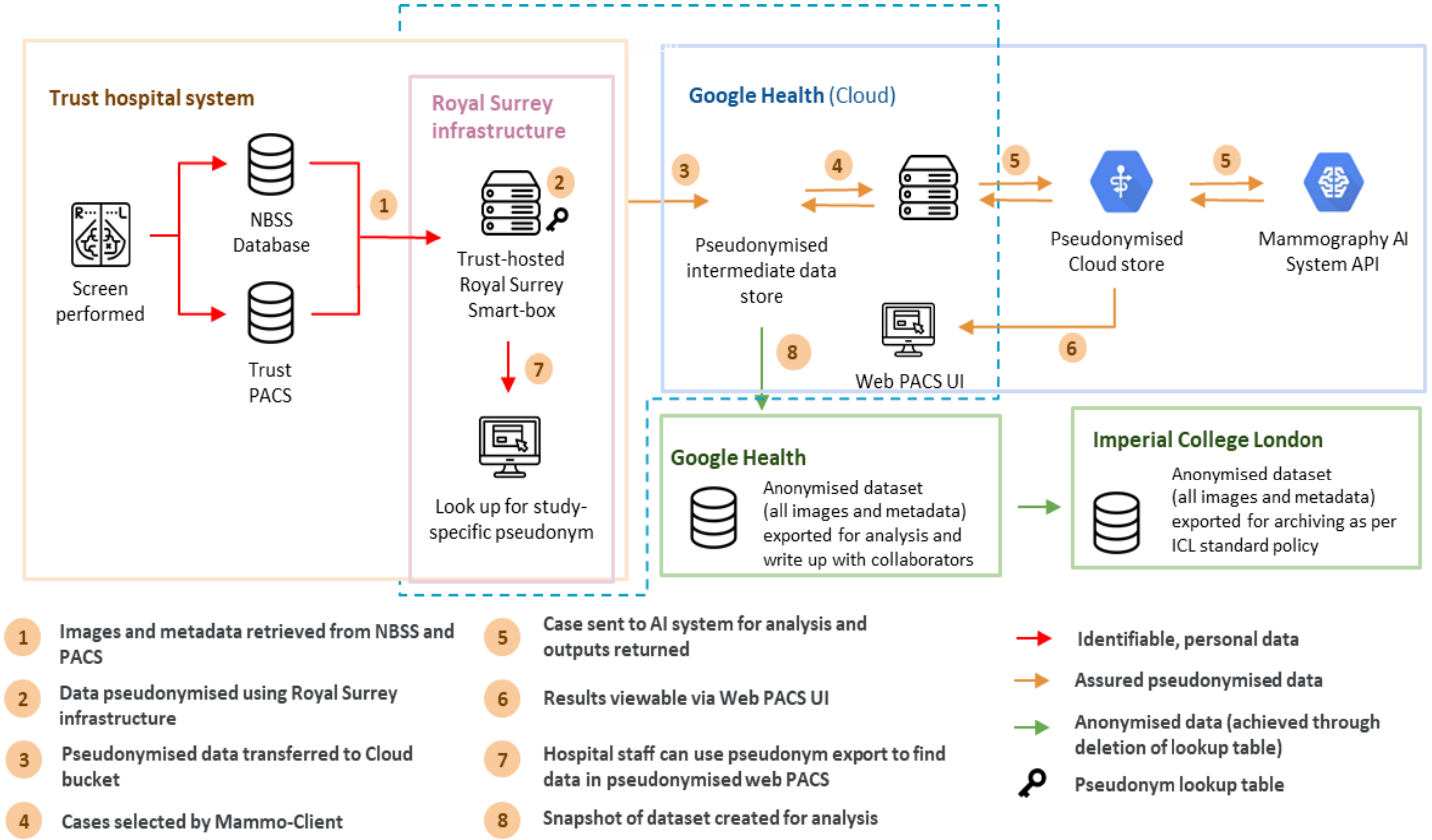

7.6 AI-system eligibility criteria

During this study, we will test the ability for the Integrated Client to correctly apply the eligibility checks and reject cases that should not be read by the AI system. The following criteria apply for a case to be deemed eligible to be read by the AI.

<u>Eligible</u>

- Women undergoing routine breast cancer screening (age 50-70), as part of the national breast screening programme
- Mammography images acquired using Hologic/Lorad, Siemens, or GE devices.

<u>Ineligible</u>

- Women attending an assessment clinic or symptomatic clinic
- Women undergoing annual screening due to:
    - High risk (lifetime risk >30% - e.g. faulty BRCA1, BRCA2, TP53)
    - Moderate risk (lifetime risk 17-30%)
    - Personal stratified follow up (e.g. indeterminate B3 lesions)
- Presence of breast implants

- Screens with incomplete (<4 standard screening views - e.g. due to abandoned screen)
- Poor diagnostic quality imaging (which would be repeated)
- Non-standard acquisitions beyond the routine 4 screening views

The data to check for eligibility will be provided by NBSS (e.g. screening procedure lists, presence of implants, technical recalls) and image DICOM labels (e.g. number of images and views acquired during visit). Integrated Client will read this data to ensure AI results are only available for eligible cases and withheld for ineligible cases.

7.7 Study sites

This study will run at two NHS screening organisations, which each comprise a number of screening sites as described below. Study information materials include information leaflets and posters will be displayed at each of the screening sites. The screening sites will not be performing study activities and will only provide routine clinical care.

7.7.1 St George's University Hospitals NHS Foundation Trust

The South West London Breast Screening Service (SWLBSS) has an eligible population of approximately 165,000 women. The service is provided by the St George's University Hospitals NHS Foundation Trust, and is commissioned by NHS England (London). The programme operates from a base at St George's Hospital and has 6 additional static screening sites across south west London. The service has fully implemented digital mammography, and undertakes high risk screening. In the 3 year period from April 2014 to March 2017, the annual uptake rate of the service was 61.6%, 65.7% and 65.4% respectively (national standard is ≥ 70%). The rate of technical recalls is approximately 0.2%. The pathology service to the SWLBSS is provided by the South West London Pathology Service, based at St George's Hospital. Screen-detected cases are treated at a number of surgical units across the region.

Number of clinics / sites and location: 7 static screening sites

- Base site at the Rose Centre, St George's Hospital, Perimeter Road, London, SW17 0QT
- Further sites at:
  - Teddington Memorial Hospital, Teddington, TW11 0JL
  - Surbiton Health Centre, Surbiton, KT6 6EZ
  - Edridge Road Community Health Centre, Croydon, CR9 1PJ
  - Robin Hood Lane Health Centre, Sutton, SM1 2RJ
  - Queen Mary's Hospital, Roehampton, SW15 5PN
  - Purley Memorial Hospital, Purley, CR8 2LY

Radiographers: 2 radiographers at each peripheral site. At the base site, there are 2 screening mammography machines and therefore up to 4 radiographers.

Volume of women screened per week: Approximately 1,000

Mammography equipment used: Hologic

Screening PACS: Philips PACS, although as part of the South West London PACS collaborative, there are plans to change the PACS supplier in due course.

Arbitration method: All recalls arbitrated

7.7.2 Imperial College Healthcare NHS Trust

The West of London breast screening service (WoLBSS) is provided by Imperial College Healthcare NHS Trust, and serves an eligible population of approximately 172,000 women aged 50–70. The service operates from Charing Cross Hospital, London. The service undertakes digital mammography and provides screening at 5 static sites. In the 3 year period from April 2013 to March 2016, the annual uptake rate of the service was 61.47%, 61.25% and 60.75% respectively (national standard is ≥ 70%). The rate of technical recalls is approximately 0.5%.

Number of clinics and location: 5 static screening clinics

- Charing Cross Hospital (Breast Screening Unit, First Floor, West Wing, Charing Cross Hospital, Fulham Palace Road, London, W6 8RF)
- St Mary's Hospital (Breast Screening Unit, Third Floor, Queen Elizabeth Queen Mother (QEQM) Building, St Mary's Hospital, Praed Street, London, W2 1NY)
- Hanwell Medical Centre (Breast Screening Unit, Hanwell Medical Centre, 20 Church Road, London, W7 1DR)
- Heart of Hounslow Centre for Health (Breast Screening Unit, Heart of Hounslow Centre for Health, 92 Bath Road, Hounslow, TW3 3EL)
- Uxbridge Health Centre (Breast Screening Unit, Room 101, Uxbridge Health Centre, George Street, Uxbridge, Middlesex, UB8 1UB)

Volume of women screened per week: Approximately 750

Mammography equipment used: Hologic

Screening PACS: NovaPACS

Arbitration method: Only discordant recalls arbitrated

7.8 Pilot of technical integration

To test that the system is correctly integrated into the real clinical workflow, a pilot of the system will run over a single clinic day at each site using prospectively collected patient data, followed by a review period. The ability to correctly withhold cases that do not meet the eligibility criteria and the ability to return an output from the AI and store the results in the research database will be verified. Clinical outcomes and performance of the AI in recalling cancers correctly will not be verified. If any errors are observed during the pilot, a further pilot test may be run. A maximum of 4 pilot tests will be performed prior to starting the main feasibility study. The feasibility study will run 4 weeks after the first pilot test has been initiated.

7.9 Main feasibility study

The feasibility study will run for a minimum of 4 weeks and a maximum of 8 weeks at all routine breast screening clinics at each NHS site. This study period was chosen to test the ability for the system to run continuously for a sufficient period of time, and identify examples of all eligibility/ineligibility criteria. One of the rarest types of ineligible cases are 'technical recalls' where the screening images are of poor diagnostic quality and require a repeat visit. We will test the ability of the system to link results to the same original record for patients who have more than one screening visit due to 'technical recall' during the study period. The study will run for a minimum of 4 weeks, and will continue (to a maximum of 8 weeks) until 2 examples of a technical recall who return for their second visit have been identified. The rate of technical recalls is approximately 0.5% at Imperial and 0.2% at St. George's. At each site, women with a technical recall are normally seen for their second visit to repeat the mammography imaging within 2–4 weeks after the read has been completed, and we therefore anticipate that 2 examples will be identified within 4 weeks.

Over this time, it is expected that 1000 patients per week will be screened at St George's and 750 patients per week will be screened at Imperial (1750 patients per week overall). The study will run at each of the two sites sequentially. This provides the opportunity to review findings from the first site prior to running the study at the second site.

All patients whose images are read by the AI will be further followed up for a period of 3 months. This will enable collection of data such as arbitration outcomes and assessment outcomes should the patient be recalled for further investigation. Diagnosis outcomes will provide an estimate of the sensitivity and specificity of the AI system for patients that were recalled to the clinic. This data will be collected by NBSS and linked to the pseudonymised patient record on the Trust's research server.

7.10 End of study definition

The study at each site will end at the conclusion of data collection, defined as 3 months after the last woman has been screened and read by the AI system.

7.11 Study data collected

Imaging collected

- 4 Full-Field Digital Mammography (FFDM) DICOM images (pseudonymised)

Metadata collected

The output data will only include numerical/categorical data types and will not include free text.

Description	Variable name	Data type	Definition
Patient ID	study_id	Ascending integer	Study pseudonymous unique patient identifier
Case ID	case_id	Ascending integer	Study pseudonymous case identifier
**Metadata from NBSS and PACS**			
Age	age	Float	Age of patient at time of initial screen (values 50–70)
Sex	sex	Text, categorical	Patient sex at birth<br>“male”<br>“female”
Ethnicity	ethnicity	Text, categorical	"A" = White - British<br>"B" = White - Irish<br>"C" = White - Any other White background<br>"D" = Mixed - White and Black Caribbean<br>"E" = Mixed - White and Black African<br>"F" = Mixed - White and Asian<br>"G" = Mixed - Any other mixed background<br>"H" = Asian or Asian British - Indian<br>"J" = Asian or Asian British - Pakistani<br>"K" = Asian or Asian British - Bangladeshi<br>"L" = Asian or Asian British - Any other Asian background<br>"M" = Black or Black British - Caribbean<br>"N" = Black or Black British - African<br>"P" = Black or Black British - Any other Black background<br>"R" = Other Ethnic Groups - Chinese<br>"S" = Other Ethnic Groups - Any other ethnic group<br>"Z" = Not stated<br>As per https://datadictionary.nhs.uk/data_elements/ethnic_category.html

	breast_implant	Binary	1 = Breast implants present (case should be excluded)<br>0 = No breast implants
Manufacturer	device_manufacturer	Text, categorical	"GE" = GE Healthcare<br>"Hologic" = Hologic / Lorad<br>"Siemens" = Siemens
Model	device_model	Text	Free text entry for mammographic device used, as described exactly in DICOM header "ManufacturerModelName"
Case type	case_type	Text, categorical	"negative" = no cancer detected at screen, subsequent screen at least 31 months later also negative<br>"benign" = lesion suspicious for cancer at screen, confirmed negative by biopsy or diagnostic imaging, subsequent screen at least 31 months later also negative<br>"cancer" = cancer diagnosed within 39 months of initial screening mammography, confirmed through follow-up core biopsy (not fine needle aspiration)
Screen-detected or interval cancer	screen_interval	Text, categorical	"screen" = Screen detected (i.e. cancer identified on initial mammogram)<br>"interval" = Interval cancer (i.e. presented symptomatically in the interval following this mammogram that was interpreted at the time as negative or benign)<br>"na" = Not applicable (not a cancer case)
Initial screening mammogram date	initial_mammogram_date	Date	Date of the initial routine screening mammogram
Initial mammogram quality acceptable	initial_mammogram_quality	Binary	Initial mammograms of acceptable diagnostic quality standard for screening, with none of the following deficits: inadequate diagnostic quality status such as insufficient anatomical coverage of screening mammograms, inadequate positioning, inadequate compression, incorrect exposure, artefacts obscuring image, image blurring<br>1 = Yes - acceptable quality<br>0 = No - unacceptable quality
Initial mammogram lesion size	initial_mammogram_lesion_size	Text, categorical	Size in mm
Initial mammogram lesion morphology	initial_mammogram_lesion_morphology	Text, categorical	"na" = N/A<br>"IR" = irregular<br>"LB" = lobulated

			"SM" = smooth<br>"SP" = spiculated
Initial mammogram breast density	initial_mammogram_breast_density	Text, categorical	"A" = Class A (Fatty)<br>"B" = Class B (Scattered fibroglandular density)<br>"C" = Class C (Heterogeneously dense)<br>"D" = Class D (Extremely Dense)
Reader 1 decision	initial_mammogram_r1	Text, categorical	"EC" = For Early Recall for Clinic<br>"ES" = For Early Recall for Screening<br>"FN" = For Fine Needle Aspiration<br>"FP" = For Follow-up (Post-treatment)<br>"FV" = For Further X-ray views<br>"IP" = For Inpatient biopsy<br>"MT" = For Medical Treatment<br>"NA" = No Action from this procedure<br>"RC" = For Review in clinic<br>"RF" = For referral to consultant/GP<br>"RR" = Routine recall for screening<br>"ST" = For Surgical Treatment<br>"TR" = For Repeat Film (technical)<br>"WB" = For Wide Bore Needle
Reader 2 decision	initial_mammogram_r2	Text, categorical	"EC" = For Early Recall for Clinic<br>"ES" = For Early Recall for Screening<br>"FN" = For Fine Needle Aspiration<br>"FP" = For Follow-up (Post-treatment)<br>"FV" = For Further X-ray views<br>"IP" = For Inpatient biopsy<br>"MT" = For Medical Treatment<br>"NA" = No Action from this procedure<br>"RC" = For Review in clinic<br>"RF" = For referral to consultant/GP<br>"RR" = Routine recall for screening<br>"ST" = For Surgical Treatment<br>"TR" = For Repeat Film (technical)<br>"WB" = For Wide Bore Needle
Arbitration decision	initial_mammogram_r3	Text, categorical	"EC" = For Early Recall for Clinic<br>"ES" = For Early Recall for Screening<br>"FN" = For Fine Needle Aspiration<br>"FP" = For Follow-up (Post-treatment)<br>"FV" = For Further X-ray views<br>"IP" = For Inpatient biopsy<br>"MT" = For Medical Treatment<br>"NA" = No Action from this procedure<br>"RC" = For Review in clinic<br>"RF" = For referral to consultant/GP<br>"RR" = Routine recall for screening<br>"ST" = For Surgical Treatment<br>"TR" = For Repeat Film (technical)<br>"WB" = For Wide Bore Needle

Date of follow up mammogram	followup_mammogram_date	Date	In negative and benign cases (initial_mammogram_report), follow up mammogram if performed. Must be at least 21 months after initial_mammogram_date
Radiology report for follow up screening mammogram	followup_mammogram_report	Text, categorical	In negative and benign cases (initial_mammogram_report), radiology conclusion of screening mammogram - values:<br>"negative"<br>"benign"<br>"cancer" = Cancer (Malignant)
Biopsy performed	biopsy_performed	Binary	0 = No<br>1 = Yes
Biopsy results	biopsy_result	Text, categorical	"negative" = No abnormality found<br>"benign"<br>"dcis" = Ductal carcinoma in situ<br>"invasive" = Invasive carcinoma<br>"na" = Biopsy not performed
Biopsy result detail	biopsy_pathology_detail	Text, categorical	Normal<br>"negative" = No cancer<br><br>Benign lesions:<br>"BCC" = Columnar cell change<br>"BCR" = Complex sclerosing lesion/radial scar<br>"BDE" = Periductal mastitis/duct ectasia<br>"BFA" = Fibroadenoma<br>"BFC" = Fibrocystic change<br>"BPM" = Multiple papilloma<br>"BPS" = Solitary papilloma<br>"BSA" = Sclerosing adenosis<br>"BSC" = Solitary cyst<br>"BXX" = Other benign lesion<br><br>Non-invasive lesions:<br>"ND" = Ductal<br>"NIL" = Lobular<br>"NIP" = Paget's<br><br>Invasive lesions:<br>"IDC" = Invasive ductal carcinoma<br>"ILC" = Invasive lobular carcinoma<br>"IMC" = INvasive medullary carcinoma<br>"IMD" = Invasive mixed<br>"IMU" = Invasive mucinous carcinoma<br>"ITC" = Invasive tubular carcinoma<br>"IMX" = Other malignant tumour

Invasive tumour grade	diagnosis_tumour_grade_invasive	Text, categorical	"grade1" = Grade 1<br>"grade2" = Grade 2<br>"grade3" = Grade 3<br>"unknown" = Unknown<br>"na" = Not applicable
Non-invasive tumour grade	diagnosis_tumour_grade_noninvasive	Text, categorical	"low" = Low grade<br>"medium" = Medium grade<br>"high" = High grade<br>"unknown" = Unknown<br>"na" = Not applicable
**Inclusion/Exclusion screening fields**			
Sex is female	incl_criteria_femalesex	Binary	1 = Female, include<br>0 = Not female, Do not include
Age is 50–70	incl_criteria_age	Binary	1 = 50–70 years old inclusive<br>0 = <50 or >70 years old
Correct manufacturer	incl_criteria_manufacturer	Binary	Acquired with devices from three manufacturers; Hologic/Lorad, Siemens, and GE<br>1 = Yes; include<br>0 = No; do not include
Case meets inclusion / exclusion criteria	case_included	Binary	1 = Case meets all inclusion / exclusion criteria<br>0 = Case does not meet all inclusion / exclusion criteria
**Eligibility criteria**			
Four views FFDM	incl_criteria_views	Binary	4 views (RCC, LCC, RMLO, LMLO) images of FFDM (Full-Field Digital mammography)<br>1 = Yes; include<br>0 = No; do not include
Breast implant in screening mammogram	excl_criteria_implants	Binary	1 = Exclude as implants present in either breast<br>0 = Do not exclude
Adequate quality of mammogram	excl_criteria_quality	Binary	1 = Exclude as initial mammogram not of diagnostic quality (reasons including: inadequate diagnostic quality status such as insufficient anatomical coverage of screening mammograms, inadequate positioning, inadequate compression, incorrect exposure, artefacts obscuring image, image blurring).<br>0 = Acceptable quality; do not exclude

Screening due to high risk	excl_criteria_high_risk	Binary	1 = Exclude high risk screening (lifetime risk >30% - e.g. faulty BRCA1, BRCA2, TP53)<br>0 = Do not exclude
Screening due to moderate risk	excl_criteria_moderate_risk	Binary	1 = Exclude moderate risk screening (lifetime risk 17-30%)<br>0 = Do not exclude
Screening due to personal stratified follow up	excl_criteria_stratified_followup	Binary	1 = Exclude personalised stratified follow up screening (e.g. indeterminate B3 lesions)<br>0 = Do not exclude
**AI metadata**			
Timestamp of AI analysis started	ai_analysis_date_started	Date	Timestamp of AI analysis started
Timestamp of AI analysis completed	ai_analysis_date_completed	Date	Timestamp of AI analysis completed

7.12 Study outcome measures

7.12.1 Primary outcome measures

- Time taken for the system to return results.
- Number of cases correctly excluded during eligibility checks, and reasons for exclusion.
- Number and types of failures, such as model errors, software errors, integration errors, use errors, and hardware errors encountered.

7.12.2 Exploratory outcome measures

- Accuracy measures including AI recall rate, AI sensitivity and specificity with respect to arbitrated recall decisions, AI sensitivity for biopsy-proven cancer, AI specificity for biopsy or diagnostic imaging-proven benign lesions.

7.13 Statistical analysis

Descriptive statistics will be used to measure the number of cases successfully analysed, the number of failures, and the time taken for the system to return a result. Qualitative analysis will be used to explore and summarise thematic findings including successes and failures encountered during the study.

Exploratory inferential statistics will be conducted to determine accuracy measures, but no conclusions will be made from the results given we will not have adequate numbers for sufficient power, nor longitudinal follow up for accurate ground truth determination.

7.14 Presentation of results

On completion of the study, the data will be analysed and tabulated, and a final study report will be prepared that will be shared internally with the research group and with the NIHR. Results may be presented at scientific conferences and will be published in a peer-reviewed academic journal with open access as soon as possible following the report finalisation. Results will be available on the study website.

8 Consent

Informed consent is not sought because the AI system does not obtain or store identifiable data for this feasibility study. The only processing of patient identifiable data is the de-identification process itself which takes place at the point of collection on a dedicated Trust computer, in an automated manner. Patients whose data may be used during the study will be informed and given the opportunity to opt-out of the research (details below). Approval will be sought from the Confidentiality Advisory Group (CAG) before starting the study.

The focus of this study is demonstrating technical feasibility (rather than any clinical performance metric). For this reason, there will be no change to patient care as a result of inclusion in this study, and all patients will receive the existing high quality screening programme care. Results from the AI system will be blinded to the clinical team and will explicitly not influence patient care.

The research group considered obtaining informed consent for this study in order to enable additional study components such as patient questionnaires. However, given COVID-19 backlogs, reluctance to disrupt patient flow, logistical difficulties with screening vans, and administrative complexity with appointments being made centrally with little flexibility, the research group has opted to avoid the need for informed consent. Through an opt-out (or “active dissent”) model, our study will more closely resemble real world conditions, and include a non-biased and representative population within these real-world NHS settings. This approach was thought to offer greater acceptability to the woman attending for screening.

The options of informed consent versus an opt-out process were presented to our patient and public engagement group in July 2021 during an ethics-themed session. As this study is not interventional, participants in the group favoured an opt-out process to ensure there are no delays during screening to obtain consent and so women are not overwhelmed with information during their screening visit. The group recommended that a synopsis of the study should be provided to women through an information leaflet or a video that includes 1) reassurances around confidentiality and privacy of data, 2) transparency of Google’s involvement in the research 3) the potential benefits of this research, 4) details on how women can opt-out.

To ensure that women attending for screening are fully aware of their centre’s participation in this study, women whose data may be used for the study will be informed through a 2-page information leaflet that will be posted together with their screening visit appointment letter by the London Breast Screening Hub. The leaflet incorporates feedback provided by participants during

a further engagement event held in September 2021. The leaflet includes a clear explanation for how we will respect ICO rules, including information on what we do with the data, where the data is going, how they can enact their data rights, and how they can opt out later on. The 2-page information leaflet has been translated into several languages including Arabic, Gujarati, Hindi, Polish, Punjabi, Tamil, and Urdu, and will be available on the study website and also at the screening clinics. A short video describing the research will be provided as a link to the study website in the information sheet. Detailed information leaflets will be available online, and posters will be displayed at screening sites with information on how to opt-out. Through this comprehensive approach, we aim for all women to be fully informed about the study, and be given every opportunity to decline to participate.

8.1 Opt-out process

Women can opt-out by entering their details into an eOptOut portal (via internet access) or call a study-specific phone number. Opt-outs will be recorded in a dedicated opt-out table. This will ensure that their data is not used for the research. Women will also be able to opt-out at any time within 3 months of their screening visit. If patients opt-out after their data has already been processed by the AI system, the research team will ensure that the returned AI output is removed from the pseudonymised Cloud store and the Trust research server, and their data will not be used for analysis. The research team will confirm with the woman that their dissent has been registered.

If a woman decides that she would like to opt-out, she may visit the AIMS website or go directly to an eOptOut portal set up by RSNFT. The ePortal directs the woman to a URL to complete a form to automatically opt-out the woman from the study. URL details of the eOptOut portal are provided on the participant information leaflet and the poster. The form will have fields for the Name, Date of Birth, NHS number (mandatory) and Screening Site. The woman will receive a PDF certificate confirming opt-out. Details entered on the ePortal will be stored in an encrypted manner. The RSNFT SMART box will send requests to the eOptOut portal (via an API) to ask if a specific hashed NHS number has opted out.

The study-specific phone number will lead to a voicemail inbox, with a prompt asking the patient to leave their name, date of birth, NHS number, the Screening Centre they attend, and their phone number, if they choose to opt out. A research coordinator from one of the Trust sites will monitor the voicemail inbox daily and communicate opt-outs with the research team at each of the Trusts. The research coordinators at each Trust site will register the opt-out on the local SMART box. The woman will receive a phone call back from the research radiographer to confirm that their opt-out has been registered.

In addition, the project will respect the NHS National Data Opt out Programme (NDOP) by performing checks from the Trust research server that holds the pseudonyms.

9 Data management

The RSNFT SMART box running on local hospital systems will perform automatic case identification and pseudonymisation (i.e. patient identifiers are replaced by a pseudonym), before passing the de-identified data to the cloud-based AI system for analysis. Data will be pseudonymised using DICOM 142 supplement compliance tools. This supplement is a standard for de-identification of data in DICOM files.

Pseudonym lookup tables and key material used for re-identification are stored securely at the clinical sites, and will only be accessible to designated members of the Trust's research team or data managers with specific approval to access the patient data for the purpose of pseudonymisation. The data managers will have NHS letters of access permitting the level of access required for the pseudonymisation process.

Each site will have a dedicated research server on which the RSNFT SMART box will be set up. All patient identifiable data will be processed on this computer, which remains within the clinical site. Access to this computer will be restricted to approved data managers with appropriate research passport/letter of access for that site. The amount of contact that the data manager will have to any patient data is kept to a minimum by maintaining an automated system. This means that only minimal contact is required to configure and customise the system for the local site infrastructure, to test/verify that it is working correctly and diagnose any issues if they arise.

The engineering team providing the Cloud service will have no access to patient identifiable information. All staff members at Google Health implementing this project have been trained in information governance policies specific to working with health data.

Local Trust Information Governance teams (CIO, CCIO, Information Governance Lead and Caldicott Guardian) will be closely involved in the set up of the system, working with Google Health Information Governance teams.

Research data collected in the course of this study will not be used for any other purpose other than described in this protocol.

9.1 Data encryption and security

The AI system that analyses the pseudonymised mammography images will run on Google-controlled secure infrastructure within Google data centres. All data will be encrypted in transit to/from the AI system, and at rest. All data and systems will be secured with state-of-the-art encryption and Access Control Lists (ACLs), which are tightly controlled and logged. Both automated and manual review processes are in place around the audit logs. Access to data and systems is restricted to those with explicit permission through ACLs, and only through secure computers. Only those working directly on the project will have access to the project infrastructure.

- **Encryption 'in-transit':** all data is transmitted over secure HTTP, encrypted with Transport Layer Security (TLS).
- **Encryption 'at rest':** All data stored in Google Cloud is encrypted at the storage level using AES256. Google uses several layers of encryption to protect health data. Data for storage is split into chunks, and each chunk is encrypted with a unique data encryption key. These data encryption keys are stored with the data, encrypted with ("wrapped" by) key encryption keys that are exclusively stored and used inside Google's central Key Management Service. Google's Key Management Service is redundant and globally distributed.
- **Access to data:** Data access will require a member of the engineering team to be logged into a corporate computer with two-factor authentication. The engineer must then be added to an access control list which requires verified access and approval. Finally, the engineer will receive a short-lived certificate for access. Every 24 hours the engineer will need to re-authenticate his/her computer.
- **Geolocation of data processing:** Data will be processed both in the UK, countries with an EC adequacy decision, and also the US. As per ICO guidance, data processing in the US will occur under a contract that incorporates standard data protection clauses recognised or issued in accordance with the UK data protection regime. These are known as standard contractual clauses (SCCs). The SCCs contain contractual obligations on both the data exporter and the data importer, and rights for the individuals whose personal data is transferred.

9.2 Integration qualification process

Prior to the commencement of the study, each Trust will work with members of the Google Health team to qualify the integration to test that:

1) The local Trust client can correctly identify cases.
2) The pseudonymisation process operates correctly.
3) Images and requests can be successfully transmitted.
4) The AI system is able to analyse these images.
5) The local trust client correctly receives the results.
6) The AI system results are stored in a way that unauthorised staff, and staff working in the routine clinical workflow cannot access them.
7) The AI system results are accessible by radiologists as needed to support the feasibility study.

The qualification process will involve direct interactions between the IT staff at a Trust along with employees at Google Health to make sure each of the above points are thoroughly tested before the commencement of the study.

9.3 Data storage at study completion

There will be a number of datasets that will be created through our research activities. At the completion of the study, these will be processed as follows:

1. **Identifiable data with associated AI results, stored at each participating Trust**
   This data will be held securely within the jurisdiction of the relevant hospital Trust, with whom responsibility lies for deletion.

2. **Pseudonymised data held by data processors for operation of AI system**
   All pseudonymised data held with the data processors (Royal Surrey NHS Foundation Trust and Google Health) will be deleted after the archiving period under direction of the Data Controller (Imperial College London).

3. **De-identified AI results, clinical metadata, operational information, outcome information**
   An anonymised dataset will be curated with AI results, clinical metadata, operational information (such as time to inference, logs etc), study results and analysis. Anonymised research data generated by the study will be archived after study completion for a period of 10 years, as per Imperial College London policy.

   Data will be stored securely at the Institute of Global Health Innovation at Imperial College London. Access will be granted to the Principal Investigator and their deputies only, with security controlled through two-factor authentication. Google Health will retain anonymised study data for a maximum period of 10 years. The study dataset will be stored in dedicated, encrypted, secure health research storage, with two-factor authentication, and strict access control lists that are limited to researchers directly working on this research study.

10 Oversight and Trial Committees

10.1 Trial Management Group (TMG)

A Trial Management Group (TMG) has been formed comprising: the project lead, collaboration partners, co-investigators, site research personnel, and members of ICL/IGHI Trial Management Team. The TMG will be responsible for the day-to-day running and management of the trial. The TMG will meet by teleconference at least on a monthly basis where possible and in person as needed.

Further details of TMG functioning are provided in the TMG charter (available on request).

10.2 Trial Steering Committee (TSC)

A Trial Steering Committee (TSC) has been formed to provide overall supervision for the trial and provide advice through its independent chair. The TSC consists of representatives from each organisation, alongside patient and public representatives. The TSC will meet regularly, as required by the trial, and at least quarterly to ensure that the study is progressing well, review progress, and prepare relevant reports for Sponsor.

10.3 (Independent) Data Monitoring Committee (I[DMC])

The trial will not consist of an IDMC due to the nature of the trial design.

10.4 Patient and Public Involvement Advisory Groups

The trial has an established Patient and Public Involvement (PPI) group, see **section 9**.

10.5 Role of Study Sponsor

The study is sponsored by Imperial College London with responsibilities defined by a written Research Collaboration agreement with collaborators. Imperial College London will undertake and enforce those sponsor duties set out in the UK policy Framework for Health and Social Care. Delegated responsibilities will also be assigned to the NHS Trusts taking part in this study.

11 Patient and Public Involvement and Engagement (PPIE)

Patient and Public Involvement (PPI) in research is defined by INVOLVE (an advisory group established by the NIHR) as research being carried out 'with' or 'by' members of the public rather than 'to', 'about' or 'for' them. INVOLVE intends 'public' to include patients, potential patients, carers and other users of health and social care services, as well as people from organisations that represent people who use services. In some cases, this may include involvement of a trial's participants in guidance or oversight of a trial. Under the Imperial College Research Ethics Committee process, the PPIE study has been approved **(ICREC reference: 21IC6635)**

11.1 PPIE Strategy

AIMS will have a comprehensive PPI strategy which will involve participants, the public and key community stakeholders throughout the life cycle of the trial. At the core of the strategy will be the commitment to active PPI during the study, building partnerships with participants, the public, local stakeholders to shape decisions about the research. The term 'participant and public' includes former patients, people who use local health care and social services and people who represent those services.

The goal is to gain a broad and diverse range of perspectives rather than achieve representativeness of specific population groups.

The strategy will describe:

- The models of PPIE that will be used in AIMS
- The use of PPI through the research cycle of AIMS
- The framework for assessing the impact of PPI
- The procedures for supporting PPI

11.2 PPI contributors

From project inception in 2016, we have had 2 lay partners with personal experience of breast cancer as part of a committee comprising approximately 10 UK breast screening radiologists and academics. We have recruited 2 additional lay partners to join our steering committee to increase the diversity of voices and in case members cannot attend. Lay partners will be directly involved in decision making, design and dissemination of findings. In addition, they will provide input into infographics and blogs aimed at the general public.

All lay partners will be appropriately trained and have access needs supported. Due to COVID-19, we may have to carry out meetings remotely. To ensure our opportunities are inclusive, we will add a phone number to the workshop advert and advertise through community groups. We will send guidance and pay for any dongles for those without internet access. Members will be paid £25/hour and £5 for any online interaction.

11.3 Protocol design and study setup

PPI contributors are involved in every stage of the initial development, from training data specification and use-case, to evaluation strategy and iteration of experiments. This ensured that our AI system meets the needs of the screening programme, radiologists and patients alike - classifying occurrence of cancer within 3 years (designed around the NHS programme interval), acting as second reader (advised by Public Health England as their most impactful application), and validated in a large representative dataset.

11.4 PPI in the ongoing running of study

Patients have been involved in all our original research. Two lay partners with experience of breast cancer participated in every stage of the initial model development, from training data specification and use-case, to evaluation strategy, iteration of our experiments, and preparing our publication. We will recruit two additional lay partners to the steering committee to expand diversity of input. Lay partners will be involved in decision making, design, and dissemination. We will carry out workshops with wider groups of people that attend mammography screening or have been affected by cancer to discuss ideas, concerns, and expectations for the project. They will

continue to be involved throughout the lifecycle of the trial and will provide input when writing manuscripts for publications.

11.5 PPIE Workshops

11.5.1 Aim

To understand public/patient ideas, concerns and expectations about the use of AI in breast screening mammography.

11.5.2 Methods

We will run workshops with diverse groups of up to 14 participants who have had experience of breast cancer or have previously experienced routine mammography screening. These workshops are co-facilitated by a lay partner. We will discuss patients' ideas, concerns and expectations for the project, which will feed into project design. All lay partners will be appropriately trained and have access needs supported. Due to COVID-19, we may have to carry out meetings remotely. To ensure our opportunities are inclusive, we will add a phone number to the workshop advert and advertise through community groups. We will send guidance and pay for any dongles for those without internet access. Members will be paid £25/hour and £5 for any online interaction.

We have so far run 5 workshops in 2021 and 2 workshops in 2022. During these workshops, participants provided feedback on the design of the feasibility study, including discussion of informed consent versus an opt-out process. Participants were closely involved in designing and critically reviewing the participant information sheet and information video. We will be arranging a series of further workshops in 2022 as the study progresses.

11.6 Reporting and evaluating impact of PPI

We will evaluate the impact of our PPI using the GRIPP2 academic tool and asking for feedback.

An impact assessment form will be used to collate the purpose, implementation and response for each PPI activity throughout the course of the trial. If recommendations from PPI activity are not implemented, the reason why will be documented on the assessment form.

12 Ethical and Regulatory considerations

All regulatory requirements will be met by the sponsor or their delegated authorities. The sites will adhere to their local requirements for information governance and research processes.

DocuSign Envelope ID: 3755EDA5-CDD3-4119-BD41-A6AF70DAAAAC

12.1 Regulatory compliance

The trial will be conducted in compliance with the approved protocol, the Declaration of Helsinki 1996, the principles of Good Clinical Practice (GCP), Commission Clinical Trials Directive 2005/28/EC* with the implementation in national legislation in the UK by Statutory Instrument 2004/1031 and subsequent amendments, the UK Data Protection Act, and the and the National Health Service (NHS) Research Governance Framework for Health and Social Care (RGF).

*Until the Clinical Trials Regulation EU No 536/2014 becomes applicable, the trial will be conducted in accordance with the Clinical Trials Directive as implemented in the UK statutory instrument. When the directive is repealed on the day of entry into application of the Clinical Trial Regulation the trial will work towards implementation of the Regulation (536/2014) following any transition period.

12.2 Protocol compliance

Prospective, planned deviations or waivers to the protocol are not allowed under the UK regulations on Clinical Trials and will not be used.

Accidental protocol deviations must be adequately documented on the relevant forms and reported to the Chief Investigator and Sponsor immediately.

12.3 Site compliance

Participating site will comply with the above and an agreement will be in place between the site and the Sponsor.

The site will inform ICL/IGHI as soon as they are aware of a possible serious breach of compliance, so that the Trials Coordinating Centre can report this breach within the timelines required by the regulatory authorities. For the purposes of this, a 'serious breach' is one that is likely to affect the scientific value of the trial to a significant degree.

12.4 Ethical conduct

The protocol will have a Favourable Opinion from an appropriate Research Ethics Committee, according to national guidelines. Additionally, each site must also obtain management permission for research (Local Confirmation of Capacity and Capability approval or equivalent) before participating in the trial.

The right of the participant to opt-out of taking part in the trial without the stipulated time frame of 3 months after breast screening must be respected.

12.5 Research Ethics Committee review

- The Study Coordination Centre has obtained approval from the Nottingham Research Ethics Committee (REC) and Health Research Authority (HRA) for the trial protocol, and other relevant documents.
- The study must also receive approval from the Confidentiality Advisory Group (CAG) before starting the study.
- The study must also receive confirmation of capacity and capability from each participating NHS Trust before accepting participants into the study or any research activity is carried out. The study will be conducted in accordance with the recommendations for physicians involved in research on human subjects adopted by the 18th World Medical Assembly, Helsinki 1964 and later revisions.
- Substantial amendments that require review by REC will not be implemented until the REC grants a favourable opinion for the trial.
- All correspondence with the REC will be retained in the Trial Master File/Investigator Site File.
- An annual progress report (APR) will be submitted to the REC within 30 days of the anniversary date on which the favourable opinion was given, and annually until the trial is declared ended
- It is the Chief Investigator's responsibility to produce the annual reports as required.
- The Chief Investigator will notify the REC of the end of the study
- If the study is ended prematurely, the Chief Investigator will notify the REC, including the reasons for the premature termination
- Within one year after the end of the trial, the Chief Investigator will submit a final report with the results, including any publications/abstracts, to the REC.

12.6 Peer review

This study is funded by the Artificial Intelligence in Health and Care Award. As part of the application process for this award, the study underwent independent external review by the Accelerated Access Collaborative in partnership with NHSX and the National Institute for Health Research (NIHR), in addition to the Breast Screening Research Advisory Committee (RAC). The protocol underwent further review by the NHS Accelerated Access Collaborative Evaluation Advisory Group (EAG) on 28th February 2022 and the RAC on 29th March 2022. The study has been reviewed by the Imperial College London Research Governance & Integrity (RGIT) peer review service.

12.7 Indemnity

- Given this is a data-only study, with no direct contact with participants, the potential legal liability of the sponsor(s) for harm to participants arising from the management, design, and conduct of the research is felt to be minimal.
- The study only involves sites that are covered by the NHS indemnity scheme.
- Imperial College London holds negligent harm and non-negligent harm insurance policies which apply to this study.
- In addition, the study and protocol will be submitted to each participating site's Research & Development Department for the usual legal approvals.

12.8 Financial and other competing interests

Members of the study group will be required to declare:

- Ownership interests that may be related to products, services, or interventions considered for use in the trial or that may be significantly affected by the trial,
- Commercial ties requiring disclosure include, but are not restricted to, any pharmaceutical, behaviour modification, and/or technology company
- Any non-commercial potential conflicts e.g. professional collaborations that may impact on academic promotion.

A record of declarations will be kept by the Chief Investigator.

12.9 Protocol amendments

- Amendments will be submitted to the Sponsor and the relevant REC using forms provided by the HRA.
- The Sponsor and Chief Investigator will be responsible for the decision to amend the protocol and for deciding whether an amendment is substantial or non-substantial.
- Copies of amendment documentation will be provided to relevant stakeholders including R&D departments at each site.
- A record of changes will be maintained by the Chief Investigator.

13 Quality Assurance and Control

13.1 Risk assessment and management of risk

This study involves contemporaneous analysis of women's screening mammograms. In this study, the recall decision made by the AI system will not be known by the treating clinical teams during the study, and therefore the system will not influence clinical decisions or provision of care.

The research team considered the ethical need to alert the screening clinical team to cases where our research identified a suspicious case for recall that had been assigned a routine recall

decision by the standard screening process. We concluded that feedback should not be provided to the clinical team for the following reasons:

- The observational study is deliberately intended not to change the complex dynamics of a healthcare system, and feeding back discordant results would indirectly make this study interventional.
- By only feeding back suspected false negative cases (without also feeding back false positive cases that may already have been assessed/biopsied), we may influence a higher patient recall rate, potentially increasing morbidity without improving mortality.
- A study that has the potential to influence patient care should obtain informed consent, which the team would prefer to avoid during the period of COVID-19 restrictions.
- While we do have prior evidence of superior diagnostic accuracy of the AI system compared to the average reader in previous cohorts, this does not necessarily imply absence of clinical equipoise regarding impact on overall health.
- While the research consensus panel consists of similar mammography experts to those performing routine cases, it is possible that their decisions are influenced by outputs from the AI system (Haenssle et al. 2020; Kiani et al. 2020), and so results should be treated with caution until careful system evaluation has been performed.

Ensuring the highest level of data security and privacy is a fundamental underlying theme of this project, as described in detail in Section 8. Great care will be taken to minimise any potential risk to patients due to data breach.

13.2 Potential for unintended bias

Google Health is very conscious of the potential for AI systems to propagate biases in healthcare. Blind spots in AI systems can reflect the worst societal biases, with a risk of unintended or unknown accuracies in minority subgroups. As a result, the AI system has been developed through the curation of large training datasets from two UK breast screening sites, encompassing scans of patients thought to closely represent the overall UK breast screening population. We believe that this methodology has minimised the possibility of algorithmic biases to date, and we have explored algorithm performance across many clinical and demographic subgroups.

Despite this, we need to achieve greater confidence about the AI system's performance across various underrepresented subgroups that were not possible due to limited sample size in the original paper. Through the studies outlined in this application, we plan to more rigorously explore the performance of the system in larger, more diverse datasets, in order to ensure that any product created is safe for future deployment at scale.

13.3 Monitoring and audit

The study may be subject to inspection and audit by Imperial College London under their remit as sponsor and other regulatory bodies to ensure adherence to GCP and the UK Policy Framework

for Health and Social Care Research. The Chief Investigator will be responsible for the monitoring of the study.

13.4 Confidentiality

All information collected during the course of the research will be kept strictly confidential, we plan to follow the principles of the UK Data Protection Act.

The Chief Investigator will preserve the confidentiality of participants taking part in the study and is registered under the Data Protection Act.

Data will be pseudonymised for processing by data processors then anonymised prior to transferring to Sponsor for analysis.

Pseudonymised data will be transferred to Royal Surrey NHS Foundation Trust and Google Health. Anonymised data will be transferred to Imperial College London.

14 Expected outcomes of the study, Patient and Public Engagement and Publication and Dissemination of results

Through this project funded by the AI Award, we aim to gather evidence of standalone accuracy from two large scale validations of the AI system (Part A), quantify human factors when being used by clinicians (Part B), and demonstrate feasibility of real world integration into live clinical systems (Part C). We hope this strategy will provide appropriate evidence of feasibility, efficacy and safety for the National Screening Committee to consider a future major modification to the screening programme. This work will also provide evidence for the wider clinical community to understand the impacts of this technology. This project seeks to understand factors that influence public engagement in AI technology, and what may foster better confidence in AI systems.

This study has clear anticipated public health benefits. Results from clinical, workflow and economic analyses will be used to design future interventional studies, and make recommendations about safe future integration of the AI system into routine clinical practice, supporting decision making by NHS England and the National Screening Committee. All results will also be presented at a formally designated Patient and Public Involvement and Engagement (PPIE) group whose interim and final meeting comments will be audited, presented to the steering committee and included in the final presentation of this work.

We plan for this work to be published in peer-reviewed academic journals with open source access as soon as possible following completion, following the forthcoming STARD-AI guidelines for diagnostic accuracy in AI studies (being led by members of this project (Sounderajah et al. 2020)). It is intended that the study will be published as a multicentre study. Findings will also be disseminated through infographics and blogs aimed at the general public, with the assistance of our patient and public representatives.

The preparation of a manuscript for publication in a peer-reviewed professional journal or an abstract for presentation, oral or written, to a learned society or symposium will be discussed on the Trial Management Group calls.

DocuSign Envelope ID: 3755EDA5-CDD3-4119-BD41-A6AF70DAAAAC

15 References

Breast Cancer Now. 2020. “Press Play: Getting and Keeping Breast Cancer Services Back on Track.” https://breastcancernow.org/sites/default/files/final_breast_cancer_now_press_play_report.pdf

Haenssle, Holger Andreas, Julia Katharina Winkler, Christine Fink, Ferdinand Toberer, Alexander Enk, Wilhelm Stolz, Teresa Deinlein, et al. 2020. “Skin Lesions of Face and Scalp - Classification by a Market-Approved Convolutional Neural Network in Comparison with 64 Dermatologists.” *European Journal of Cancer* 144 (December): 192–99.

Kiani, Amirhossein, Bora Uyumazturk, Pranav Rajpurkar, Alex Wang, Rebecca Gao, Erik Jones, Yifan Yu, et al. 2020. “Impact of a Deep Learning Assistant on the Histopathologic Classification of Liver Cancer.” *NPJ Digital Medicine* 3 (February): 23.

Macmillan Cancer Support. 2020. “The Forgotten ‘C’? The Impact of Covid-19 on Cancer Care.” https://www.macmillan.org.uk/assets/forgotten-c-impact-of-covid-19-on-cancer-care.pdf.

McKinney, Scott Mayer, Marcin Sieniek, Varun Godbole, Jonathan Godwin, Natasha Antropova, Hutan Ashrafian, Trevor Back, et al. 2020. “International Evaluation of an AI System for Breast Cancer Screening.” *Nature* 577 (7788): 89–94.

Moser, Kath, Sarah Sellars, Margot Wheaton, Julie Cooke, Alison Duncan, Anthony Maxwell, Michael Michell, et al. 2011. “Extending the Age Range for Breast Screening in England: Pilot Study to Assess the Feasibility and Acceptability of Randomization.” *Journal of Medical Screening* 18 (2): 96–102.

Public Health England. 2016. “NHS Breast Screening Programme: National Radiographic Workforce Survey 2016.” https://assets.publishing.service.gov.uk/government/uploads/system/uploads/attachment_data/file/564515/Final_radiographic_workforce_report_25-10-16_colinbabb_gateway_number_2016416.pdf.

Sounderajah, Viknesh, Hutan Ashrafian, Ravi Aggarwal, Jeffrey De Fauw, Alastair K. Denniston, Felix Greaves, Alan Karthikesalingam, et al. 2020. “Developing Specific Reporting Guidelines for Diagnostic Accuracy Studies Assessing AI Interventions: The STARD-AI Steering Group.” *Nature Medicine* 26 (6): 807–8.

The Royal College of Radiologists. 2021. “Clinical Radiology: UK Workforce Census 2020 Report.” https://www.rcr.ac.uk/system/files/publication/field_publication_files/clinical-radiology-uk-workforce-census-2020-report.pdf.

16 Appendix 1

16.1 De-identification of DICOM Images

Each medical image including mammograms is stored in a standard DICOM format. In addition to the actual image there is a DICOM header with information stored in fields that are known as “tags” which contain a very large amount of information including patient identifiable information. In order to preserve the confidentiality of the patients the following changes are made to each DICOM header at the clinical site before it is transferred to the central server.

All the tags not mentioned below are retained as specified in the DICOM supplement 142 standard for anonymisation as they do not contain patient data or data likely to permit patient data to be accessed.

16.1.1 Pseudonymisation of DICOM tags

The following tags are pseudonymised:

0010,0010: PatientName: This is replaced with an auto incrementing number

N/B: The auto incrementing can be global (i.e. across all institutes) or local (each institute has its own auto-incrementing number)

0010,0020: PatientID: This is changed to be the same as PatientName

0010,0030: PatientBirthDate: This is altered, so that the year remains the same, but the day and month are set to “01”

16.1.2 Private tags

All private tags are removed

16.1.3 UIDs

All UIDs are regenerated and replaced.

16.1.4 Nulling of DICOM tags

The following tags are either removed or nulled:

```
4008,0111 - Interpretation Approver Sequence
0018,9424 - Acquisition Protocol Description
0040,2010 - Order Callback Phone Number
4008,0300 - Impressions
4008,0118 - Results Distribution List Sequence
0400,0100 - Digital Signature UID
4008,0119 - Distribution Name
0012,0031 - Clinical Trial Site Name
0040,0253 - Performed Procedure Step ID
```

```
0012,0030 - Clinical Trial Site ID
0040,0254 - Performed Procedure Step Description
4008,0114 - Physician Approving Interpretation
60XX,4000
4008,0115 - Interpretation Diagnosis Description
0040,2016 - Placer Order Number / Imaging Service Request
6000,4000 - Overlay Comments
0012,0021 - Clinical Trial Protocol Name
0040,2009 - Order Enterer's Location
4008,011A - Distribution Address
0040,2008 - Order Entered By
0040,0248 - ?
0028,4000 - Image Presentation Comments
0040,A027 - Verifying Organization
4008,0202 - Interpretation ID Issuer
4008,0102 - Interpretation Recorder
0012,0062 - Patient Identity Removed
0012,0060 - Clinical Trial Coordinating Center Name
0012,0020 - Clinical Trial Protocol ID
0012,0010 - Clinical Trial Sponsor Name
0010,0010 - Patient's Name
0010,2299 - Responsible Organization
4008,010B - Interpretation Text
0040,2017 - Filler Order Number / Imaging Service Request
4008,010A - Interpretation Transcriber
0010,2297 - Responsible Person
4008,010C - Interpretation Author
0008,010D - Context Group Extension Creator UID
0040,1400 - Requested Procedure Comments
0070,0001 - Graphic Annotation Sequence
300A,0013 - Dose Reference UID
0040,0275 - Request Attributes Sequence
0012,0051 - Clinical Trial Time Point Description
0012,0050 - Clinical Trial Time Point ID
0010,0020 - Patient ID
0040,1004 - Patient Transport Arrangements
0040,1001 - Requested Procedure ID
0010,0021 - Issuer of Patient ID
0010,2180 - Occupation
0032,0012 - Study ID Issuer
0040,2400 - Imaging Service Request Comments
0040,0280 - Comments on the Performed Procedure Step
0040,000B - Scheduled Performing Physician Identification Sequence
0010,1000 - Other Patient IDs
0010,1001 - Other Patient Names
0020,9161 - Concatenation UID
0020,9164 - Dimension Organization UID
0040,1005 - Requested Procedure Location
0038,1234 - ?
0012,0040 - Clinical Trial Subject ID
0040,0006 - Scheduled Performing Physician's Name
0040,0007 - Scheduled Procedure Step Description
0040,2001 - Reason for the Imaging Service Request
0012,0042 - Clinical Trial Subject Reading ID
0040,0004 - Scheduled Procedure Step End Date
0040,0005 - Scheduled Procedure Step End Time
0032,1021 - Scheduled Study Location AE Title
0040,0012 - Pre-Medication
0040,1010 - Names of Intended Recipients of Results
0040,0011 - Scheduled Procedure Step Location
0032,1020 - Scheduled Study Location
0040,1011 - Intended Recipients of Results Identification Sequence
0010,0032 - Patient's Birth Time
0040,0010 - Scheduled Station Name
0010,0030 - Patient's Birth Date
0020,0052 - Frame of Reference UID
0040,A07C - Custodial Organization Sequence
```

```
0040,A07A - Participant Sequence
0008,2112 - Source Image Sequence
0040,A075 - Verifying Observer Name
0008,0096 - Referring Physician Identification Sequence
0008,0094 - Referring Physician's Telephone Numbers
0088,0910 - Topic Author
0040,A078 - Author Observer Sequence
0008,0092 - Referring Physician's Address
0088,0912 - Topic Keywords
0008,1060 - Name of Physician(s) Reading Study
0008,1062 - Physician(s) Reading Study Identification Sequence
0032,1030 - Reason for Study
0040,0001 - Scheduled Station AE Title
0008,2111 - Derivation Description
0032,1032 - Requesting Physician
0040,A073 - Verifying Observer Sequence
0032,1033 - Requesting Service
0040,0002 - Scheduled Procedure Step Start Date
0040,0003 - Scheduled Procedure Step Start Time
0010,1090 - Medical Record Locator
2030,0020 - Text String
0010,0102 - Patient's Primary Language Modifier Code Sequence
0008,0090 - Referring Physician's Name
0010,0101 - Patient's Primary Language Code Sequence
0020,4000 - Image Comments
0038,0400 - Patient's Institution Residence
0088,0906 - Topic Subject
0008,1195 - Transaction UID
0008,0080 - Institution Name
3006,00C2 - Related Frame of Reference UID
0008,0081 - Institution Address
0008,0082 - Institution Code Sequence
0008,1050 - Performing Physician's Name
0008,1052 - Performing Physician Identification Sequence
0020,0200 - Synchronization Frame of Reference UID
0008,0201 - Timezone Offset From UTC
4008,4000 - Results Comments
3006,0024 - Referenced Frame of Reference UID
0020,3406 - Modified Image Description
0020,3404 - Modifying Device Manufacturer
0008,1084 - Admitting Diagnoses Code Sequence
0020,3401 - Modifying Device ID
0040,0245 - Performed Procedure Step Start Time
0008,1080 - Admitting Diagnoses Description
0040,0244 - Performed Procedure Step Start Date
0040,0243 - Performed Location
0040,0242 - Performed Station Name
0040,0241 - Performed Station AE Title
0040,3001 - Confidentiality Constraint on Patient Data Description
0020,0010 - Study ID
0032,4000 - Study Comments
0020,0012 - Acquisition Number
0040,A088 - Verifying Observer Identification Code Sequence
0020,000D - Study Instance UID
0020,000E - Series Instance UID
0008,1072 - Operator Identification Sequence
0032,1060 - Requested Procedure Description
0008,1070 - Operators' Name
FFFA,FFFA - Digital Signatures Sequence
0008,0058 - Failed SOP Instance UID List
0038,0010 - Admission ID
0038,0011 - Issuer of Admission ID
0040,A730 - Content Sequence
0008,0050 - Accession Number
0010,1050 - Insurance Plan Identification
0038,0500 - Patient State
0038,001E - Scheduled Patient Institution Residence
```

0010,21B0 - Additional Patient History
0018,4000 - Acquisition Comments
0018,1030 - Protocol Name
0008,1155 - Referenced SOP Instance UID
0008,1010 - Station Name
0032,1070 - Requested Contrast Agent
0038,0021 - Admitting Time
0038,0020 - Admitting Date
0400,0403 - Referenced SOP Instance MAC Sequence
0008,4000 - Identifying Comments
0400,0402 - Referenced Digital Signature Sequence
0010,21C0 - Pregnancy Status
0010,1060 - Patient’s Mother’s Birth Name
0040,A124 - UID
0038,0300 - Current Patient Location
0040,A123 - Person Name
0010,2203 - Patient’s Sex Neutered
0010,2110 - Allergies
0008,1040 - Institutional Department Name
0018,1000 - Device Serial Number
0018,1002 - Device UID
0008,1049 - Physician(s) of Record Identification Sequence
0018,1005 - Generator ID
0018,1004 - Plate ID
0018,1007 - Cassette ID
0008,1048 - Physician(s) of Record
0008,1140 - Referenced Image Sequence
0018,1008 - Gantry ID
FFFC,FFFC - Data Set Trailing Padding
4008,0042 - Results ID Issuer
0018,0010 - Contrast/Bolus Agent
0070,0086 - Content Creator’s Identification Code Sequence
0070,0084 - Content Creator’s Name
0008,1030 - Study Description
0040,DB0D - Template Extension Creator UID
0040,DB0C - Template Extension Organization UID
0038,0040 - Discharge Diagnosis Description
0010,2000 - Medical Alerts
0088,0200 - Icon Image Sequence
0018,700A - Detector ID
0400,0561 - Original Attributes Sequence
0010,21A0 - Smoking Status
0088,0140 - Storage Media File-set UID
0038,4000 - Visit Comments
0010,1080 - Military Rank
0018,1400 - Acquisition Device Processing Description
0010,1081 - Branch of Service
4000,0010 - Arbitrary
0070,031A - Fiducial UID
0008,103E - Series Description
0010,1002 - Other Patient IDs Sequence
300E,0008 - Reviewer Name
0400,0550 - Modified Attributes Sequence
0008,1120 - Referenced Patient Sequence
0010,1005 - Patient’s Birth Name
0038,0050 - Special Needs
50XX,XXXX
0008,0018 - SOP Instance UID
0010,0040 - Patient’s Sex
0000,1001 - Requested SOP Instance UID
0008,0012 - Instance Creation Date
0008,0013 - Instance Creation Time
0008,0014 - Instance Creator UID
0008,2112 - Source Image Sequence
0020,9158 - Frame Comments
0040,0555 - Acquisition Context Sequence
0010,21F0 - Patient’s Religious Preference

```
0038,0060 - Service Episode ID
0010,1010 - Patient's Age
0008,3010 - Irradiation Event UID
4000,4000 - Text Comments
0008,1110 - Referenced Study Sequence
0028,1199 - Palette Color Lookup Table UID
0038,0062 - Service Episode Description
0002,0003 - Media Storage SOP Instance UID
0038,0061 - Issuer of Service Episode ID
0008,1111 - Referenced Performed Procedure Step Sequence
0010,0050 - Patient's Insurance Plan Code Sequence
0010,2160 - Ethnic Group
0040,4027 - Scheduled Station Geographic Location Code Sequence
0040,4028 - Performed Station Name Code Sequence
0040,0404 - ?
0040,4023 - Referenced General Purpose Scheduled Procedure Step Transaction UID
0040,4025 - Scheduled Station Name Code Sequence
0000,0021 - ?
0010,1020 - Patient's Size
0008,0030 - Study Time
0008,0031 - Series Time
0008,0032 - Acquisition Time
0018,A003 - Contribution Description
0010,2150 - Country of Residence
0008,0033 - Content Time
0008,0034 - Overlay Time
0008,9123 - Creator-Version UID
0010,2152 - Region of Residence
0008,0035 - Curve Time
0010,2154 - Patient's Telephone Numbers
0040,4036 - Human Performer's Organization
0040,4035 - Actual Human Performers Sequence
0040,4034 - Scheduled Human Performers Sequence
0040,4037 - Human Performer's Name
0010,21D0 - Last Menstrual Date
0040,4030 - Performed Station Geographic Location Code Sequence
0010,1030 - Patient's Weight
60XX,3000
0008,0021 - Series Date
0040,1103 - Person's Telephone Numbers
0008,0024 - Overlay Date
0008,0025 - Curve Date
0040,1102 - Person's Address
0008,0022 - Acquisition Date
0040,1101 - Person Identification Code Sequence
0008,0023 - Content Date
0008,002A - Acquisition DateTime
0008,1511 - ?
0010,1040 - Patient's Address
```
