# Supplementary material for: Diagnostic accuracy, fairness and clinical implementation of AI for breast cancer screening: results of multicenter retrospective and prospective technical feasibility studies
Source: Nat Cancer. 2026 Mar 10;7(3):494–506. doi: 10.1038/s43018-026-01127-0 (PMC13035471; doi:10.1038/s43018-026-01127-0)
Supplement: Supplementary file 2 — Reporting Summary [file 43018_2026_1127_MOESM2_ESM.pdf]

# nature portfolio

Corresponding author(s): Christopher Kelly
Last updated by author(s): 10/30/2025

# Reporting Summary

Nature Portfolio wishes to improve the reproducibility of the work that we publish. This form provides structure for consistency and transparency in reporting. For further information on Nature Portfolio policies, see our Editorial Policies and the Editorial Policy Checklist.

## Statistics

For all statistical analyses, confirm that the following items are present in the figure legend, table legend, main text, or Methods section.

| n/a | Confirmed | |
|---|---|---|
| ☐ | ☒ | The exact sample size (*n*) for each experimental group/condition, given as a discrete number and unit of measurement |
| ☒ | ☐ | A statement on whether measurements were taken from distinct samples or whether the same sample was measured repeatedly |
| ☐ | ☒ | The statistical test(s) used AND whether they are one- or two-sided<br>*Only common tests should be described solely by name; describe more complex techniques in the Methods section.* |
| ☒ | ☐ | A description of all covariates tested |
| ☐ | ☒ | A description of any assumptions or corrections, such as tests of normality and adjustment for multiple comparisons |
| ☐ | ☒ | A full description of the statistical parameters including central tendency (e.g. means) or other basic estimates (e.g. regression coefficient) AND variation (e.g. standard deviation) or associated estimates of uncertainty (e.g. confidence intervals) |
| ☐ | ☒ | For null hypothesis testing, the test statistic (e.g. *F*, *t*, *r*) with confidence intervals, effect sizes, degrees of freedom and *P* value noted<br>*Give P values as exact values whenever suitable.* |
| ☒ | ☐ | For Bayesian analysis, information on the choice of priors and Markov chain Monte Carlo settings |
| ☒ | ☐ | For hierarchical and complex designs, identification of the appropriate level for tests and full reporting of outcomes |
| ☒ | ☐ | Estimates of effect sizes (e.g. Cohen's *d*, Pearson's *r*), indicating how they were calculated |

*Our web collection on statistics for biologists contains articles on many of the points above.*

## Software and code

Policy information about availability of computer code

| | |
|---|---|
| Data collection | AI analysis performed by Google's Breast Cancer AI software v1.2, Google LLC. |
| Data analysis | Statistical analysis and plotting was performed using open source and publicly available Python packages (numpy v2.3.3, pandas v2.3.2, scipy v1.16.1, sklearn v1.7.1, matplotlib v3.9.1), using the methodology outlined in the Methods section. All code was executed in Python (v.2.7.18) |

For manuscripts utilizing custom algorithms or software that are central to the research but not yet described in published literature, software must be made available to editors and reviewers. We strongly encourage code deposition in a community repository (e.g. GitHub). See the Nature Portfolio guidelines for submitting code & software for further information.

## Data

Policy information about availability of data

All manuscripts must include a data availability statement. This statement should provide the following information, where applicable:

- Accession codes, unique identifiers, or web links for publicly available datasets
- A description of any restrictions on data availability
- For clinical datasets or third party data, please ensure that the statement adheres to our policy

All mammography images and metadata collected during the course of the retrospective study are now available via the OPTIMAM database. Applications for access from healthcare institutions, academic centres or commercial organisations are considered by OPTIMAM and access is subject to a data sharing agreement.

More details are available at https://medphys.royalsurrey.nhs.uk/omidb/getting-access/. Source data for all graphs within the Figures and Extended Data Figures have been provided as Source Data files. All other data supporting the findings of this study are available from the corresponding authors on reasonable request.

## Research involving human participants, their data, or biological material

Policy information about studies with human participants or human data. See also policy information about sex, gender (identity/presentation), and sexual orientation and race, ethnicity and racism.

| | |
|---|---|
| Reporting on sex and gender | This study is focused on population breast cancer screening - something that is only available to females. Therefore the study only analysed data from women. |
| Reporting on race, ethnicity, or other socially relevant groupings | We report subgroup analyses on ethnicity (as defined by the UK government - https://www.ethnicity-facts-figures.service.gov.uk/style-guide/ethnic-groups/) and rely on routinely collected NHS data for these analyses. We also analyse results using Indices of Multiple Deprivation as a measure of socioeconomic status (https://data.cdrc.ac.uk/dataset/index-multiple-deprivation-imd) and calculate this using postcode data prior to anonymisation. |
| Population characteristics | 125,000 women aged 50-70 who underwent routine screening at five screening services during 2016, and who had a subsequent screening attendance between 24-39 months or a documented interval cancer within 39 months. |
| Recruitment | Retrospective study: Random selection from the population who underwent screening at at five screening services: Cornwall Breast Screening Service, Leicester & Rutland Breast Screening Service, Oxford Breast Imaging Centre, South West London Breast Screening Service (SWLBSS), and West of London Breast Screening Service (WoLBSS).<br><br>Prospective: Women aged 50-70 were consecutively recruited if they underwent routine screening as part of national breast screening during study dates 27 Nov 2023 - 19 Jan 2024 (West of London Breast Screening Service) or 4 Dec 2023 - 9 Feb 2024 (South West London Breast Screening Service). The study had national ethical approval for opt-out consent, meaning that informed consent was not needed to use this routinely collected anonymised data. |
| Ethics oversight | The retrospective study protocol was approved by East Midlands Nottingham Research Ethics Committee (22/EM/0038) and NHS England Breast Screening Programme Research Advisory Committee (BSPRAC_0093). The prospective study protocol was approved by East Midlands Nottingham Research Ethics Committee (22/EM/0198), NHS England (NHSE) Breast Screening Programme (BSP) Research Innovation and Development Advisory Committee (BSPRAC_0093b), and NHS England's Research Advisory Committee (BSPRAC_0093). NHS Confidentiality Advisory Group approved the study for an opt-out consent approach under section 251 of NHS Act 2006 (22/CAG/0124). |

Note that full information on the approval of the study protocol must also be provided in the manuscript.

# Field-specific reporting

Please select the one below that is the best fit for your research. If you are not sure, read the appropriate sections before making your selection.

☒ Life sciences ☐ Behavioural & social sciences ☐ Ecological, evolutionary & environmental sciences

For a reference copy of the document with all sections, see nature.com/documents/nr-reporting-summary-flat.pdf

# Life sciences study design

All studies must disclose on these points even when the disclosure is negative.

| | |
|---|---|
| Sample size | 125,000 participants. The study's primary endpoint was powered at a site level with a target power of at least 80%. This required approximately 25,000 women per site, assuming a population prevalence of 200 cancer cases per site. |
| Data exclusions | As described in Extended Data Figure 1 (Data flow diagram), exclusions included cases that failed processing, cases that were "technical recalls" (i.e. cases that required re-imaging due to poor quality), cases with a non-standard number of views (bespoke approach required), and cases that had implants. Negative cases without a subsequent negative screening attendance between 24-39 months later were excluded as the lack of confirmation prevented a robust ground truth for evaluation. |
| Replication | The study was essentially replicated by the inclusion of 5 separate sites, and these per-site results are included in the manuscript. The AI model is deterministic, and so produces the same result when run twice on the same case. |
| Randomization | This study did not randomise cases into different experimental groups. |
| Blinding | This was not an interventional study, and so blinding was not relevant. |

# Reporting for specific materials, systems and methods

We require information from authors about some types of materials, experimental systems and methods used in many studies. Here, indicate whether each material, system or method listed is relevant to your study. If you are not sure if a list item applies to your research, read the appropriate section before selecting a response.

## Materials & experimental systems

| n/a | Involved in the study |
|---|---|
| ☒ | ☐ Antibodies |
| ☒ | ☐ Eukaryotic cell lines |
| ☒ | ☐ Palaeontology and archaeology |
| ☒ | ☐ Animals and other organisms |
| ☐ | ☒ Clinical data |
| ☒ | ☐ Dual use research of concern |
| ☒ | ☐ Plants |

## Methods

| n/a | Involved in the study |
|---|---|
| ☒ | ☐ ChIP-seq |
| ☒ | ☐ Flow cytometry |
| ☒ | ☐ MRI-based neuroimaging |

# Clinical data

Policy information about clinical studies

All manuscripts should comply with the ICMJE guidelines for publication of clinical research and a completed CONSORT checklist must be included with all submissions.

**Clinical trial registration**

The retrospective study was registered with ISRCTN (60839016). The prospective study was registered with ISRCTN (88754382).

**Study protocol**

The study protocol has been provided along with the manuscript file.

**Data collection**

Our planned selection process for this study was to select a random selection of women who had attended screening in 2016. This would permit 3 years follow up in 2019, before COVID-19 disrupted screening practices in 2020. Women aged 67 without 3 years' follow-up (39 months, to allow 3 months for slippage of the ideal 3 year interval) were replaced with women with 3 year follow-up examination, matched by age and ethnicity. Women aged 68+ were permitted to have no follow up screen, as they would not typically be invited back as part of national screening at this age.

**Outcomes**

Primary endpoints for the study from the protocol:
● AI system cancer detection sensitivity and specificity (diagnostic accuracy matrix) compared to first, second and consensus reader decisions, measured against ground truth definition above.
Secondary endpoints will include:
● Subgroup performance by factors including cancer type and grade, primary tumour size, patient age, breast density, prior cancer, prevalent and incident screens, ethnicity, device manufacturer, socioeconomic status, and screening site.
● AUC-ROC for cancer detection, positive predictive value, negative predictive value, cancer detection rate, case recall rate.
● System performance in confirmed interval cancers (percentage of historical interval cancers that the AI system flagged for recall, and qualitative agreement of the localisation in the original screening mammogram with the presence/absence of true radiological evidence).
● AI system localisation performance (if lesion position data available).
● Analysis of failure cases.
● Percentage of women that meet the eligibility criteria.
● Simulations of workforce impact assessment and health economic modelling.
For prospective study:
● To demonstrate successful technical integration
● To assess automated eligibility checks

# Plants

**Seed stocks**

*Report on the source of all seed stocks or other plant material used. If applicable, state the seed stock centre and catalogue number. If plant specimens were collected from the field, describe the collection location, date and sampling procedures.*

**Novel plant genotypes**

*Describe the methods by which all novel plant genotypes were produced. This includes those generated by transgenic approaches, gene editing, chemical/radiation-based mutagenesis and hybridization. For transgenic lines, describe the transformation method, the number of independent lines analyzed and the generation upon which experiments were performed. For gene-edited lines, describe the editor used, the endogenous sequence targeted for editing, the targeting guide RNA sequence (if applicable) and how the editor was applied.*

**Authentication**

*Describe any authentication procedures for each seed stock used or novel genotype generated. Describe any experiments used to assess the effect of a mutation and, where applicable, how potential secondary effects (e.g. second site T-DNA insertions, mosiacism, off-target gene editing) were examined.*
